# An improved golden jackal optimization for multilevel thresholding image segmentation

**DOI:** 10.1371/journal.pone.0285211

**Published:** 2023-05-05

**Authors:** Zihao Wang, Yuanbin Mo, Mingyue Cui, Jufeng Hu, Yucheng Lyu

**Affiliations:** 1 School of Artificial Intelligence, Guangxi Minzu University, Nanning, China; 2 Guangxi Key Laboratory of Hybrid Computation and IC Design Analysis, Guangxi Minzu University, Nanning, China; Universidad de Guadalajara, MEXICO

## Abstract

Aerial photography is a long-range, non-contact method of target detection technology that enables qualitative or quantitative analysis of the target. However, aerial photography images generally have certain chromatic aberration and color distortion. Therefore, effective segmentation of aerial images can further enhance the feature information and reduce the computational difficulty for subsequent image processing. In this paper, we propose an improved version of Golden Jackal Optimization, which is dubbed Helper Mechanism Based Golden Jackal Optimization (HGJO), to apply multilevel threshold segmentation to aerial images. The proposed method uses opposition-based learning to boost population diversity. And a new approach to calculate the prey escape energy is proposed to improve the convergence speed of the algorithm. In addition, the Cauchy distribution is introduced to adjust the original update scheme to enhance the exploration capability of the algorithm. Finally, a novel “helper mechanism” is designed to improve the performance for escape the local optima. To demonstrate the effectiveness of the proposed algorithm, we use the CEC2022 benchmark function test suite to perform comparison experiments. the HGJO is compared with the original GJO and five classical meta-heuristics. The experimental results show that HGJO is able to achieve competitive results in the benchmark test set. Finally, all of the algorithms are applied to the experiments of variable threshold segmentation of aerial images, and the results show that the aerial photography images segmented by HGJO beat the others. Noteworthy, the source code of HGJO is publicly available at https://github.com/Vang-z/HGJO.

## 1. Introduction

Aerial imagery is an important component of modern photography and scientific research. Through aerial photography technology, we can obtain high-resolution images of natural landscapes and urban architecture with previously unparalleled accuracy and detail. These images can be used in various fields such as map drawing [1], urban planning [2], land use planning [3], environmental monitoring [4], and agricultural and forestry resource management [5]. However, these high-resolution images may suffer from distortion due to external environmental factors, which can make subsequent work difficult. Therefore, preprocessing aerial images is particularly important.

By segmenting, denoising, enhancing, and other operations on aerial images, we can effectively improve the quality and accuracy of the images, providing more reliable data support for subsequent applications. On the other hand, segmentation technology can also reduce the complexity of high-resolution images, making subsequent processing more efficient and accurate. Image segmentation is one of the most critical processes in computer vision. Preprocessing through segmentation technology can effectively reduce the complexity of subsequent processes.

Segmentation technology is the process of dividing images into several categories based on pixels, with threshold segmentation being the most effective and commonly used method [6]. Due to its simplicity and stable performance, threshold segmentation has always been the preferred segmentation technology [7]. In addition, there are clustering-based [8], edge-based [9], and region-based [10] segmentation methods that have also attracted the attention of researchers. However, these methods are currently only effective for binary segmentation problems. As the number of segmentation targets increases beyond two, their processing capabilities decrease exponentially. This phenomenon is due to the exponential growth in complexity of multi-threshold image segmentation problems compared to binary segmentation, making traditional segmentation methods unable to calculate feasible solutions within a limited time frame. Therefore, researchers have focused their attention on metaheuristics for these types of problems. In recent years, some of the most popular meta-heuristics used to solve engineering problems include Genetic Algorithms (GA) [11], Particle Swarm Optimization (PSO) [12], Differential Evolution (DE) [13], Ant Colony Optimization (ACO) [14], Whale Optimization Algorithm (WOA) [15], and Grey Wolf Optimization (GWO) [16]. In addition, some recently proposed metaheuristics have also been widely recognized by researchers, such as Arithmetic Optimization Algorithm [17], Aquila Optimizer (AO) [18], Ebola Optimization Search Algorithm (EOSA) [19], Dwarf Mongoose Optimization Algorithm (DMO) [20], Reptile Search Algorithm (RSA) [21], Prairie Dog Optimization Algorithm (PDO) [22], and Gazelle Optimization Algorithm (GOA) [23]. These algorithms have achieved good results in dealing with real word engineering problems, providing new ideas for the development of multi-threshold image segmentation. The following is a summary of the meta-heuristics used by scholars in various fields to solve multilevel segmentation problems in recent years.

Yin [24] proposed a recursive method to optimize the minimum cross entropy thresholding (MCET), which is combined with particle swarm optimization to search for the near-optimal MCET. The experimental results demonstrate that the MCET obtained by the proposed algorithm is very close to the optimal MCET. Osuna-Enciso, Cuevas and Sossa [25] presented a mixture of Gaussian functions to approximate the 1D histogram of a gray level image, which is combined with Particle Swarm Optimization, Artificial Bee Colony Optimization, and Differential Evolution. The experimental outcomes indicate that each algorithm has its own advantages and disadvantages, and all of them could achieve more satisfactory results. Gao, Kwong, Yang and Cao [26] suggested a novel Particle Swarm Optimization with an intermediate perturbation search strategy, which is called IDPSO. The proposed algorithm is compared with 10 different improved PSO algorithms which achieved outstanding performance on multilevel threshold segmentation problems. Jiang, Yeh, Hao and Yang [27] introduced a new hybrid algorithm which is based on honey bee mating algorithms and cooperative learning, furthermore a new initialization population strategy is employed. Upon extensive experimental analysis, the algorithm is demonstrated to be able to be applied to complex image processing problems. Han, Yang, Zhou and Gui [28] used the State Transfer Algorithm (STA) to fit the optimal parameters of a linear combined normal distribution functions. The competitive capability of the STA in thresholding segmentation is illustrated by comparison with the classical meta-heuristics. He and Huang [29] proposed an effective improved multilevel color image thresholding firefly algorithm using Kapur’s entropy, minimum cross-entropy and inter-class variance method as the objective function. The experimental outcomes indicate that the proposed algorithm is superior to other classical metaheuristic algorithms in all aspects. Ishak [30] developed a two-dimensional multilevel thresholding technique based on Rényi and Tsallis entropies, which combines Quantum Genetic Algorithm and Differential Evolutionary to solve the segmentation problem of multimodal noisy images.

A multi-level threshold image segmentation method using the Fruit Fly Optimization Algorithm was developed by Ding, Dong and Zou [31]. Extensive experimental indicate that the proposed algorithm could significantly reduce the time cost and also achieve satisfactory computational accuracy. To effectively segment coastal video images, a multilevel thresholding method based on Cuckoo Search Algorithm was designed by Widyantara et al [32]. This method was successful in overcoming a series of problems caused by nonlinear variations in image quality and opaque areas. Bohat and Arya [33] propose a novel threshold heuristic algorithm for the multilevel thresholding problem, which embeds the proposed algorithm into the Whale Optimization Algorithm, Gray Wolf Optimizer and Particle Swarm Optimization. Experiments illustrate that this work reduced the computation time of all the embedded algorithms. Singh, Mittal and Singh [34] presented an efficient multilevel thresholding image segmentation method based on Learning enthusiasm-based Teaching-Learning-Based Optimization. This method conquered the problem with increasing the level of redundant thresholds makes the computational complexity grow exponentially. Qualitative experimental outcomes demonstrate that the proposed algorithm is efficient in the field of image segmentation. Xing [35] proposed a novel color image segmentation method based on Emperor Penguin Optimization for Berkeley images, Satellite images, and Plant canopy images. The experimental shows that the method has superior segmentation accuracy. In addition, the computational complexity does not increase exponentially due to the increase of thresholds. Upadhyay and Chhabra [36] suggested a Kapur’s entropy based Crow Search Algorithm to solve the optimal solution for multilevel thresholding image segmentation. By comparing with the classical meta-heuristics, the proposed algorithm achieves satisfactory performance with respect to both quality and consistency. Mousavirad and Ebrahimpour-Komleh [37] proposed a multilevel thresholding for image segmentation using Human Mental Search. This method combines Kapur’s entropy and Otsu method to achieve significant advantages in multi-threshold segmentation problems.

Zhao et al. [38] proposed an improved Slime Mould Algorithm, which introduced a diffusion mechanism to increase the diversity of population. In addition, this methodology was successfully applied to the CT image segmentation of chronic obstructive pulmonary disease, which could help physicians to analyze the lesion tissues qualitatively and quantitatively, moreover improving the accuracy of diagnosis. Swain et al. [39] developed a multilevel thresholding image segmentation method based on differential exponential entropy. The method was combined with Equilibrium-Cuckoo Search Optimizer to achieve satisfactory performance in satellite image segmentation. Furthermore, the method is suggested in the paper for segmentation of the brain MR images. Houssein et al. [40] proposed a fresh approach based on the black widow optimizer to overcome the problem of high computational cost of multilevel thresholding image segmentation. This method has been compared with six well-known meta-heuristics. The comparison reveals that this method is the most potential alternative. Ma and Yue [41] proposed an improved multilevel thresholding image segmentation method based on the Whale Optimization Algorithm. The proposed method obtains satisfactory results for image segmentation in both grayscale and color images, respectively. Emam et al. [42] proposed an enhanced RSA algorithm for global optimization and image segmentation, which overcomes the tendency of RSA to get stuck in local optima by combining it with the RUNge Kutta Optimizer (RUN) [43] and applies it to brain MRI image segmentation. The results showed that it outperformed other advanced meta-heuristics in terms of segmentation accuracy and computational efficiency. To further advance research on COVID-19, Houssein et al. [44] used the opposition-based learning mechanism to improve the Manta Ray Foraging Optimization. Experimental results showed that the proposed method has higher robustness compared to existing meta-heuristics. Additionally, in [45], the Equilibrium Optimizer was further improved to advance research on COVID-19, and experimental results showed that the proposed method can be an effective tool for image segmentation. These two works further advance the research on image segmentation for COVID-19, making effective contributions to prevent the spread of COVID-19. In addition, Houssein et al. [46] introduced the opposition-based learning strategy into the Marine Predator Algorithm (MPA) [47] to accelerate the convergence speed of MPA. Finally, Otsu and Kapur entropy were used as objective functions to perform segmentation experiments on benchmark images. The experimental results showed that the proposed algorithm outperformed other methods.

In [48], the authors used an improved Golden Jackal Optimization (GJO) [49] to segment skin cancer images, enhancing the original GJO algorithm using the opposition-based learning and comparing it with seven different meta-heuristics. The experimental results showed that the proposed method outperformed other alternative algorithms and effectively solved the segmentation problem. However, the improvement of this work is limited for the GJO algorithm, as the time complexity of thresholding increases exponentially with increasing image resolution, and the single opposition-based learning mechanism cannot achieve satisfactory results on high-resolution images. Although meta-heuristics have been widely used in image segmentation, there are still shortcomings in multi-level threshold image segmentation for complex images. In other words, researchers are currently working on developing a method that can maintain consistent results when dealing with complex problems. The GJO algorithm is a novel and highly scalable swarm intelligence optimization algorithm proposed in 2022, which has been widely used by scholars [50–52]. Therefore, in this paper, we propose an efficient image segmentation method based on the GJO algorithm to further advance multi-level threshold segmentation work at high resolution and apply it to aerial image segmentation.

In this study, we make further improvements to the original GJO to enhance its potential in multi-level thresholding image segmentation. In order to evaluate the effectiveness of the improvements, the proposed algorithm is compared with numerous classical and novel algorithms on CEC2022 benchmark functions. Moreover, we used Peak Signal to Noise Ratio (*PSNR*) [53], Structural Similarity Index (*SSIM*) [54], and Feature Similarity Index (*FSIM*) [55] to determine the performance of image segmentation. The main contributions of this study are as follows:

1. The Opposition-Based Learning (OBL) strategy is integrated into the initialization of the GJO. The OBL strategy could dramatically improve the quality of the candidate solution to escape from the local optimal solution.
2. The Cauchy distribution is introduced to enhance the raw single Lévy flight, strengthening the distributivity of the population to improve the capability of the algorithm to search the global optimum. Furthermore, a new approach to calculate the prey escape energy is proposed. It is a more reasonable nonlinear method of calculation, which leads to a better balance of exploration and exploitation.
3. “Helpers” are introduced to improve the performance of GJO for the first time. They are some special individuals of the golden jackal population. The overall disturbance of the population by the “Helpers” before the end of each iteration can effectively prevent the algorithm trapped into local optimum.
4. The proposed HGJO was compared with numerous classical and novel algorithms on CEC2022 benchmark functions, and a lot of the segmentation of aerial images. The outcomes demonstrate that the proposed HGJO has remarkably superior performance and enables to challenge the current existing algorithms.

The rest of this paper is structured as follows: Section 2 encompasses a review of the multilevel threshold segmentation and the original GJO algorithm. The Improved Golden Jackal optimization is proposed in Section 3. Section 4 introduces, discusses, and analyzes the results of CEC2022 benchmark functions. Section 5 investigates the performance of the segmentation of aerial images. Finally, Section 6 concludes by summarizing the research and making recommendations for future work.

## 2. Literature review

### 2.1.Multilevel thresholding image segmentation

Threshold segmentation, as the name implies, is the division of an image into two parts based on the pixel values, with a given threshold. However, for the current needs of computer vision tasks, it is often not enough to simply segment the image into two parts. Therefore, depending upon the current requirements, most scholars are investigating multilevel thresholding. In general, multilevel thresholding is the addition of more thresholds to binary thresholding to segment the image into more units. At the current stage, the most commonly used thresholding method for segmentation is the Otsu method. The Otsu method involves histogram of the image as the input, where the generated class information is employed to calculate the optimal threshold for segmenting the image. The Otsu method was firstly proposed by Otsu in 1979 [56] to segment the grey scale image by maximizing the variance between classes. The approach considers *L* to represent the different gray levels in an image which has the size of M*N.

$$n=n_{0}+n_{1}+\ldots +n_{L-1}$$ (1)

$$p_{i}=\frac{n_{i}}{n},\;\sum_{i=0}^{L-1}p_{i}=1$$ (2)

where *n* means the total number of pixels in the image, *n*_*i*_ denotes the number of pixels for gray level *i*, and *p*_*i*_ indicates the probability distribution of gray levels.

Suppose there is a threshold *k*, in which 0<*k*<*L*−1, then the current input image will be segmented into two classes, namely *C*_1_ and *C*_2_, where *C*_1_ and *C*_2_ contain all pixels with the grayscale in [0, *k*] and [*k*+1, *L*−1], respectively.

$$P_{1}(k)=\sum_{i=0}^{k}p_{i},\;P_{2}(k)=\sum_{i=k+1}^{L-1}p_{i}=1-P_{1}(k)$$ (3)

where *P*_1_(*k*) and *P*_2_(*k*) represent the probability of a pixel has been classified into *C*_1_ or *C*_2_, respectively.

$$m_{1}(k)=\sum_{i=0}^{k}iP(i|C_{1})=\sum_{i=0}^{k}\frac{iP(C_{1}|i)P(i)}{P(C_{1})}=\frac{1}{P_{1}(k)}\sum_{i=0}^{k}ip_{i}$$ (4)

$$m_{2}(k)=\sum_{i=k+1}^{L-1}iP(i|C_{2})=\sum_{i=k+1}^{L-1}\frac{iP(C_{2}|i)P(i)}{P(C_{2})}=\frac{1}{P_{2}(k)}\sum_{i=k+1}^{L-1}ip_{i}$$ (5)

$$m_{k}=\sum_{i=0}^{k}ip_{i},\;m_{G}=\sum_{i=0}^{L-1}ip_{i}$$ (6)

where *m*_1_(*k*) and *m*_2_(*k*) indicate the average gray value of the pixels in *C*_1_ and *C*_2_, respectively. *m*_*k*_ denotes the average grayscale from 0 to *k*. *m*_*G*_ represents the average grayscale of the whole image. Hence, we can derive Eq (7) without ambiguity. Then the between class variance can be expressed as Eq (8).


$$\begin{array}{l} P_{1}(k)\cdot m_{1}(k)+P_{2}(k)\cdot m_{2}(k)=m_{G}\\ P_{1}(k)+P_{2}(k)=1 \end{array}$$ (7)

$$\begin{array}{l} \sigma_{B}^{2}=\frac{{\left(m_{G}P_{1}(k)-m_{k}\right)}^{2}}{P_{1}(k)(1-P_{1}(k))}\\ \;=P_{1}(k)(m_{1}(k)-m_{G}{)}^{2}+P_{2}(k)(m_{2}(k)-m_{G}{)}^{2}\\ \;=P_{1}(k)P_{2}(k)(m_{1}(k)-m_{2}(k){)}^{2} \end{array}$$ (8)

$$\sigma_{B}^{2}(k^{*})=\underset{0\leq k\leq L-1}{\max}\sigma_{B}^{2}(k)$$ (9)

As shown by Eq (8), we are able to determine a k* to make the maximum of $\sigma_{B}^{2}$, which is denoted as Eq (9). Therefore, the Otsu method can be used as an objective function of an optimization problem to solve the optimal threshold value for segmented images.

### 2.2.Golden jackal optimization

GJO is a novel metaheuristic algorithm proposed by Chopra and Ansari in 2022 [49]. GJO simulates the behavior of the golden jackal in natural environments for hunting. The search agents of this algorithm follow male and female jackals to seek, encircle and attack the prey, while the male jackal is considered as the global optimal solution of the problem. The entire description of the GJO is given below:

#### 1. Initialization

As mentioned above, GJO is a population-based meta-heuristic. Therefore, the initialization of GJO is consistent with most meta-heuristics. The process of the initialization is described in detail in Eq (10).

$$\begin{array}{l} \vec{X_{k}}=\vec{LB}+\vec{r}\cdot (\vec{UB}-\vec{LB}),\;k=1,2,\ldots ,n\\ X={\left[\vec{X_{1}},\vec{X_{2}},\ldots ,\vec{X_{n}}\right]}^{T} \end{array}$$ (10)

where *X* denotes the prey matrix, $\vec{X_{k}}$ indicates the position of the prey, *n* represents the size of population, $\vec{LB}$ and $\vec{UB}$ stand for the lower and upper boundary, respectively. $\vec{r}$ is a random vector between 0 and 1.

**Algorithm 1** Pseudo-code of the GJO

**Inputs**: The Population Size *N* and the Max iterations *T*.

**Outputs**: The best solution.

1. Initialization the population *X*.

2. **while** (*t*<*T*)

3. Calculating the fitness of the population.

4. $\vec{X_{M}}(t)=best(X(t))$

5. $\vec{X_{FM}}(t)=second\_best(X(t))$

6. **foreach** (*X*(*t*))

7.  Update the Evasion Energy (*E*) according Eq (13).

8.  if (|*E*|≥1)

9.   Update the population according Eqs (11) and (12).

10.  **else**

11.   Update the population according Eqs (14) and (12).

12.  **end if**

13.  **end foreach**

14. *t* = *t*+1

15. **end while**

16. **return** the best solution ($\vec{X_{M}}$)

#### 1. Exploration

In the exploration phase, the algorithm will search for as many potential solutions as possible over the search space. The position update is performed by a male jackal leading a female jackal, which is shown in the following mathematical model:

$$\begin{array}{l} \vec{X_{1}}=\vec{X_{m}}(t)-\vec{E}\cdot |\vec{X_{m}}(t)-\vec{RL}\cdot \vec{X_{k}}(t)|\\ \vec{X_{2}}=\vec{X_{fm}}(t)-\vec{E}\cdot |\vec{X_{fm}}(t)-\vec{RL}\cdot \vec{X_{k}}(t)| \end{array}$$ (11)

$$\vec{X_{k}}(t+1)=\frac{\vec{X_{1}}+\vec{X_{2}}}{2}$$ (12)

where *t* is the current iteration, $\vec{X_{k}}(t+1)$ stand for the position after it has been updated, $\vec{X_{k}}(t)$ means the current position. $\vec{X_{M}}(t)$ and $\vec{X_{FM}}(t)$ denote the current positions of the male and female jackals, respectively, which are best and second-best fitness of the population. $\vec{RL}$ is a random vector which is based on Lévy motion. $\vec{E}$ is the Evasion Energy of prey which is calculated as follows:

$$\vec{E}=E_{1}\cdot \vec{E_{0}},\;E_{1}=1.5\times (1-t/T),\;\vec{E_{0}}=2\cdot \vec{r}-1$$ (13)

where *E*_1_ is the decreasing energy of the prey, $\vec{E_{0}}$ is the initial energy of the prey. *T* stands for the maximum iterations.

#### 1. Exploitation

As most of the meta-heuristics, the exploitation phase is based on the exploration phase. Through the exploitation of the candidate solutions which have been searched, the global optimal solution is approximated as closely as possible. The mathematical is shown as follows:

$$\begin{array}{l} \vec{X_{1}}=\vec{X_{m}}(t)-\vec{E}\cdot |\vec{RL}\cdot \vec{X_{m}}(t)-\vec{X_{k}}(t)|\\ \vec{X_{2}}=\vec{X_{fm}}(t)-\vec{E}\cdot |\vec{RL}\cdot \vec{X_{fm}}(t)-\vec{X_{k}}(t)| \end{array}$$ (14)

It is worth noting that all of the variables are given in 2.2.2. Therefore, we do not repeat them. In addition, the pseudo-code of the GJO is shown in Algorithm 1.

## 3. Improved golden jackal optimization

### 3.1.Opposition-based learning strategy

Since the generation for initial solution of GJO is consistent with most meta-heuristics, this approach may cause the initial population to be unevenly distributed and converge sluggishly. However, the performance of the algorithm is strongly influenced by the initial population. A high-quality initial population not only improves the convergence speed of the algorithm but even has the potential to determine the final outcomes. Enhancing population diversity can effectively avoid the algorithm from maturing prematurely and falling into a local optimum. Therefore, the Opposition-Based Learning(OBL) [57] strategy is used to assist with the generation of the initial population in this study. Each individual in the population was given an Opposition solution to select the better individual as the initial solution, which could improve the convergence performance of GJO. The mathematical model of the OBL is shown as follows:

$$\tilde{X_{k}}=\vec{LB}+\vec{UB}-\vec{X_{k}},k=1,2,\ldots n$$ (15)

where $\tilde{X_{i}}$ is the opposing individual of $\vec{X_{k}}$ in the search space. If the fitness of $\tilde{X_{k}}$ is better than $\vec{X_{k}}$, then $\tilde{X_{k}}$ will be retained as the initial individual.

### 3.2.Cauchy distribution and dynamic balance strategy

#### 3.2.1.Cauchy distribution

**Fig 1:**
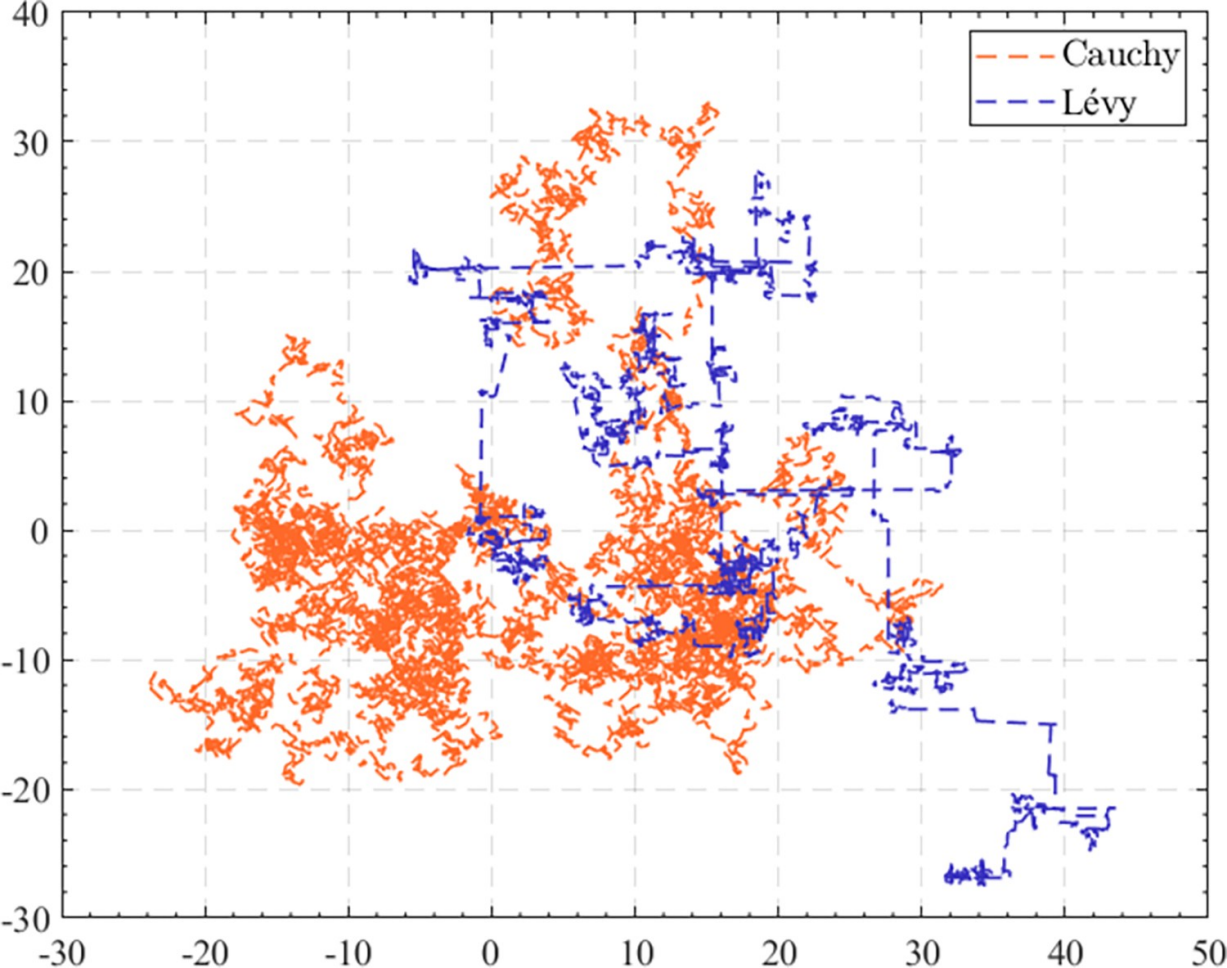
The movement of Cauchy distribution and Lévy flight.

The Cauchy distribution is a continuous probability distribution without mathematical expectation. Better outcomes tended to be achieved when the motion state of the population was portrayed by the Cauchy distribution [58]. Fig 1 is a comparison of the motion trajectory employing the Cauchy distribution and the Lévy flight, which can be visualized that the Cauchy distribution is able to perform a comprehensive search in a given search space. Therefore, it is a more sensible choice to adopt the Cauchy distribution in the exploration stages. The probability density function of the Cauchy distribution is shown below:

$$f(x;x_{0},\gamma )=1/\pi \gamma [1+{\left(\frac{x-x_{0}}{\gamma }\right)}^{2}]=\frac{1}{\pi }[\frac{\gamma }{{\left(x-x_{0}\right)}^{2}+\gamma^{2}}]$$ (16)

where *x*_0_ represents the position parameter, specifying the position of the peak of the distribution. *γ* denotes the scale parameter, which specifies the half-width at half-maximum. The Cauchy distribution which obeys *X*~*C*(0, 0.5) is utilized in this work. Where *γ* is fixed at 0.5 was determined by experimental analysis. Table 1 shows the effect of the algorithm using different *γ* on the test results of IEEE CEC 2022. In this experiment, the population size was set to 60, the number of iterations was 10000, and 31 independent experiments were conducted. Finally, the results of 31 times were validated to Friedman mean rank test. Through observation of the data in Table 1, we can realize that although when the value of *γ* is set to 0.5 does not achieve the optimal results on all test functions, it is still a best choice in general. In addition, the results given by the Friedman calibration, *γ* set to 0.5 is also the best choice. Therefore, in this paper, the Cauchy distribution which obeys *X*~*C*(0, 0.5) is used.

**Table 1:** The experimental of HGJO with different *γ*.

Problem		*γ* = 0.25	*γ* = 0.5	*γ* = 0.75	*γ* = 1	*γ* = 1.5
F1	Mean	**300**	**300**	**300**	**300**	**300**
	Std	3.91E-13	**7.190E-14**	1.651E-13	3.226E-13	2.044E-13
F2	Mean	403.94923	**400**	403.47218	404.04741	403.60078
	Std	2.13E+00	**4.896E-10**	1.359E+00	1.636E+00	1.198E+00
F3	Mean	**600.00001**	**600.00001**	**600.00001**	**600.00001**	**600.00001**
	Std	9.11E-06	1.446E-05	**5.991E-06**	1.747E-05	2.060E-05
F4	Mean	809.56445	**804.65384**	809.53234	808.28063	808.89044
	Std	3.52E+00	**5.963E-01**	3.102E+00	2.509E+00	2.436E+00
F5	Mean	**900**	**900**	**900**	**900**	**900**
	Std	4.00E-13	1.164E-12	5.142E-12	1.415E-12	**1.925E-13**
F6	Mean	**1800.1992**	1800.2297	1800.2195	1800.2166	1800.2062
	Std	1.12E-01	**8.529E-02**	1.190E-01	1.087E-01	9.270E-02
F7	Mean	2010.8465	**2002.9588**	2010.7526	2008.7908	2005.678
	Std	9.43E+00	**4.874E+00**	9.548E+00	9.465E+00	7.783E+00
F8	Mean	2201.6878	2201.8592	2202.8334	2201.4274	**2201.1876**
	Std	1.50E+00	1.340E+00	4.659E+00	1.191E+00	**9.500E-01**
F9	Mean	**2529.2844**	**2529.2844**	**2529.2844**	**2529.2844**	**2529.2844**
	Std	**0.00E+00**	**0.000E+00**	**0.000E+00**	**0.000E+00**	**0.000E+00**
F10	Mean	**2500.2164**	2500.2173	2500.2308	2503.8003	2500.2212
	Std	4.38E-02	5.886E-02	**3.934E-02**	1.990E+01	4.141E-02
F11	Mean	**2600**	**2600**	**2600**	**2600**	**2600**
	Std	**5.87E-11**	1.422E-10	2.445E-10	4.723E-10	1.364E-10
F12	Mean	2860.0375	**2859.0842**	2860.0648	2859.8318	2860.2065
	Std	1.21E+00	**4.359E-01**	1.138E+00	1.199E+00	1.103E+00
**Friedman mean rank**		**2.7500**	**2.3333**	**3.5417**	**3.4583**	**2.9167**
**Rank**		**2**	**1**	**5**	**4**	**3**

#### 3.2.2.Dynamic balance strategy

**Fig 2:**
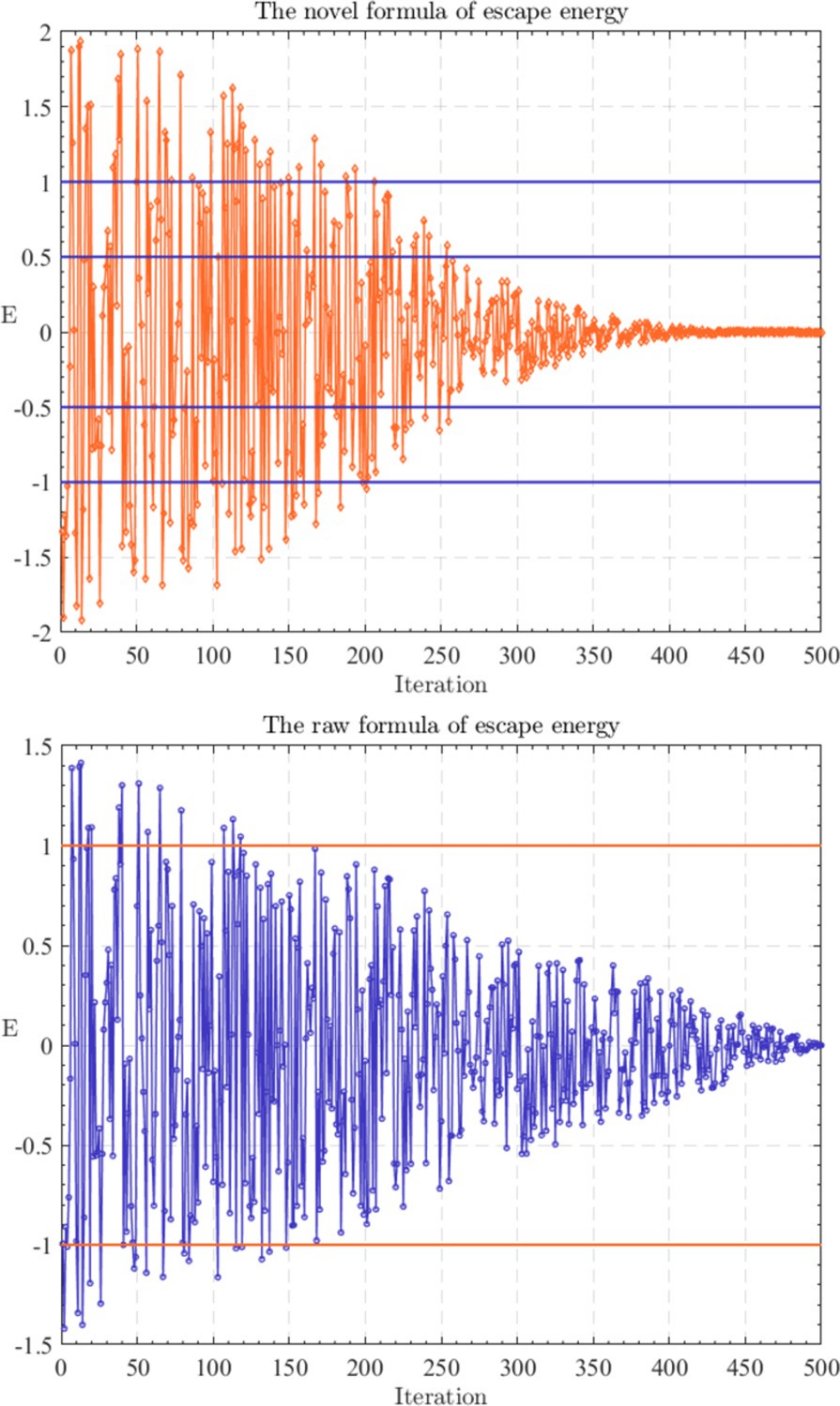
The variation curves of the two escape energies.

The balance of exploration and exploitation essentially determines the performance of an algorithm [59]. It is not hard to see the escape energy ($\vec{E}$) in the GJO determines the exploration and exploitation. However, the calculation of $\vec{E}$ is defined by *E*_1_, which is a value that varies linearly according to the iteration. Thus, GJO might cause exploration and exploitation to be insufficiently balanced during the iteration. In order to overcome this drawback, we propose a novel formula to calculate $\vec{E}$, which is shown in Eq (17). Fig 2 compares the variation curves of the two escape energies.


$$\vec{E}=E_{1}\cdot \vec{E_{0}},\;E_{1}=2\times {\left(1-t/T\right)}^{\pi t/T},\;\vec{E_{0}}=2\cdot \vec{r}-1$$ (17)

All variables have the same implications as those mentioned in the previous section. Therefore, they are not explained here. Note that how the proposed new escape energy will affect the exploration and exploitation of the algorithm is described in detail in Section 3.3.

### 3.3.The new update strategy

Combined with the improvements proposed in the previous two sections, the position update strategy is also modified in this section. In the raw algorithm, exploration and exploitation are divided into two opposite parts, the algorithm will proceed to exploration when the escape energy is greater than 1, and vice versa. However, in this study, we split the iteration into three parts. First, there is the stage where the escape energy is greater than 1. In this stage, all individuals will explore. Then, there is a phase with escape energy greater than 0.5, in which a part of the individuals in the population explores and the rest exploits. Finally, the stage with escape energy less than 0.5, in which all individuals were exploiting. The detailed mathematical model is shown below:

1. In the first stage, when $mean(abs(\vec{E}))>1$, the population will explore. However, their exploration is divided into two parts, part with reference to the current position of the individual, and the other part with reference to the center position of the whole population.

$$\{\begin{array}{c} \begin{array}{c} \vec{X_{1}}=\vec{X_{k}}(t)-\vec{E}\cdot |\vec{X_{m}}(t)-\vec{RC}\cdot \vec{X_{k}}(t)|\\ \vec{X_{2}}=\vec{X_{k}}(t)-\vec{E}\cdot |\vec{X_{fm}}(t)-\vec{RC}\cdot \vec{X_{k}}(t)| \end{array},\;rand>0.5\\ \begin{array}{c} \vec{X_{1}}=\vec{X_{k}}(t)-\vec{E}\cdot |\vec{X_{m}}(t)-\vec{RC}\cdot mean(X)|\\ \vec{X_{2}}=\vec{X_{k}}(t)-\vec{E}\cdot |\vec{X_{fm}}(t)-\vec{RC}\cdot mean(X)| \end{array},otherwise \end{array}$$ (18)

where $\vec{RC}$ means a random vector generated by the Cauchy distribution, which obeys *X*~*C*(0, 0.5). *rand* denotes a random number between 0 and 1. *mean*(*X*) represents the central position of the current population. The rest of the variables have the same meaning as before.

2. In the second stage, when $0.5<mean(abs(\vec{E}))<1$, the population will transition from exploration to exploitation. In this process, a portion of the individuals will maintain the exploration, while the rest will transform to exploitation.

$$\{\begin{array}{c} \begin{array}{c} \vec{X_{1}}=\vec{X_{k}}(t)-\vec{E}\cdot |\vec{X_{m}}(t)-\vec{RC}\cdot \vec{X_{k}}(t)|\\ \vec{X_{2}}=\vec{X_{k}}(t)-\vec{E}\cdot |\vec{X_{fm}}(t)-\vec{RC}\cdot \vec{X_{k}}(t)| \end{array},rand>0.5\\ \begin{array}{c} \vec{X_{1}}=\vec{X_{m}}(t)-\vec{E}\cdot |\vec{RC}\cdot \vec{X_{m}}(t)-\vec{X_{k}}(t)|\\ \vec{X_{2}}=\vec{X_{fm}}(t)-\vec{E}\cdot |\vec{RC}\cdot \vec{X_{fm}}(t)-\vec{X_{k}}(t)| \end{array},otherwise \end{array}$$ (19)

All the variables have the same meaning as before.

3. In the last stage, when $mean(abs(\vec{E}))<0.5$, the population will enter the exploitation stage. In this stage, a part of the individuals will be exploitation depending on themselves, while the rest of the individuals will be exploitation depending on the center position of the population.

$$\{\begin{array}{c} \begin{array}{c} \vec{X_{1}}=\vec{X_{m}}(t)-\vec{E}\cdot |\vec{RL}\cdot \vec{X_{m}}(t)-\vec{X_{k}}(t)|\\ \vec{X_{2}}=\vec{X_{fm}}(t)-\vec{E}\cdot |\vec{RL}\cdot \vec{X_{fm}}(t)-\vec{X_{k}}(t)| \end{array},\;rand>0.5\\ \begin{array}{c} \vec{X_{1}}=\vec{X_{m}}(t)-\vec{E}\cdot |\vec{RL}\cdot \vec{X_{m}}(t)-mean(X)|\\ \vec{X_{2}}=\vec{X_{fm}}(t)-\vec{E}\cdot |\vec{RL}\cdot \vec{X_{fm}}(t)-mean(X)| \end{array},otherwise \end{array}$$ (20)

As well, all variables have the same meaning as mentioned before. Therefore, there is no need to go into too much detail.

### 3.4.The helper mechanism

In general, there are individuals in the golden jackal group which are called “helpers” [49]. These helpers are the previous offspring of the golden jackal. Golden jackal populations are strengthened by helpers. This study focuses on “helpers” to enhance the global searchability of the algorithm with the introduction of “helpers”, which can effectively prevent the algorithm trapped into local optimal.

1. The first part of the “helpers” is to support the growth of the golden jackal pups. The mathematical model of this part is shown below:

$$\begin{array}{l} \vec{X_{k}}(t+1)=\left\{ \begin{array}{c} \vec{X_{k}}(t+1),fitness(\vec{X_{k}}(t+1))<fitness(\vec{X_{offspring}}(t))\\ \vec{X_{offspring}}(t),otherwise \end{array}\right.\\ \vec{X_{offspring}}(t)=\vec{X_{helper1}}(t)+\vec{E}\cdot (\vec{X_{helper2}}(t)-\vec{X_{helper3}}(t)) \end{array}$$ (21)

where $\vec{X_{helper1}},\vec{X_{helper2}}$ and $\vec{X_{helper3}}$ represent three random individuals, respectively. If the obtained offspring has a better fitness than the current updated individual, the individual position is updated to the position of the offspring.

2. The second part of the “helpers” is to take care of the pups while the golden jackal parents are out hunting. When other foragers are present, or something happens which could be harmful to the safety of the pups, the “helpers” will assist the pups in avoiding the danger. This part of the mechanism can be shown by how the algorithm escapes from the local optimal solution, which the mathematical model is as follows.

$$\begin{array}{l} \vec{X_{k}}(t+1)=\left\{ \begin{array}{c} \vec{X_{k}}(t+1),fitness(\vec{X_{k}}(t+1))<fitness(\vec{X_{helper}}(t))\\ \vec{X_{helper}}(t),otherwise \end{array}\right.\\ \vec{X_{helper}}(t)=\vec{X_{i}}(t)+rand\cdot (\vec{X_{rand1}}(t)-\vec{X_{rand2}}(t)) \end{array}$$ (22)

where $\vec{X_{rand1}},\vec{X_{rand2}}$ represent two random individuals, respectively. *rand* denotes a random number between 0 and 1. At this point, the improvement of IGJO is almost complete. The pseudo-code of HGJO is given in Algorithm 2.

**Algorithm 2** Pseudo-code of the HGJO

**Inputs**: The Population Size *N* and the Max iterations *T*.

**Outputs**: The best solution.

1. Initialization the population *X* according Eq (15).

2. **while** (*t*<*T*)

3. Calculating the fitness of the population.

4. $\vec{X_{M}}(t)=best(X(t))$

5. $\vec{X_{FM}}(t)=second\_best(X(t))$

6. foreach (*X*(*t*))

7.  Update the Evasion Energy ($\vec{E}$) according Eq (17).

8.  if ($mean(\left|\vec{E}\right|)>1$)

9.   Update the population according Eqs (12) and (18).

10.  **elseif** ($mean(\left|\vec{E}\right|)>0.5$)

11.   Update the population according Eqs (12) and (19).

12.  **else**

13.   Update the population according Eqs (12) and (20).

14.  **end if**

15.  Calculating, comparing and updating the fitness of offspring and current individual according Eq (21).

16. **end foreach**

17. Global perturbation with helpers according Eq (22).

18. *t* = *t*+1

19. **end while**

20. **return** the best solution ($\vec{X_{M}}$)

### 3.5.Computational complexity

#### 3.5.1. Time complexity

Based on the pseudo-code in Algorithm 2, we could derive the time complexity of HGJO without difficulty. In which, the initial population spends *O*(N*M) time to generated, where *N* denotes the size of the population, *M* represents the dimensions of the decision space. Then, the fitness of the population needs *O*(T*N*O_*f*_) time to calculate, where *T* indicates the maximum iterations, *O*_*f*_ is the cost of object function. In addition, the population needs *O*(T*N*M) time to be updated. Therefore, the total time complexity is *O*(N*(T*(*O*_*f*_+*M*)+*M*)).

#### 3.5.2. Space complexity

Since no additional memory space is used in the computation, the space complexity of HGJO is limited only by the population size. Hence, the space complexity of HGJO is *O*(M*N).

## 4. Experimental results and analysis

In this section, we will evaluate the performance of the proposed algorithm. We will compare HGJO with six existing meta-heuristics on the CEC2022 test suite. These six meta-heuristics include the original GJO algorithm, the first variant of GJO algorithm called IGJO which uses OBL for improvement, two recently proposed widely used meta-heuristics, the RUN algorithm and the Archimedes Optimization Algorithm (AOA) [60], and the two most classical and stable algorithms, DE and PSO. Additionally, to further ensure that combining the OBL operator with the GJO algorithm is the most feasible option, we also included the OBL operator in the DE and PSO algorithms for comparison in the experiments. There are 12 different test functions in the CEC2022 test suite, which can cover a majority of the real-world problems. Therefore, the contents of this section enable us to make a preliminary understanding of the performance for HGJO. The details of the CEC2022 test suite are given in Table 2 and the runtime environment is also shown in Table 3. All algorithms are iterated with a population size of 60 and a maximum iteration of 1000. Furthermore, in consideration of the suggestion by Arcuri et al. [61], all algorithm parameters are kept at their default values which are derived from their raw papers to ensure they are in a relatively optimal state, and these parameters are provided in Table 4. Moreover, the source code of the CEC2022 test set is available at: https://github.com/P-N-Suganthan/2022-SO-BO.

**Table 2:** The benchmark functions of CEC2022.

Problem No.	Problem name	Dim	Range	F_min
F1	Zakharov Function	10	[–100, 100]	300
F2	Rosenbrock’s Function	10	[–100, 100]	400
F3	Schaffer’s F7	10	[–100, 100]	600
F4	Rastrigin’s Function	10	[–100, 100]	800
F5	Levy Function	10	[–100, 100]	900
F6	Hybrid Function 1	10	[–100, 100]	1800
F7	Hybrid Function 2	10	[–100, 100]	2000
F8	Hybrid Function 3	10	[–100, 100]	2200
F9	Composition Function 1	10	[–100, 100]	2300
F10	Composition Function 2	10	[–100, 100]	2400
F11	Composition Function 3	10	[–100, 100]	2600
F12	Composition Function 4	10	[–100, 100]	2700

**Table 3:** Runtime environment.

Configurations	
**Hardware**	
CPU	Intel(R) Core(TM) i9-10980HK CPU @ 2.40GHz
GPU	NVIDIA GeForce RTX 3080 Laptop GPU
RAM	64.0 GB
**Software**	
OS	Microsoft Windows [Version 10.0.19043.1826]
Interpreter	IntelliJ IDEA 2021.3.2 (Ultimate Edition)
Language	MATLAB R2021a (9.10.0.1602886)

**Table 4:** The parameters setting of all algorithms.

Algorithm	Parameters
HGJO	*C*_1_ = 2
GJO	*C*_1_ = 1.5
IGJO	*C*_1_ = 1.5
RUN	*a* = 20, *b* = 12
AOA	*C*_1_ = 2, *C*_2_ = 6, *C*_3_ = 2, *C*_4_ = 0.5
DE(OBL)	*P*_*Cr*_ = 0.8, *F* = 0.85
PSO(OBL)	*C*_1_ = 1.49445, *C*_2_ = 1.49445

### 4.1.Statistical results on CEC2022

**Fig 3:**
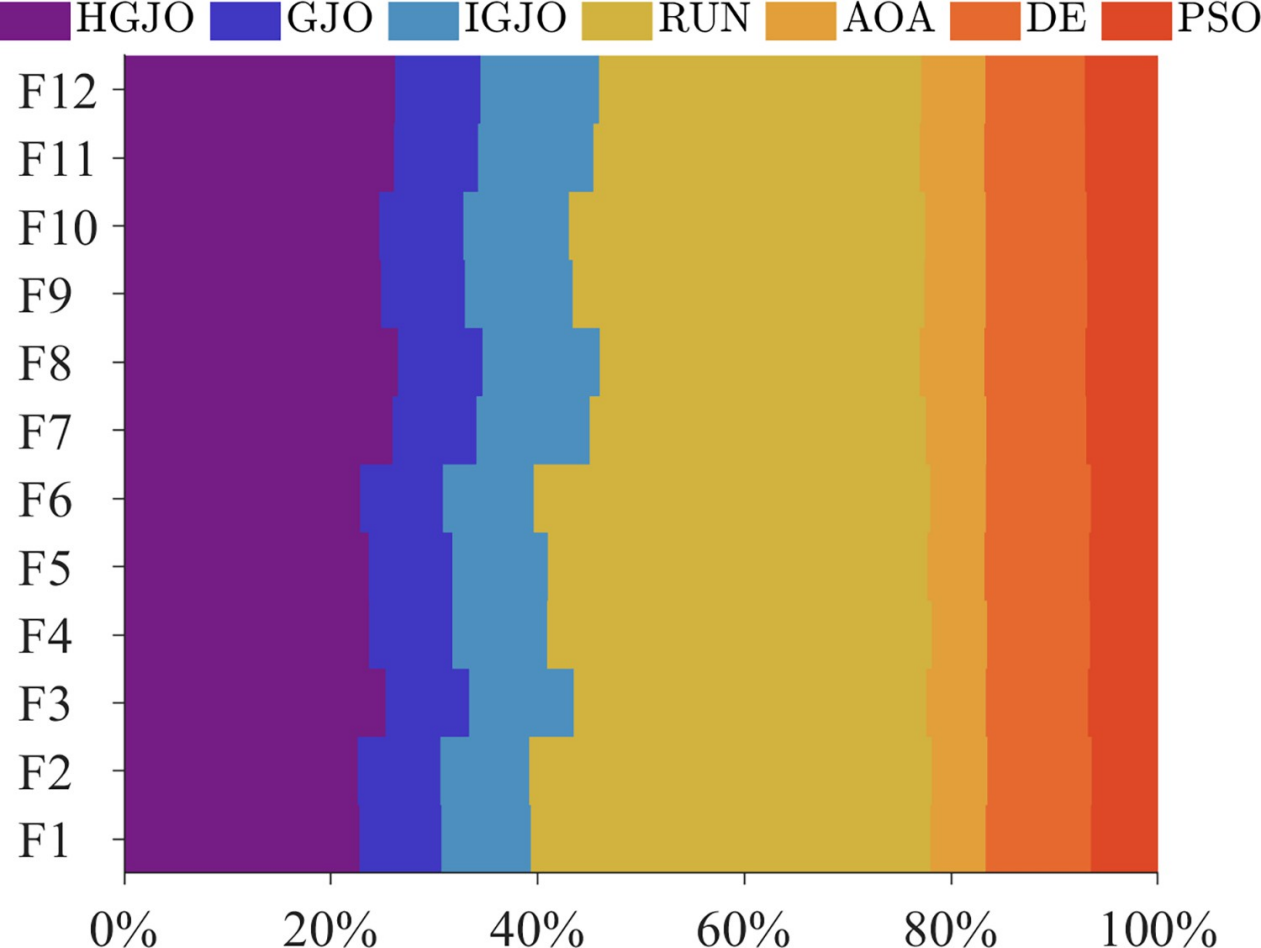
The average time slots for all algorithms.

As mentioned above, the CEC2022 test suite is used to measure the performance of each algorithm, which includes both quantitative and qualitative metrics. The quantitative metrics include the mean, median, and standard deviation obtained by all algorithms. Qualitative metrics are illustrated by convergence curves, which reflect the evolution of the optimal solution throughout the iterations of the algorithm. To ensure the fairness of the experiments, all algorithms were run 31 times independently on the CEC2022 benchmark test function. Table 5 provides the average time spent by all algorithms over the 31 runs, and lists the median, mean, and standard deviation of the best values obtained by all algorithms, the best results (minimum value) was highlighted in bold. The Friedman mean rank [62] was used to determine the overall rank of each algorithm. According to the data in Table 5, we can see that the proposed method is optimal for most problems in terms of mean and median, except for F9 where AOA obtains the most accurate value. Similarly, the proposed algorithm also achieves satisfactory results for standard deviation in most problems. Therefore, we can consider that the proposed algorithm has excellent solving performance in CEC2022. However, in terms of running time, the AOA algorithm is the shortest in all problems, while the HGJO algorithm has some shortcomings compared to it. Considering that the introduction of OBL and Cauchy operator will affect the efficiency of the algorithm to some extent, Fig 3 further analyzes the running time of all algorithms in CEC2022. Fig 3 is the average time slot of all algorithms running in CEC2022, which can reflect the percentage of time each algorithm consumes when processing the same problem. The horizontal coordinate is the percentage of time consumed, and the vertical coordinate is the test function. Through Fig 3, we can clearly observe that compared with the original GJO, the time complexity of HGJO has increased significantly, but it still has some advantages compared with RUN. In addition, through the observation of 12 test functions, we can see that the running time of HGJO has not fluctuated significantly. Although OBL and Cauchy operator will increase certain time costs, the proposed algorithm shows the most satisfactory results in terms of overall performance. In addition, the Friedman mean rank is also used to comprehensively rank the algorithms, and the top three algorithms are HGJO, DE(OBL), and AOA, respectively.

**Table 5:** The results of CEC2022 for all algorithm.

Problem		HGJO	GJO	IGJO	RUN	AOA	DE	PSO
F1	Mean	**300.000**	682.748	699.541	**300.000**	300.001	1215.047	1220.769
	Median	**300.000**	439.932	456.433	**300.000**	**300.000**	1153.934	1217.032
	Std	**1.228E-13**	7.593E+02	7.395E+02	6.385E-05	1.627E-03	3.405E+02	6.384E+01
	Time	1.072	0.362	0.395	1.973	**0.268**	0.524	0.329
F2	Mean	**403.472**	425.274	422.150	403.587	405.245	405.504	434.336
	Median	403.987	411.882	409.887	**400.013**	400.257	404.124	427.549
	Std	**1.359E+00**	2.210E+01	1.993E+01	4.363E+00	1.139E+01	2.219E+00	1.857E+01
	Time	1.149	0.362	0.389	1.976	**0.271**	0.533	0.327
F3	Mean	**600.000**	605.511	605.925	611.779	600.245	600.064	615.090
	Median	**600.000**	604.463	605.740	608.887	600.097	600.061	612.536
	Std	**1.419E-05**	4.645E+00	4.768E+00	6.793E+00	2.906E-01	2.314E-02	5.356E+00
	Time	1.460	0.457	0.572	2.241	**0.371**	0.639	0.437
F4	Mean	**808.762**	819.404	822.509	823.847	817.139	861.592	845.546
	Median	**808.955**	818.711	821.114	823.879	817.909	861.478	845.007
	Std	2.819E+00	5.913E+00	7.876E+00	6.765E+00	5.615E+00	**1.967E+00**	2.538E+00
	Time	1.246	0.394	0.448	2.041	**0.303**	0.568	0.363
F5	Mean	**900.000**	959.189	967.710	977.192	908.414	900.041	977.753
	Median	**900.000**	951.961	942.420	968.285	900.454	900.014	977.414
	Std	**3.771E-13**	6.716E+01	8.875E+01	4.827E+01	3.302E+01	6.664E-02	6.523E+00
	Time	1.275	0.399	0.457	2.066	**0.307**	0.542	0.369
F6	Mean	**1800.226**	7151.939	7110.417	2921.693	2532.632	1814.253	4354616.518
	Median	**1800.211**	8154.988	8182.686	2533.530	2171.087	1814.067	4264276.228
	Std	**9.605E-02**	2.193E+03	1.907E+03	1.012E+03	9.174E+02	1.567E+00	7.624E+05
	Time	1.202	0.371	0.408	1.988	**0.280**	0.540	0.343
F7	Mean	**2006.963**	2032.196	2037.376	2037.731	2021.994	2019.993	2064.246
	Median	**2001.372**	2031.836	2034.197	2037.757	2021.643	2021.894	2062.244
	Std	8.907E+00	1.136E+01	1.121E+01	9.988E+00	9.576E+00	6.431E+00	**5.565E+00**
	Time	1.674	0.510	0.689	2.138	**0.398**	0.654	0.457
F8	Mean	**2201.458**	2224.956	2225.259	2223.042	2219.205	2210.591	2247.440
	Median	**2201.065**	2225.255	2225.280	2223.540	2221.317	2207.073	2235.872
	Std	**1.409E+00**	4.580E+00	2.978E+00	1.564E+00	6.726E+00	8.650E+00	3.649E+01
	Time	1.667	0.548	0.761	2.302	**0.482**	0.747	0.528
F9	Mean	2529.284	2549.393	2551.107	2529.285	**2493.923**	2529.284	2545.972
	Median	2529.284	2531.291	2541.380	2529.284	**2493.923**	2529.284	2536.194
	Std	**0.000E+00**	3.477E+01	2.645E+01	2.205E-04	8.775E-05	**0.000E+00**	3.849E+01
	Time	1.436	0.470	0.604	2.127	**0.375**	0.608	0.426
F10	Mean	**2500.230**	2570.959	2543.721	2530.174	2551.838	2500.876	2579.107
	Median	**2500.225**	2614.641	2500.606	2500.631	2500.519	2500.874	2648.197
	Std	**4.586E-02**	6.120E+01	5.936E+01	5.094E+01	5.767E+01	6.567E-02	7.631E+01
	Time	1.350	0.460	0.579	2.026	**0.352**	0.580	0.413
F11	Mean	**2600.000**	2800.106	2725.587	2622.627	2644.051	2600.001	2808.208
	Median	**2600.000**	2735.136	2731.824	2600.018	2600.001	2600.001	2761.214
	Std	**1.947E-10**	1.735E+02	9.684E+01	7.946E+01	1.051E+02	4.481E-04	1.260E+02
	Time	1.711	0.531	0.729	2.175	**0.431**	0.695	0.513
F12	Mean	**2859.847**	2865.104	2865.137	2863.978	2878.827	2861.082	2866.534
	Median	**2859.369**	2864.532	2864.505	2863.883	2867.589	2861.405	2866.482
	Std	1.183E+00	3.054E+00	4.156E+00	1.668E+00	1.965E+01	9.283E-01	**7.997E-01**
	Time	1.699	0.554	0.768	2.202	**0.443**	0.699	0.513
**Friedman mean rank**		**1.1667**	**4.8333**	**5.0833**	**3.9583**	**3.3333**	**2.9583**	**6.6667**
**Rank**		**1**	**5**	**6**	**4**	**3**	**2**	**7**

Table 6 uses the Wilcoxon rank-sum test [63] to further evaluate the running results of each algorithm. The Wilcoxon rank-sum test is used to verify whether there is a significant difference between algorithms. When the p-value is less than 0.05, it can be considered that there is a significant difference between algorithms. On the contrary, it means that the performance of the proposed algorithm is similar to that of the compared algorithm. To better represent the analysis of values, we use the symbols "**++**" and "**—**" to indicate the cases where the p-value is less than 0.05 and greater than 0.05, respectively. From Table 6, we can clearly see that the proposed algorithm has significant differences compared with the original GJO, IGJO, RUN, and PSO(OBL). Combined with the data in Table 5, we can consider that it has significant improvements compared with the above algorithms. For AOA and DE(OBL), only similar performance was shown in F2 and F9, respectively. It is worth noting that we can see from the data in Table 5 that AOA achieved the best result in F9, which is also reflected in the Wilcoxon rank-sum test (significant difference between HGJO and AOA). Overall, according to the results of the Wilcoxon rank-sum test, we can consider that the proposed HGJO algorithm has higher performance on the CEC2022 test set.

**Table 6:** The Wilcoxon signed-rank test for CEC2022.

Problem	HGJO vs. GJO	HGJO vs. IGJO	HGJO vs. RUN	HGJO vs. AOA	HGJO vs. DE	HGJO vs. PSO
F1	6.018E-07 **++**	6.018E-07 **++**	6.018E-07 **++**	6.018E-07 **++**	6.018E-07 **++**	6.018E-07 **++**
F2	6.018E-07 **++**	1.588E-06 **++**	4.457E-02 **++**	**3.001E-01 —**	6.352E-06 **++**	6.018E-07 **++**
F3	6.018E-07 **++**	6.018E-07 **++**	6.018E-07 **++**	6.018E-07 **++**	6.018E-07 **++**	6.018E-07 **++**
F4	7.329E-07 **++**	6.642E-07 **++**	6.018E-07 **++**	1.588E-06 **++**	6.018E-07 **++**	6.018E-07 **++**
F5	6.018E-07 **++**	6.018E-07 **++**	6.018E-07 **++**	6.018E-07 **++**	6.018E-07 **++**	6.018E-07 **++**
F6	6.018E-07 **++**	6.018E-07 **++**	6.018E-07 **++**	6.018E-07 **++**	6.018E-07 **++**	6.018E-07 **++**
F7	1.192E-06 **++**	6.018E-07 **++**	6.018E-07 **++**	6.252E-05 **++**	2.308E-02 **++**	6.018E-07 **++**
F8	6.018E-07 **++**	6.018E-07 **++**	6.642E-07 **++**	1.312E-06 **++**	2.906E-03 **++**	6.018E-07 **++**
F9	6.018E-07 **++**	6.018E-07 **++**	6.018E-07 **++**	6.018E-07 **++**	**NaN —**	6.018E-07 **++**
F10	6.018E-07 **++**	6.018E-07 **++**	6.642E-07 **++**	6.018E-07 **++**	6.642E-07 **++**	6.018E-07 **++**
F11	6.018E-07 **++**	6.018E-07 **++**	6.018E-07 **++**	6.018E-07 **++**	6.018E-07 **++**	6.018E-07 **++**
F12	6.642E-07 **++**	6.018E-07 **++**	1.192E-06 **++**	5.057E-04 **++**	1.451E-02 **++**	6.018E-07 **++**

Fig 3 further shows the average time slots achieved by each algorithm on the IEEE CEC2022 benchmark test function. The figure displays the percentage of time consumed by each algorithm in processing the same test function. The horizontal coordinate is the percentage of time consumed, and the vertical coordinate is the test function. Through this figure, we can observe that the time consumption of the HGJO algorithm increases to some extent, but there is no significant fluctuation for the overall. Therefore, this phenomenon verifies that the performance of the proposed algorithm is not limited to a particular problem. This makes the algorithm more extensible and can be more widely ap-plied to other optimization problems.

### 4.2.Convergence behavior analysis

**Fig 4:**
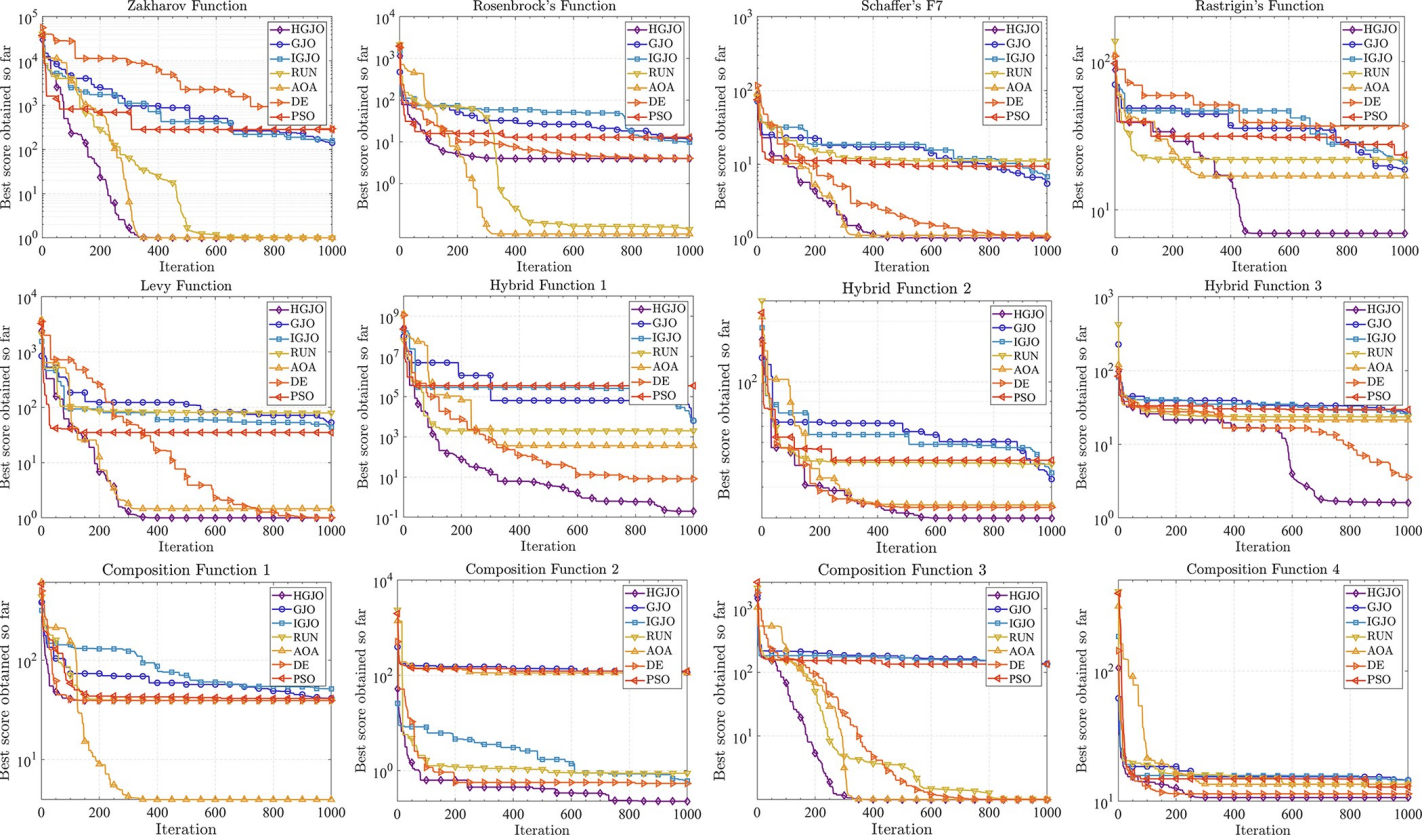
The convergence curves for all algorithms on CEC2022 benchmark functions.

This subsection further presents the results of the convergence analysis of HGJO compared to other meta-heuristics. Fig 4 shows the convergence curves of all the algorithms for the CEC2022 benchmark test function. It is worth noting that all the curves in Fig 4 are calculated as the difference between the optimal values of their benchmark functions. It does not alter the exact meaning of the convergence curves but additionally enhances the observability of the images on the logarithmic axis. In short, the value closer to 0 indicates that the optimal solution obtained by the algorithm is closer to the true optimal solution. With these convergence plots, we can see that the proposed algorithm reaches a stable point in all benchmark test functions, which can further indicate that the proposed algorithm is convergent. In addition, for most test functions, HGJO achieved the optimal solution with the least number of iterations, except for F2 and F9 where it was surpassed by RUN and AOA. Therefore, by analyzing the convergence of the proposed algorithm and other competing algorithms, the superiority of HGJO is further validated, making it possible for HGJO to replace existing algorithms to solve complex problems.

### 4.3.Boxplot behavior analysis

**Fig 5:**
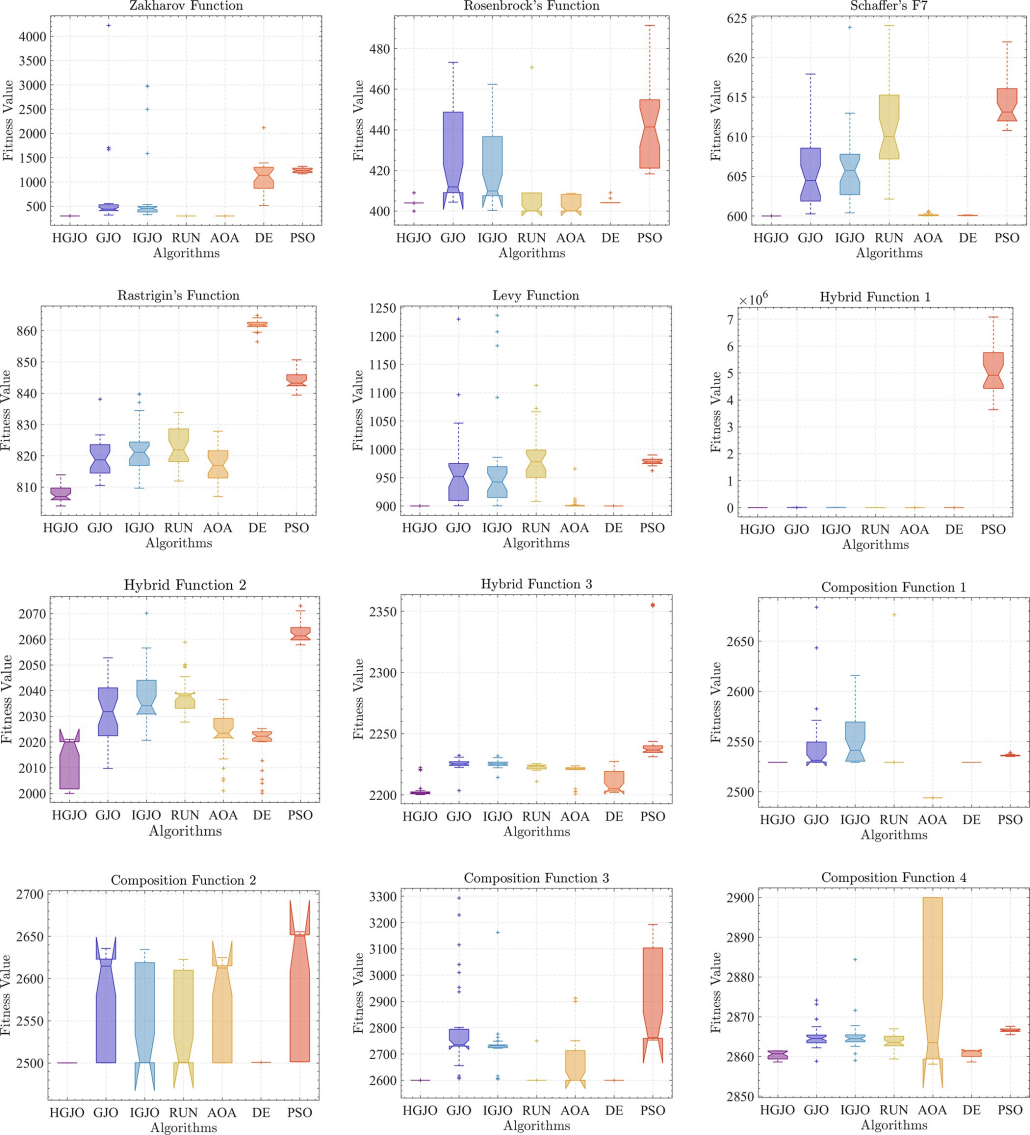
The box plots for all algorithms on CEC2022 benchmark functions.

Due to the many local optima of the CEC2022 benchmark test functions, solving these problems can easily fall into local optima. In order to analyze the algorithmic results more intuitively, in this section we use boxplots to analyze HGJO and other metaheuristics. Fig 5 shows boxplots for all algorithms on the CEC2022 benchmark test function. The boxplots provide a visual representation of the distribution characteristics of the data, with the maximum and minimum values of the data corresponding to the highest and lowest points of the image, respectively. Therefore, the narrower the image reflects, the more stable the data. For most benchmark test functions, the proposed method has the narrowest and lowest boxplots. In fact, HGJO outperforms other metaheuristic algorithms in most test functions, except for F9. Combining the performance analysis of HGJO mentioned above, we can reasonably speculate that the proposed algorithm has the ability to solve complex engineering problems in the real world, providing a new candidate solution for scientific researchers to choose from. In the next section, we will use HGJO to handle the optical aerial image segmentation problem.

## 5. The optical aerial image segmentation

In this section, HGJO is applied to a real-world scenario of multi-level thresholding segmentation. The HGJO and all the comparison algorithms in the previous section are used to perform threshold segmentation on the optical aerial image. The objective function used is the Otsu method introduced in Section 2.1. Finally, all algorithms are evaluated by *PSNR*, *SSIM*, and *FSIM* obtained after processing.

### 5.1.Dataset and runtime environment

**Fig 6:**
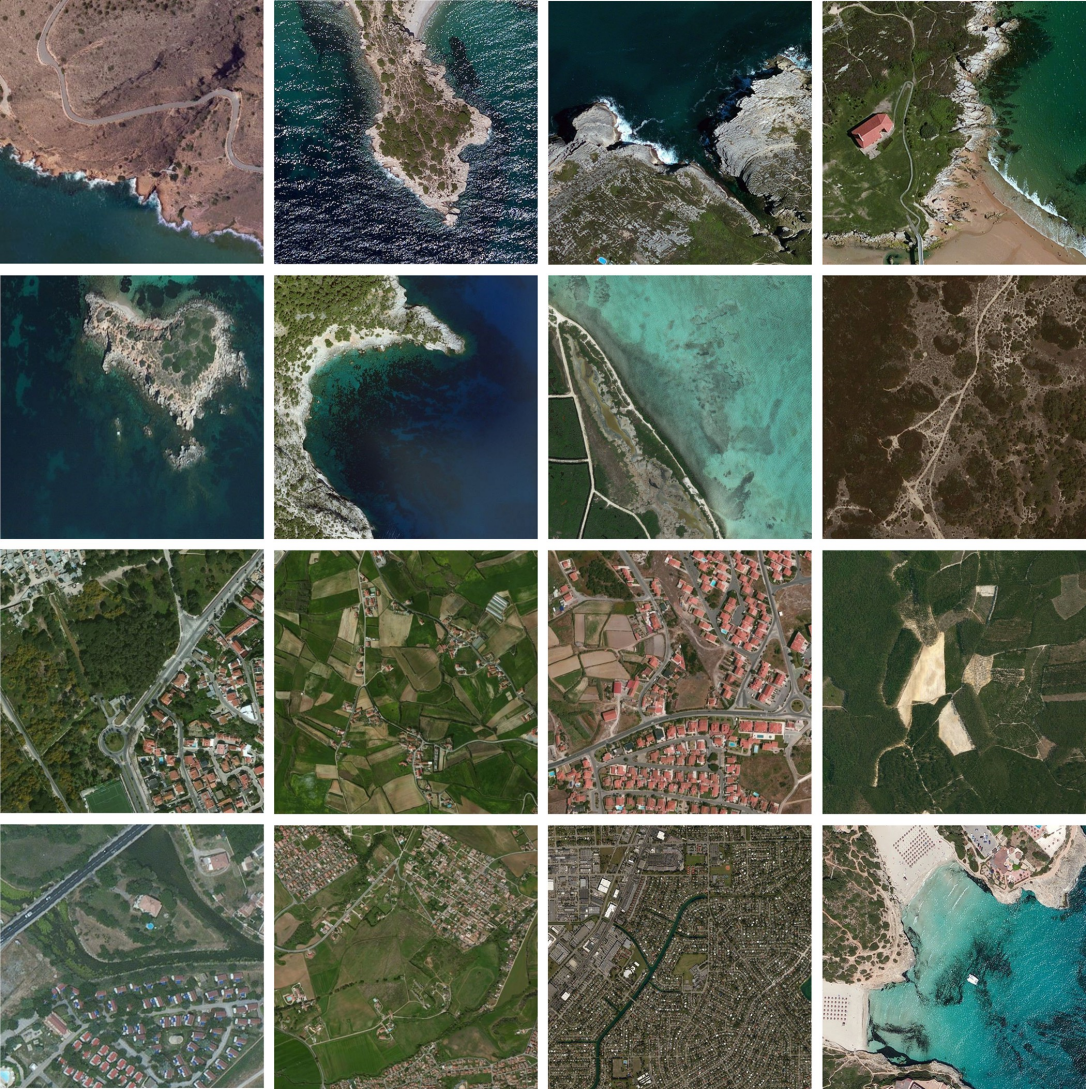
The selected images for segmentation.

**Fig 7:**
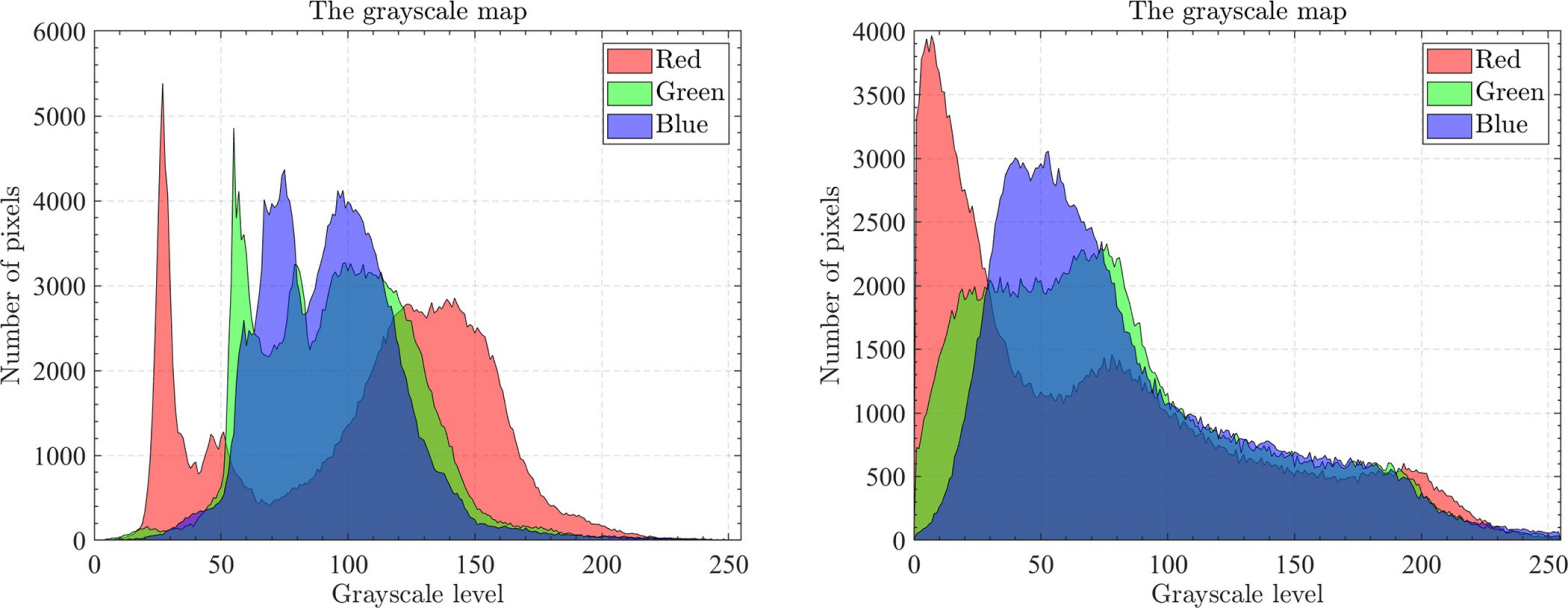
The RGB histogram of C0080 and C0088.

**Fig 8:**
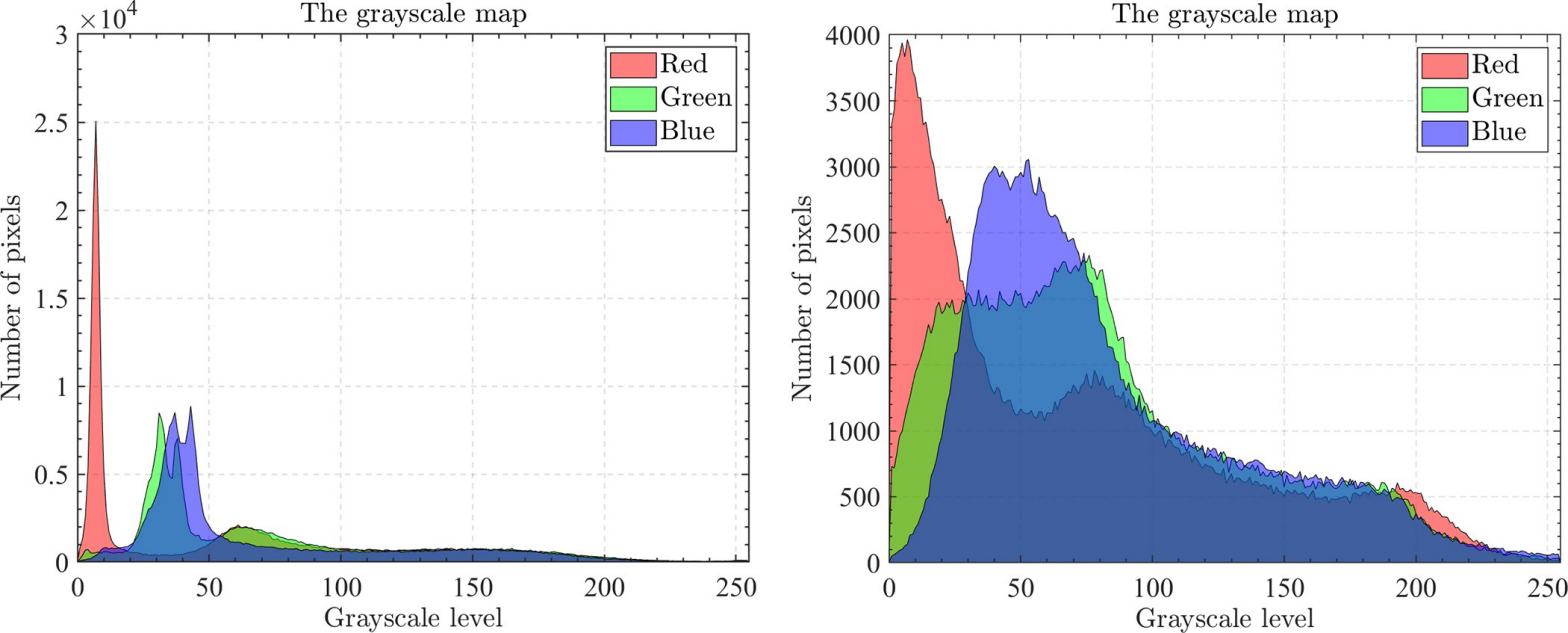
The RGB histogram of C0132 and C0135.

**Fig 9:**
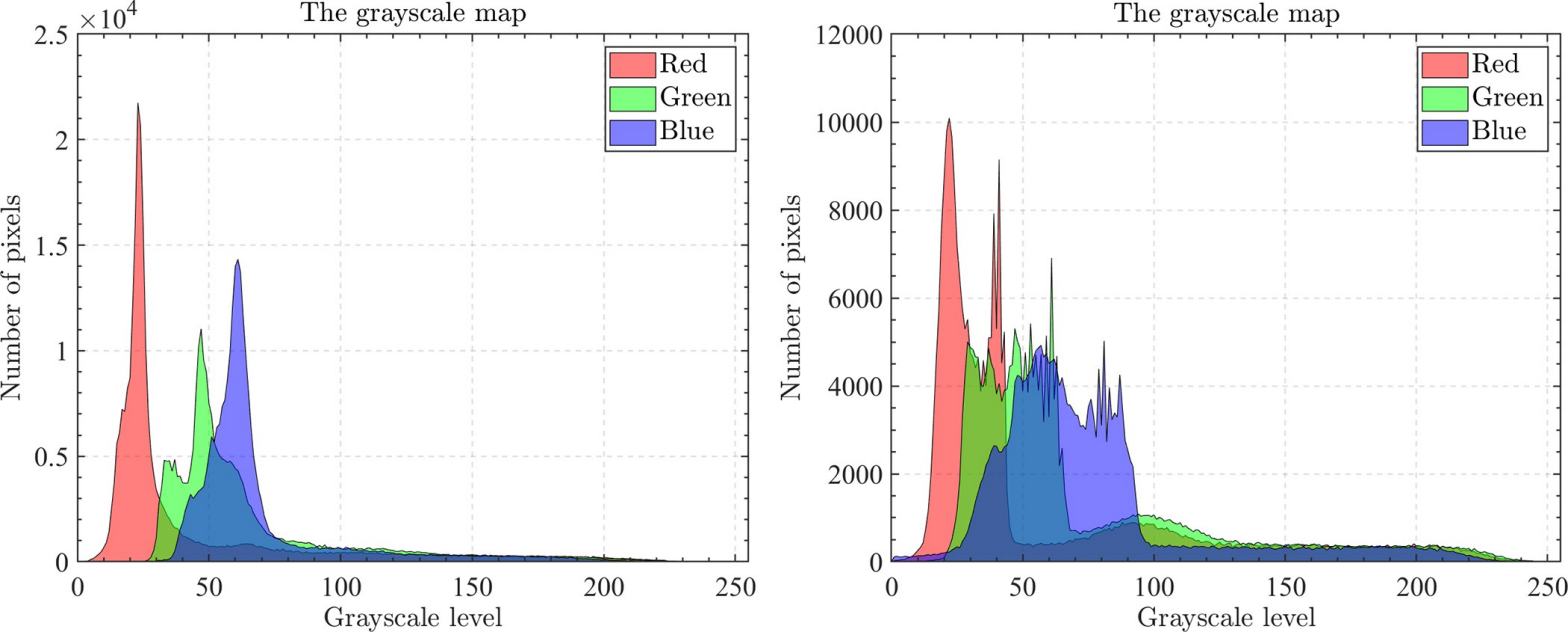
The RGB histogram of C0180 and C0536.

**Fig 10:**
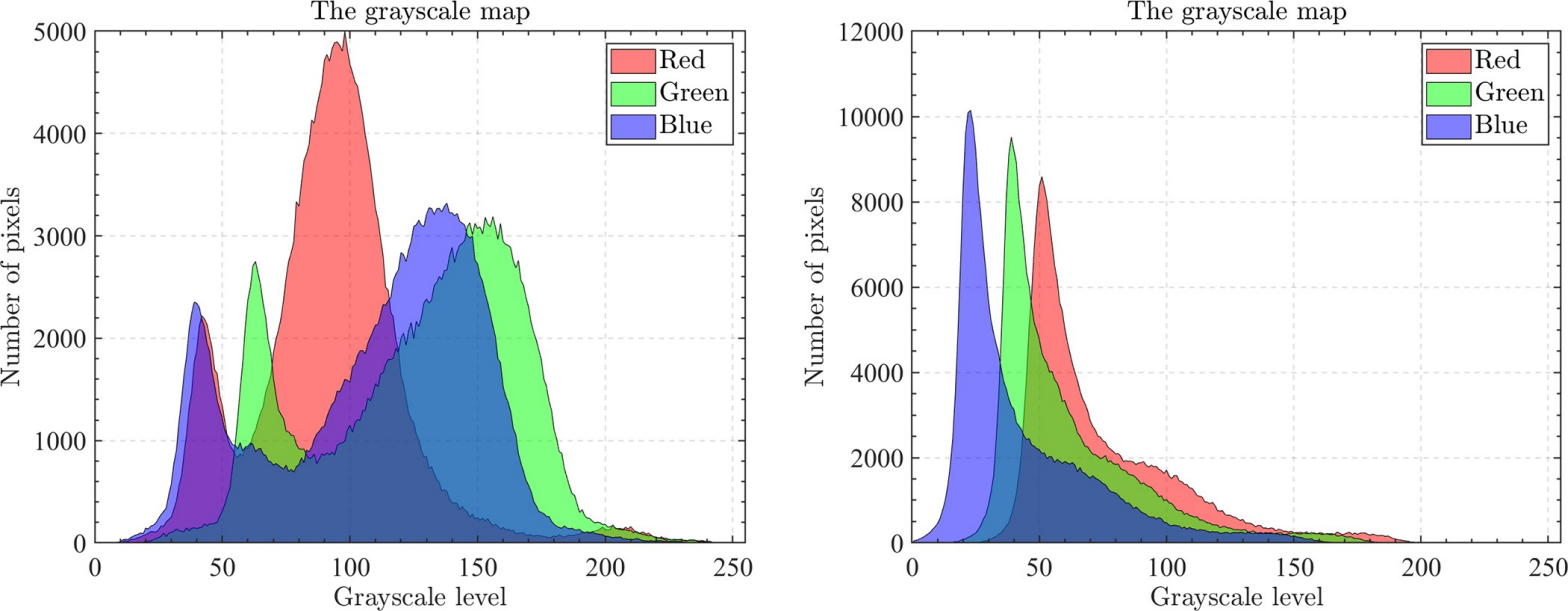
The RGB histogram of C1088 and L0032.

**Fig 11:**
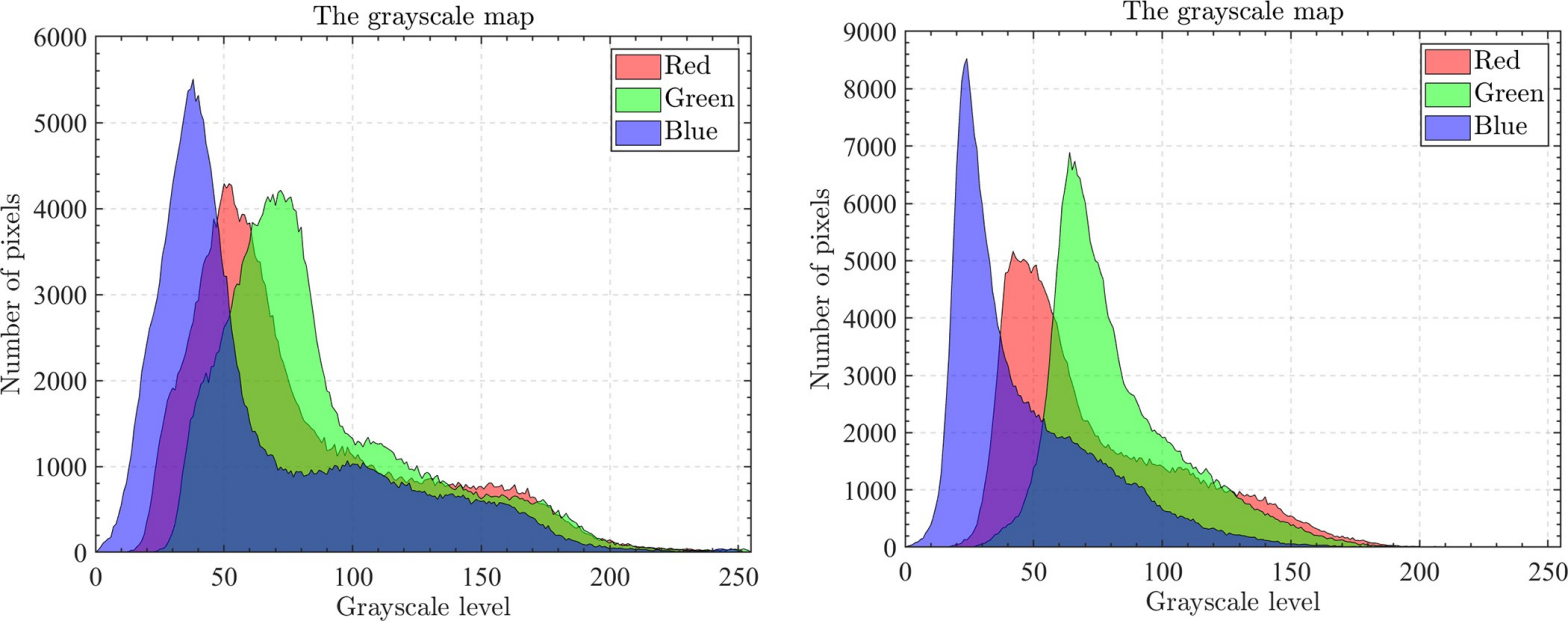
The RGB histogram of L0064 and L0135.

**Fig 12:**
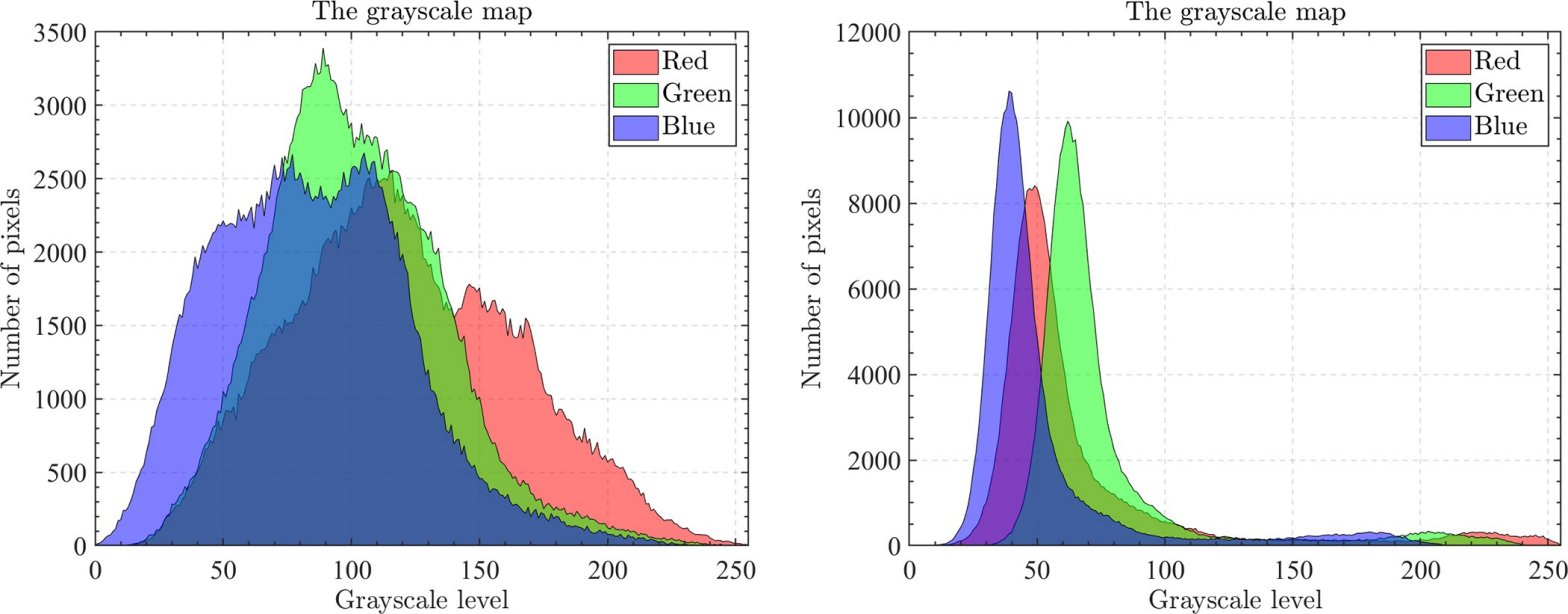
The RGB histogram of L0158 and L0226.

**Fig 13:**
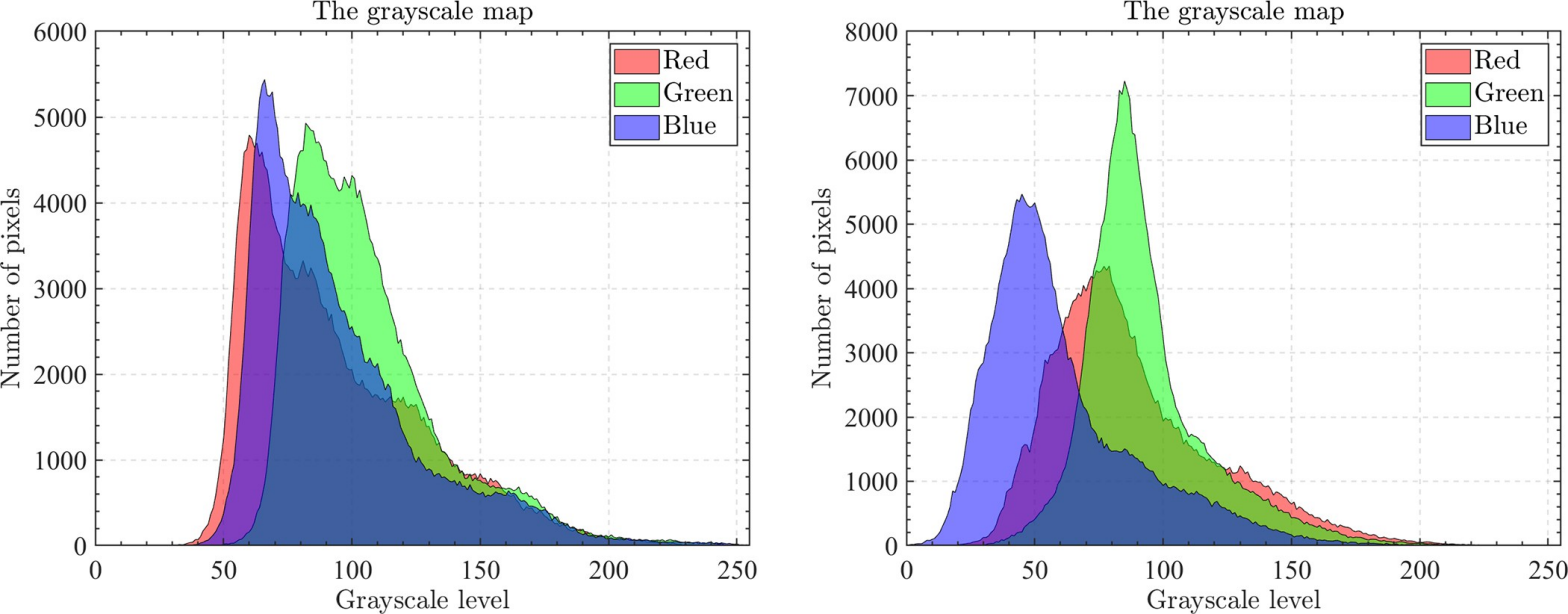
The RGB histogram of L0699 and L0879.

**Fig 14:**
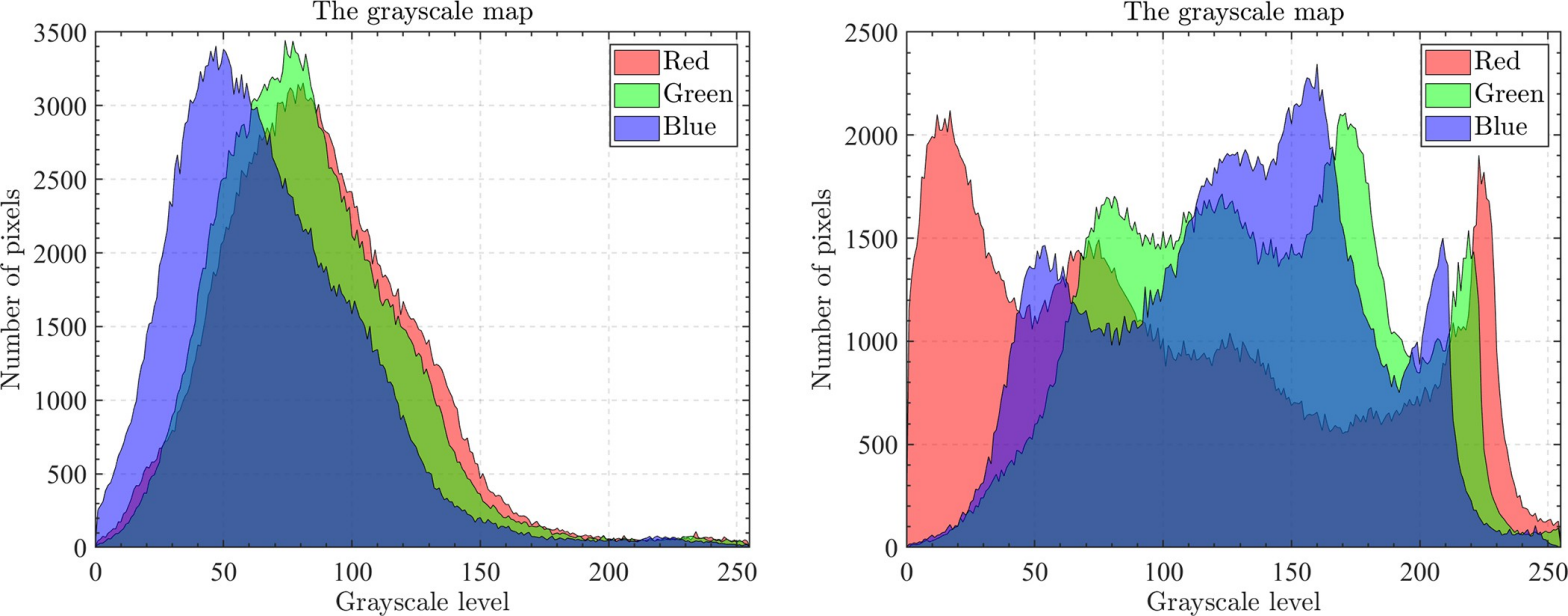
The RGB histogram of L1074 and X0017.

The optical aerial image used for image segmentation experiments are from the MASATI dataset [64], where each image has a size of 512×512. In this study, 16 images are selected for segmentation, which were named C0080, C0088, C0132, C0135, C0180, C0536, C1088, L0032, L0064, L0135, L0158, L0226, L0699, L0879, L1074, and X0017, as shown in Fig 6. All of these images are characterized by their own features which cover the vast majority of optical aerial image types. Furthermore, Figs 7–14 show the RGB histogram of all the selected images. The threshold values of each image are set to 8, 16, 24, 32, respectively. To ensure the fairness of the experiments, the population size was fixed to 60, the maximum number of iterations was set to 1000 and all the algorithms were run independently 31 times with the same configured environment.

### 5.2.The evaluation metrics

As mentioned above, in this study *PSNR*, *SSIM*, and *FSIM* will be used as the performance metrics for image segmentation. This subsection will describe the effects of these three metrics in detail.

First is *PSNR*, peak signal-to-noise ratio, which is used to measure the difference between two images, which has a minimum value of 0 to represent the maximum difference. The mathematical formula of *PSNR* can be described as follows:

$$PSNR=20\;\log_{10}\frac{255}{RMSE},RMSE=\sqrt{\frac{\sum_{i=1}^{M}\sum_{j=1}^{N}{\left(Iin(i,j)-Iout(i,j)\right)}^{2}}{M\times N}}$$ (23)

where *RMES* is the root mean square error, *Iin* and *Iout* are the original image and the segmented image, which have the size of *M*×*N*, respectively.

Next is *SSIM*, Structural Similarity, which is used to evaluate the similarity between two images. The value of *SSIM* is between 0 and 1, and if two images are completely identical, then the *SSIM* is equal to 1. Its mathematical model can be described as follows:

$$SSIM(Iin,Iout)=\frac{\left(2\mu_{Iin}\mu_{Iout}+c_{1})(2\delta_{Iin,Iout}+c_{2}\right)}{\left(\mu_{Iin}^{2}+\mu_{Iout}^{2}+c_{1})(\delta_{Iin}^{2}+\delta_{Iout}^{2}+c_{2}\right)}$$ (24)

where *μ*_*Iin*_ and *μ*_*Iout*_ are the mean intensities of the original image and segmented image, respectively, *σ*_*Iin*_ and *σ*_*Iout*_ are the standard deviations of the original image and segmented image, respectively, *σ*_*Iin*_,_*Iout*_ is the covariance of the original and segmented images, and *C*_1_ and *C*_2_ are two constants.

Finally, is *FSIM*, Feature Similarity, which is calculated by Phase Consistency (*PC*) and Gradient Magnitude (*GM*). It reflects the difference in features between the two images. The *FSIM* has the same value as the *SSIM*, which is also between 0 and 1, and the closer to 1 indicates that the feature information of the two images is more similar. It can be described as follows:

$$FSIM=\frac{\sum_{x\in \Omega }S_{L}(x)PC_{m}(x)}{\sum_{x\in \Omega }PC_{m}(x)}$$ (25)

$$S_{L}(x)={\left[S_{PC}(x)\right]}^{\alpha }{\left[S_{G}(x)\right]}^{\beta }$$ (26)

$$S_{PC}(x)=\frac{2PC_{Iin}(x)PC_{Iout}(x)+T_{1}}{PC_{Iin}^{2}(x)+PC_{Iout}^{2}(x)+T_{1}}$$ (27)

$$S_{G}(x)=\frac{2G_{Iin}(x)G_{Iout}(x)+T_{2}}{G_{Iin}^{2}(x)+G_{Iout}^{2}(x)+T_{2}}$$ (28)

where *PC*_*Iin*_ and *PC*_*Iout*_ are the *PC* of the original image and segmented image, respectively, and *T*_1_ is a positive constant used to increase the stability of *S*_*PC*_. *G*_*Iin*_ and *G*_*Iout*_ represent the gradients of the original and segmented images, respectively, while *T*_2_ is also a positive constant that controls the range of *GM*. *α* and *β* are two constants, respectively. In short, for all three metrics, *PSNR*, *SSIM*, and *FSIM*, the bigger the better.

### 5.3.Experimental results and analysis

In this section, the experimental outcomes of multilevel thresholding segmentation are analyzed by combining images and tables. To verify the adaptivity of HGJO in handling the segmentation task, the selected optical aerial images were segmented at threshold levels of 8, 16, 24, and 32, respectively. The algorithms involved in the segmentation comparison are consistent with the previous section, and the experimental results are evaluated by three metrics: *PSNR*, *SSIM*, and *FSIM*. In addition, the comprehensive performance of all algorithms is ranked using the Friedman mean rank test, and the Wilcoxon rank-sum test is used to analyze the fitness of all algorithms on the Otsu method.

Tables 7–9 show the mean and std value of the *PSNR*, *SSIM*, and *FSIM*, respectively. It is noteworthy that the maximum mean and minimum std are highlighted in the tables. In addition, it should be mentioned that the segmentation results of the same algorithm on different images may behave differently. This is because each image corresponds to a different problem being processed. When compared to other algorithms, HGJO performs exceptionally well at the specified threshold level. Additionally, as the threshold level increases, this performance does not degrade.

**Table 7:** The *PSNR* results for all algorithms.

Image Name	Level	HGJO		GJO		IGJO		RUN		AOA		DE		PSO	
		Mean	STD	Mean	STD	Mean	STD	Mean	STD	Mean	STD	Mean	STD	Mean	STD
C0080	8	**24.49**	4.05E-02	22.23	7.44E-01	23.29	4.29E-01	22.48	6.72E-01	23.35	2.77E-01	24.38	**1.37E-02**	18.67	1.23E+00
	16	**29.64**	**1.23E-01**	27.32	9.44E-01	27.37	9.60E-01	28.06	6.36E-01	28.32	5.83E-01	27.62	3.88E-01	23.61	9.81E-01
	24	**34.82**	5.66E-01	30.57	8.25E-01	29.67	1.13E+00	31.79	**5.29E-01**	31.60	6.71E-01	29.30	1.08E+00	26.08	1.35E+00
	32	**37.86**	**2.54E-01**	31.39	1.12E+00	31.66	9.20E-01	33.13	7.09E-01	34.18	6.69E-01	31.09	1.16E+00	29.66	1.01E+00
C0088	8	**24.43**	**3.82E-03**	23.80	1.87E-01	24.14	9.64E-02	24.19	5.86E-02	24.32	3.60E-02	24.42	6.67E-03	19.90	9.88E-01
	16	**30.31**	**3.29E-02**	27.69	5.86E-01	28.11	5.34E-01	28.63	3.54E-01	29.67	1.49E-01	28.87	1.31E-01	25.37	7.67E-01
	24	**33.88**	**7.34E-02**	30.29	7.56E-01	30.99	4.90E-01	31.65	3.20E-01	32.76	1.47E-01	31.43	2.36E-01	26.86	8.28E-01
	32	**36.45**	**1.20E-01**	32.80	6.64E-01	32.77	5.70E-01	33.59	5.92E-01	35.21	1.44E-01	33.11	3.22E-01	29.08	8.26E-01
C0132	8	**25.06**	9.41E-03	24.04	3.70E-01	24.00	3.24E-01	24.50	1.32E-01	24.47	1.84E-01	**25.06**	**2.55E-03**	21.80	7.87E-01
	16	**30.21**	**1.12E-01**	28.45	4.60E-01	28.44	5.82E-01	28.83	3.68E-01	28.56	3.12E-01	27.15	7.10E-01	24.53	1.03E+00
	24	**33.67**	**1.32E-01**	30.53	8.49E-01	30.76	5.64E-01	31.96	4.37E-01	32.31	2.78E-01	30.18	6.07E-01	27.45	8.25E-01
	32	**36.54**	**2.06E-01**	31.11	1.11E+00	33.01	7.12E-01	34.66	4.48E-01	34.42	3.38E-01	31.44	7.07E-01	29.51	9.41E-01
C0135	8	**24.88**	1.49E-02	22.72	6.23E-01	23.80	2.50E-01	24.62	7.07E-02	24.66	4.91E-02	24.85	**1.29E-02**	20.93	6.83E-01
	16	**30.86**	**2.71E-02**	27.30	7.52E-01	28.88	4.79E-01	29.04	3.22E-01	29.84	1.88E-01	28.69	3.60E-01	22.41	1.42E+00
	24	**34.36**	**5.83E-02**	31.40	4.87E-01	30.84	7.32E-01	31.60	4.42E-01	32.33	3.44E-01	31.46	2.91E-01	28.01	8.52E-01
	32	**36.99**	**1.55E-01**	33.25	7.34E-01	32.73	8.07E-01	33.97	5.09E-01	34.77	2.39E-01	33.18	3.62E-01	28.91	1.12E+00
C0180	8	**25.93**	**7.28E-15**	24.13	7.54E-01	24.14	4.32E-01	25.79	4.54E-02	24.48	6.67E-01	25.92	1.60E-02	19.93	1.34E+00
	16	**31.50**	**1.29E-01**	28.27	8.29E-01	29.09	7.22E-01	29.28	4.50E-01	29.58	6.41E-01	28.28	5.83E-01	26.59	1.08E+00
	24	**35.56**	**1.79E-01**	30.85	9.47E-01	31.25	9.23E-01	31.90	6.97E-01	31.40	8.09E-01	31.20	7.19E-01	26.83	1.45E+00
	32	**38.02**	**3.85E-01**	33.10	7.69E-01	34.51	5.80E-01	34.81	7.18E-01	34.78	6.03E-01	32.67	8.31E-01	29.27	1.46E+00
C0536	8	**23.96**	1.20E-02	23.10	5.06E-01	23.15	3.92E-01	**23.96**	1.72E-01	23.92	1.30E-01	23.93	**7.28E-15**	19.21	9.90E-01
	16	**30.47**	**2.06E-01**	27.36	8.19E-01	28.59	5.59E-01	29.66	3.74E-01	28.43	4.67E-01	28.44	5.46E-01	25.25	1.10E+00
	24	**34.82**	**2.39E-01**	30.63	8.74E-01	31.74	6.40E-01	32.40	3.95E-01	32.08	3.16E-01	30.74	6.31E-01	26.26	1.42E+00
	32	**37.09**	**2.12E-01**	32.63	1.08E+00	33.44	6.58E-01	34.26	5.18E-01	34.63	3.94E-01	33.00	5.55E-01	30.15	1.10E+00
C1088	8	**22.72**	3.61E-02	21.41	6.58E-01	20.96	6.48E-01	21.90	5.84E-01	22.55	1.57E-01	22.64	**1.70E-02**	14.25	2.08E+00
	16	**30.20**	**1.52E-01**	25.01	1.14E+00	26.89	8.42E-01	27.74	5.48E-01	28.49	6.76E-01	26.10	9.36E-01	20.20	1.72E+00
	24	**34.42**	**2.71E-01**	28.28	1.25E+00	28.86	8.17E-01	29.57	8.20E-01	31.94	7.32E-01	30.19	8.93E-01	25.24	1.76E+00
	32	**37.08**	**2.96E-01**	29.26	1.13E+00	30.97	1.08E+00	30.14	1.23E+00	33.39	8.25E-01	32.09	7.79E-01	25.89	1.83E+00
L0032	8	**19.41**	8.40E-02	18.27	3.89E-01	18.27	3.90E-01	18.27	1.36E+00	18.77	3.27E-01	19.13	**5.91E-04**	17.15	1.24E+00
	16	**23.11**	**2.20E-01**	18.27	3.27E+00	19.61	3.33E+00	20.23	3.08E+00	20.01	2.88E+00	21.07	9.06E-01	19.21	1.87E+00
	24	26.59	**5.89E-01**	22.93	2.89E+00	21.06	3.70E+00	**28.12**	1.30E+00	22.29	2.55E+00	20.99	1.83E+00	19.33	3.11E+00
	32	**33.67**	**1.51E+00**	24.71	2.36E+00	26.27	2.36E+00	23.23	3.22E+00	24.63	2.52E+00	21.87	2.73E+00	21.09	3.25E+00
L0064	8	**22.97**	9.47E-02	21.97	4.48E-01	22.70	2.81E-01	21.11	7.57E-01	22.70	2.40E-01	22.70	**8.62E-02**	20.32	7.46E-01
	16	29.35	**2.97E-01**	26.34	1.38E+00	28.05	9.55E-01	**29.90**	3.12E-01	27.30	1.18E+00	25.09	9.36E-01	22.62	1.72E+00
	24	**33.15**	**3.16E-01**	27.95	1.45E+00	30.39	8.34E-01	32.42	5.99E-01	31.42	8.71E-01	28.40	9.43E-01	23.92	1.75E+00
	32	**37.55**	6.83E-01	32.91	8.16E-01	32.90	1.06E+00	34.96	**6.14E-01**	33.69	6.63E-01	29.35	1.70E+00	24.30	2.22E+00
L0135	8	**22.65**	**2.40E-03**	21.18	5.20E-01	22.15	8.36E-01	21.20	9.25E-01	22.64	2.73E-01	22.64	2.51E-03	18.91	1.23E+00
	16	26.98	**2.31E-01**	24.73	1.78E+00	23.84	2.53E+00	**27.72**	1.25E+00	25.12	2.11E+00	24.58	1.10E+00	22.73	1.42E+00
	24	**34.26**	1.30E+00	27.25	2.22E+00	27.33	2.12E+00	32.20	**1.07E+00**	27.91	2.58E+00	25.72	1.54E+00	24.53	2.32E+00
	32	**37.40**	2.29E+00	31.09	**1.32E+00**	28.63	2.04E+00	31.15	1.81E+00	28.48	2.80E+00	26.62	1.69E+00	26.11	2.46E+00
L0158	8	**22.18**	3.73E-02	20.09	8.82E-01	20.95	4.25E-01	22.15	7.18E-01	21.80	3.12E-01	22.01	**7.28E-15**	16.66	1.40E+00
	16	28.77	**3.08E-01**	25.99	1.28E+00	25.58	1.38E+00	28.95	5.23E-01	**28.96**	5.72E-01	25.42	9.80E-01	21.43	2.13E+00
	24	**33.98**	5.43E-01	29.91	9.75E-01	28.55	1.47E+00	32.27	**4.67E-01**	32.12	6.11E-01	25.62	1.27E+00	23.21	2.32E+00
	32	**36.91**	**6.09E-01**	32.29	8.77E-01	30.86	1.50E+00	31.23	1.23E+00	33.77	8.02E-01	29.66	1.42E+00	25.87	1.86E+00
L0226	8	**21.42**	**7.28E-15**	19.79	9.06E-01	21.40	2.31E-01	21.29	5.09E-01	21.02	2.70E-01	**21.42**	1.58E-03	19.04	1.48E+00
	16	**29.47**	**4.40E-01**	26.65	1.48E+00	26.62	1.75E+00	28.15	1.14E+00	25.90	1.91E+00	24.03	1.62E+00	21.29	2.26E+00
	24	**36.22**	6.15E-01	32.38	8.56E-01	32.12	9.04E-01	34.04	**4.84E-01**	32.57	9.25E-01	25.81	2.38E+00	25.16	2.00E+00
	32	**39.28**	**2.34E-01**	33.20	9.84E-01	34.61	8.55E-01	36.43	4.99E-01	35.06	6.74E-01	28.01	2.30E+00	25.39	2.28E+00
L0699	8	**21.47**	**3.64E-15**	18.85	1.04E+00	20.49	5.91E-01	20.50	1.39E+00	20.50	8.32E-01	**21.47**	**3.64E-15**	17.97	1.52E+00
	16	**30.10**	**5.00E-01**	24.33	2.06E+00	27.08	1.34E+00	25.85	1.87E+00	27.55	1.56E+00	24.52	1.57E+00	20.41	2.70E+00
	24	**36.47**	**6.57E-01**	28.99	1.53E+00	29.70	1.47E+00	27.37	1.95E+00	32.20	9.90E-01	28.58	1.94E+00	23.38	3.08E+00
	32	**39.63**	**3.95E-01**	31.96	1.50E+00	31.48	1.66E+00	31.51	1.21E+00	35.91	6.84E-01	29.68	2.19E+00	24.74	2.34E+00
L0879	8	**21.24**	2.73E-04	20.50	5.17E-01	19.65	9.02E-01	20.50	1.29E+00	**21.24**	1.96E-01	**21.24**	**3.64E-15**	19.04	1.04E+00
	16	27.92	**4.45E-01**	25.16	2.11E+00	24.97	2.05E+00	**29.20**	9.69E-01	24.41	2.17E+00	23.62	1.07E+00	21.55	1.50E+00
	24	**33.62**	**8.29E-01**	29.70	1.29E+00	24.72	2.66E+00	31.98	1.05E+00	26.94	2.52E+00	25.51	1.89E+00	20.90	2.84E+00
	32	**38.05**	1.48E+00	29.03	1.84E+00	30.59	1.70E+00	30.69	1.98E+00	33.78	**1.39E+00**	26.49	2.54E+00	24.02	2.54E+00
L1074	8	24.33	8.48E-03	23.13	6.21E-01	23.10	3.77E-01	**24.49**	2.97E-01	24.30	2.08E-01	24.30	**7.28E-15**	20.66	1.03E+00
	16	**31.32**	**1.25E-01**	28.94	5.60E-01	29.48	4.96E-01	30.08	3.87E-01	29.82	4.15E-01	28.31	5.99E-01	25.21	9.50E-01
	24	**35.34**	**1.26E-01**	31.97	5.45E-01	32.37	6.48E-01	32.90	4.09E-01	33.08	2.61E-01	31.26	4.99E-01	25.63	1.33E+00
	32	**38.08**	**1.89E-01**	33.39	6.92E-01	34.05	8.71E-01	35.33	4.20E-01	35.10	3.77E-01	32.96	5.24E-01	29.60	8.36E-01
X0017	8	**23.45**	**8.22E-03**	22.19	4.50E-01	22.10	4.04E-01	22.72	1.84E-01	23.25	5.07E-02	23.39	1.47E-02	20.65	6.03E-01
	16	**29.26**	**7.51E-02**	26.58	7.20E-01	27.56	4.75E-01	27.48	3.74E-01	28.68	1.21E-01	27.58	2.35E-01	24.42	9.23E-01
	24	**32.99**	**6.53E-02**	30.39	4.81E-01	29.71	7.04E-01	30.36	4.55E-01	32.18	1.29E-01	30.40	2.57E-01	27.84	5.01E-01
	32	**35.60**	**9.65E-02**	32.26	6.18E-01	32.25	7.39E-01	32.36	5.06E-01	34.54	1.36E-01	32.14	3.50E-01	28.82	7.64E-01
**Friedman mean rank**		**1.1328**		**4.9844**		**4.5781**		**3.0156**		**2.8828**		**4.4219**		**6.98441**	
**Rank**		**1**		**6**		**5**		**3**		**2**		**4**		**7**	

**Table 8:** The *SSIM* results for all algorithms.

Image Name	Level	HGJO		GJO		IGJO		RUN		AOA		DE		PSO	
		Mean	STD	Mean	STD	Mean	STD	Mean	STD	Mean	STD	Mean	STD	Mean	STD
C0080	8	**0.892**	8.99E-04	0.833	1.74E-02	0.868	7.74E-03	0.844	1.39E-02	0.869	5.55E-03	0.890	**3.69E-04**	0.660	4.64E-02
	16	**0.957**	**1.16E-03**	0.937	9.46E-03	0.940	9.50E-03	0.945	6.07E-03	0.945	5.63E-03	0.932	5.13E-03	0.840	2.12E-02
	24	**0.987**	3.13E-03	0.966	4.53E-03	0.963	6.18E-03	0.977	**2.34E-03**	0.974	3.20E-03	0.951	8.67E-03	0.900	1.83E-02
	32	**0.993**	**5.42E-04**	0.973	4.29E-03	0.970	4.15E-03	0.983	1.95E-03	0.985	1.84E-03	0.963	6.76E-03	0.948	8.88E-03
C0088	8	0.938	4.63E-04	0.933	1.56E-03	0.937	7.18E-04	**0.940**	6.52E-04	**0.940**	8.79E-04	0.937	**2.69E-04**	0.841	2.42E-02
	16	**0.983**	**1.40E-04**	0.970	2.84E-03	0.974	2.33E-03	0.978	1.41E-03	0.980	8.15E-04	0.976	1.17E-03	0.951	6.26E-03
	24	**0.993**	**1.33E-04**	0.984	1.82E-03	0.986	1.25E-03	0.989	6.29E-04	0.990	4.02E-04	0.985	1.03E-03	0.961	5.25E-03
	32	**0.996**	**1.14E-04**	0.991	1.11E-03	0.991	7.98E-04	0.993	6.94E-04	0.994	2.35E-04	0.989	8.64E-04	0.974	3.34E-03
C0132	8	**0.859**	**2.48E-04**	0.835	1.06E-02	0.809	1.59E-02	0.838	5.96E-03	0.837	6.73E-03	**0.859**	6.78E-04	0.615	6.47E-02
	16	0.940	**1.50E-03**	0.917	1.31E-02	0.937	1.45E-02	**0.944**	3.81E-03	0.911	8.40E-03	0.872	1.77E-02	0.795	3.49E-02
	24	**0.977**	**1.68E-03**	0.951	8.95E-03	0.945	8.01E-03	0.974	2.32E-03	0.965	3.12E-03	0.938	9.27E-03	0.859	2.28E-02
	32	**0.989**	**1.13E-03**	0.939	1.05E-02	0.980	5.62E-03	0.986	1.57E-03	0.978	2.33E-03	0.941	8.56E-03	0.927	1.57E-02
C0135	8	0.907	7.95E-04	0.858	1.58E-02	0.882	5.95E-03	**0.914**	2.17E-03	0.910	1.88E-03	0.905	**7.59E-04**	0.759	3.13E-02
	16	**0.974**	**3.54E-04**	0.946	6.34E-03	0.960	4.39E-03	0.968	1.96E-03	0.964	2.18E-03	0.951	4.17E-03	0.775	3.83E-02
	24	**0.989**	**2.38E-04**	0.979	2.56E-03	0.977	3.05E-03	0.981	1.45E-03	0.980	1.58E-03	0.976	2.09E-03	0.939	9.22E-03
	32	**0.993**	**3.01E-04**	0.985	1.74E-03	0.984	2.36E-03	0.989	8.99E-04	0.988	7.17E-04	0.981	1.69E-03	0.948	9.13E-03
C0180	8	0.888	**1.14E-16**	0.845	2.11E-02	0.843	1.32E-02	**0.890**	1.75E-03	0.849	2.03E-02	0.888	1.32E-04	0.689	5.44E-02
	16	**0.962**	**1.93E-03**	0.928	1.05E-02	0.939	8.61E-03	0.945	5.41E-03	0.939	8.48E-03	0.908	1.18E-02	0.885	2.21E-02
	24	**0.986**	**8.68E-04**	0.957	6.91E-03	0.958	6.82E-03	0.970	3.44E-03	0.956	6.58E-03	0.949	7.61E-03	0.900	2.12E-02
	32	**0.992**	**1.11E-03**	0.977	4.08E-03	0.980	2.45E-03	0.984	2.33E-03	0.980	2.55E-03	0.960	6.16E-03	0.926	1.60E-02
C0536	8	**0.832**	5.78E-04	0.812	1.58E-02	0.814	1.36E-02	**0.832**	8.51E-03	0.830	6.65E-03	0.831	**3.41E-16**	0.626	5.13E-02
	16	**0.959**	**4.78E-03**	0.932	1.09E-02	0.925	9.92E-03	0.951	5.21E-03	0.925	9.26E-03	0.923	1.14E-02	0.862	2.94E-02
	24	**0.987**	**1.46E-03**	0.963	6.30E-03	0.970	4.19E-03	0.979	2.26E-03	0.969	3.01E-03	0.951	7.51E-03	0.868	2.90E-02
	32	**0.992**	**6.71E-04**	0.974	5.01E-03	0.984	2.64E-03	0.986	1.56E-03	0.984	1.69E-03	0.968	5.25E-03	0.961	1.08E-02
C1088	8	**0.847**	1.02E-03	0.812	1.97E-02	0.795	2.03E-02	0.824	1.71E-02	0.842	4.92E-03	0.845	**4.82E-04**	0.240	1.59E-01
	16	**0.965**	**2.46E-03**	0.898	1.87E-02	0.926	1.08E-02	0.954	4.77E-03	0.941	1.15E-02	0.907	1.40E-02	0.655	5.95E-02
	24	**0.988**	**1.26E-03**	0.938	9.89E-03	0.960	5.27E-03	0.968	4.17E-03	0.983	2.66E-03	0.952	8.91E-03	0.886	2.28E-02
	32	**0.993**	**4.01E-04**	0.966	5.92E-03	0.977	4.26E-03	0.974	3.72E-03	0.986	1.80E-03	0.969	4.01E-03	0.884	2.23E-02
L0032	8	**0.582**	6.35E-03	0.496	2.95E-02	0.496	2.92E-02	0.496	7.65E-02	0.534	2.39E-02	0.561	**1.41E-05**	0.533	9.14E-02
	16	**0.778**	**8.88E-03**	0.496	1.24E-01	0.595	1.08E-01	0.639	7.73E-02	0.620	8.59E-02	0.684	3.84E-02	0.576	9.30E-02
	24	0.869	**1.39E-02**	0.773	5.81E-02	0.686	9.87E-02	**0.916**	1.50E-02	0.742	5.78E-02	0.521	8.35E-02	0.579	1.19E-01
	32	**0.963**	**2.15E-02**	0.829	3.49E-02	0.865	3.35E-02	0.797	5.47E-02	0.880	3.06E-02	0.799	4.73E-02	0.702	8.43E-02
L0064	8	**0.812**	3.68E-03	0.781	1.39E-02	0.802	1.10E-02	0.751	2.72E-02	0.802	9.50E-03	0.802	**3.33E-03**	0.702	3.21E-02
	16	0.941	5.31E-03	0.894	2.56E-02	0.919	1.84E-02	**0.964**	**4.50E-03**	0.907	2.09E-02	0.853	2.07E-02	0.813	4.32E-02
	24	0.972	3.10E-03	0.921	1.53E-02	0.947	8.76E-03	**0.984**	**1.84E-03**	0.959	8.91E-03	0.916	1.35E-02	0.819	3.57E-02
	32	**0.992**	3.66E-03	0.980	2.92E-03	0.972	6.22E-03	0.988	**1.33E-03**	0.978	3.10E-03	0.927	1.84E-02	0.827	3.80E-02
L0135	8	**0.793**	**1.37E-04**	0.740	1.85E-02	0.778	3.00E-02	0.740	3.41E-02	0.793	1.05E-02	**0.793**	1.48E-04	0.582	5.97E-02
	16	0.904	**4.98E-03**	0.857	3.03E-02	0.834	4.23E-02	**0.923**	1.39E-02	0.863	3.45E-02	0.847	2.41E-02	0.790	3.71E-02
	24	**0.982**	1.37E-02	0.910	2.34E-02	0.909	2.27E-02	0.979	**3.20E-03**	0.918	2.40E-02	0.875	2.30E-02	0.846	3.80E-02
	32	**0.990**	1.30E-02	0.961	6.98E-03	0.929	1.42E-02	0.971	**5.55E-03**	0.924	2.05E-02	0.890	2.18E-02	0.884	3.26E-02
L0158	8	0.865	1.03E-03	0.809	2.35E-02	0.834	1.07E-02	**0.868**	1.58E-02	0.855	7.76E-03	0.861	**4.55E-16**	0.670	4.68E-02
	16	0.960	**3.19E-03**	0.931	1.34E-02	0.924	1.58E-02	**0.970**	3.64E-03	0.963	5.19E-03	0.918	1.31E-02	0.825	3.69E-02
	24	**0.988**	2.69E-03	0.968	5.32E-03	0.957	8.87E-03	0.987	**1.13E-03**	0.983	2.22E-03	0.915	1.35E-02	0.880	2.94E-02
	32	**0.994**	**1.82E-03**	0.983	2.24E-03	0.972	5.72E-03	0.979	3.31E-03	0.987	2.18E-03	0.962	8.50E-03	0.921	1.78E-02
L0226	8	0.680	**2.28E-16**	0.614	4.48E-02	0.679	1.04E-02	**0.684**	2.44E-02	0.661	1.23E-02	0.680	7.45E-05	0.561	6.85E-02
	16	0.910	**1.07E-02**	0.866	3.50E-02	0.840	4.26E-02	**0.912**	2.30E-02	0.822	4.62E-02	0.760	4.64E-02	0.664	8.24E-02
	24	**0.987**	5.91E-03	0.951	1.01E-02	0.973	9.77E-03	0.984	**1.89E-03**	0.953	1.11E-02	0.809	4.83E-02	0.798	4.95E-02
	32	**0.994**	**1.03E-03**	0.953	8.19E-03	0.984	3.13E-03	0.990	1.09E-03	0.971	4.35E-03	0.859	3.39E-02	0.791	4.62E-02
L0699	8	**0.760**	**2.28E-16**	0.670	3.33E-02	0.727	1.83E-02	0.728	3.77E-02	0.728	2.37E-02	**0.760**	**2.28E-16**	0.601	5.31E-02
	16	**0.934**	**7.78E-03**	0.832	3.56E-02	0.889	2.27E-02	0.865	2.85E-02	0.896	2.45E-02	0.833	2.84E-02	0.662	7.43E-02
	24	**0.988**	**4.64E-03**	0.922	1.65E-02	0.925	1.43E-02	0.897	2.04E-02	0.953	8.70E-03	0.905	2.32E-02	0.808	7.42E-02
	32	**0.994**	**1.22E-03**	0.960	6.83E-03	0.960	1.03E-02	0.963	6.47E-03	0.984	2.45E-03	0.920	1.91E-02	0.788	4.26E-02
L0879	8	**0.789**	9.89E-06	0.765	1.71E-02	0.733	3.13E-02	0.765	4.03E-02	**0.789**	6.74E-03	**0.789**	**2.28E-16**	0.705	4.08E-02
	16	0.936	7.58E-03	0.891	2.92E-02	0.884	2.79E-02	**0.963**	**6.92E-03**	0.873	2.91E-02	0.853	2.23E-02	0.792	4.52E-02
	24	0.981	5.95E-03	0.959	8.38E-03	0.877	2.76E-02	**0.983**	**2.66E-03**	0.919	1.91E-02	0.893	2.59E-02	0.760	5.82E-02
	32	**0.994**	5.51E-03	0.945	1.09E-02	0.961	7.91E-03	0.969	6.45E-03	0.981	**4.26E-03**	0.908	2.60E-02	0.857	3.41E-02
L1074	8	0.929	6.34E-05	0.909	1.08E-02	0.909	7.15E-03	**0.932**	6.41E-03	0.929	4.24E-03	0.929	**0.00E+00**	0.833	2.63E-02
	16	**0.986**	**8.00E-04**	0.976	2.79E-03	0.976	2.51E-03	0.983	1.33E-03	0.978	2.20E-03	0.965	5.00E-03	0.944	1.01E-02
	24	**0.995**	**2.31E-04**	0.989	9.63E-04	0.989	1.60E-03	0.991	6.29E-04	0.990	5.82E-04	0.981	2.24E-03	0.923	1.32E-02
	32	**0.997**	**1.56E-04**	0.992	9.63E-04	0.993	1.08E-03	0.995	3.91E-04	0.994	4.66E-04	0.986	1.69E-03	0.969	4.78E-03
X0017	8	**0.921**	**2.38E-04**	0.898	8.22E-03	0.896	7.49E-03	0.908	3.50E-03	0.918	1.06E-03	**0.921**	3.04E-04	0.869	1.15E-02
	16	**0.978**	**3.25E-04**	0.959	4.91E-03	0.966	2.80E-03	0.966	2.54E-03	0.975	6.07E-04	0.966	1.63E-03	0.924	1.02E-02
	24	**0.989**	3.28E-04	0.981	1.50E-03	0.979	2.33E-03	0.981	1.50E-03	0.988	**3.13E-04**	0.980	1.48E-03	0.964	3.61E-03
	32	**0.994**	**1.62E-04**	0.988	1.50E-03	0.987	1.67E-03	0.988	1.12E-03	0.993	2.16E-04	0.986	1.16E-03	0.969	3.93E-03
**Friedman mean rank**		**1.3203**		**4.7969**		**4.4219**		**2.5469**		**3.1172**		**4.8750**		**6.9219**	
**Rank**		**1**		**5**		**4**		**2**		**3**		**6**		**7**	

**Table 9:** The *FSIM* results for all algorithms.

Image Name	Level	HGJO		GJO		IGJO		RUN		AOA		DE		PSO	
		Mean	STD	Mean	STD	Mean	STD	Mean	STD	Mean	STD	Mean	STD	Mean	STD
C0080	8	**0.975**	1.33E-04	0.962	4.17E-03	0.970	2.03E-03	0.964	3.40E-03	0.971	1.18E-03	**0.975**	**3.94E-05**	0.885	2.12E-02
	16	**0.992**	**2.11E-04**	0.984	2.12E-03	0.987	2.02E-03	0.988	1.49E-03	0.990	9.56E-04	0.986	1.52E-03	0.939	1.04E-02
	24	**0.997**	5.07E-04	0.993	1.23E-03	0.993	1.33E-03	0.996	**4.77E-04**	0.996	6.36E-04	0.990	1.48E-03	0.977	3.62E-03
	32	**0.998**	**2.77E-04**	0.995	9.76E-04	0.993	1.17E-03	0.997	3.93E-04	**0.998**	4.65E-04	0.992	1.43E-03	0.992	1.91E-03
C0088	8	**0.988**	**1.61E-05**	0.986	6.69E-04	0.987	3.89E-04	0.987	3.53E-04	0.987	3.03E-04	**0.988**	3.22E-05	0.957	7.57E-03
	16	**0.997**	1.62E-04	0.995	6.08E-04	0.995	5.93E-04	0.995	3.75E-04	**0.997**	**9.38E-05**	0.995	6.02E-04	0.989	2.20E-03
	24	**0.999**	5.39E-05	0.997	5.21E-04	0.998	3.82E-04	0.998	2.77E-04	**0.999**	**5.21E-05**	0.998	3.63E-04	0.991	1.30E-03
	32	**0.999**	3.48E-05	0.998	3.44E-04	0.997	3.99E-04	0.998	1.92E-04	**0.999**	**3.25E-05**	0.998	1.97E-04	0.996	8.69E-04
C0132	8	0.972	3.60E-04	0.963	5.11E-03	0.974	1.56E-03	0.976	5.06E-04	**0.976**	2.05E-03	0.972	**3.03E-04**	0.951	9.26E-03
	16	**0.993**	**2.17E-04**	0.988	1.28E-03	0.989	1.69E-03	0.990	9.38E-04	0.991	9.38E-04	0.989	2.02E-03	0.977	6.35E-03
	24	**0.996**	**2.06E-04**	0.993	1.42E-03	0.995	1.09E-03	0.995	6.34E-04	0.996	4.78E-04	0.993	1.20E-03	0.983	3.29E-03
	32	**0.998**	2.79E-04	0.992	1.47E-03	0.996	9.72E-04	0.998	3.16E-04	**0.998**	**2.39E-04**	0.997	5.51E-04	0.990	2.11E-03
C0135	8	0.977	8.04E-06	0.968	2.00E-03	0.976	4.70E-04	0.976	4.25E-04	**0.978**	2.87E-04	0.977	**7.67E-06**	0.944	8.20E-03
	16	**0.995**	**1.73E-04**	0.987	1.87E-03	0.992	1.10E-03	0.990	9.83E-04	0.994	4.07E-04	0.989	1.27E-03	0.951	8.64E-03
	24	**0.998**	**1.60E-04**	0.994	9.36E-04	0.994	1.19E-03	0.996	4.94E-04	0.997	1.81E-04	0.995	6.62E-04	0.984	2.68E-03
	32	**0.999**	**9.61E-05**	0.997	7.34E-04	0.997	5.38E-04	0.997	3.68E-04	**0.999**	1.57E-04	0.996	6.31E-04	0.995	1.33E-03
C0180	8	**0.956**	**0.00E+00**	0.947	4.18E-03	0.948	2.06E-03	0.951	1.22E-03	0.955	3.55E-03	0.955	3.45E-04	0.917	2.50E-02
	16	**0.986**	**6.60E-04**	0.974	3.50E-03	0.978	3.18E-03	0.979	2.06E-03	0.980	1.59E-03	0.979	2.74E-03	0.961	8.50E-03
	24	**0.995**	**2.80E-04**	0.982	3.12E-03	0.985	2.72E-03	0.988	1.51E-03	0.987	1.59E-03	0.987	2.30E-03	0.954	1.14E-02
	32	**0.998**	**4.18E-04**	0.991	2.08E-03	0.992	1.16E-03	0.995	9.35E-04	0.992	1.19E-03	0.991	2.81E-03	0.967	6.64E-03
C0536	8	**0.947**	1.43E-04	0.941	7.41E-03	0.942	5.99E-03	0.947	6.61E-03	**0.947**	2.75E-03	0.947	**4.55E-16**	0.934	1.08E-02
	16	**0.988**	1.77E-03	0.981	2.34E-03	0.983	2.10E-03	0.985	1.29E-03	0.983	**1.02E-03**	0.981	4.16E-03	0.967	8.89E-03
	24	**0.996**	**7.85E-04**	0.990	2.24E-03	0.990	1.86E-03	0.992	1.19E-03	0.989	8.61E-04	0.986	2.58E-03	0.972	9.37E-03
	32	**0.997**	**2.70E-04**	0.990	2.03E-03	0.995	1.17E-03	0.995	6.55E-04	0.995	7.87E-04	0.991	1.78E-03	0.979	3.89E-03
C1088	8	**0.951**	8.77E-04	0.928	8.46E-03	0.921	8.23E-03	0.937	5.06E-03	0.947	1.83E-03	0.949	**4.13E-04**	0.799	4.52E-02
	16	**0.990**	**6.26E-04**	0.963	6.40E-03	0.975	4.30E-03	0.985	1.73E-03	0.983	2.61E-03	0.975	4.34E-03	0.878	2.48E-02
	24	**0.996**	**4.42E-04**	0.982	3.54E-03	0.989	1.61E-03	0.992	1.46E-03	0.994	1.00E-03	0.994	2.96E-03	0.979	1.06E-02
	32	**0.998**	**2.02E-04**	0.990	1.57E-03	0.993	1.68E-03	0.993	1.31E-03	0.996	7.79E-04	0.994	1.83E-03	0.971	7.71E-03
L0032	8	**0.921**	6.75E-04	0.917	1.76E-03	0.917	1.57E-03	0.917	1.25E-02	0.919	1.96E-03	0.919	**1.44E-04**	0.884	3.37E-02
	16	**0.943**	**1.93E-03**	0.917	2.62E-02	0.923	2.59E-02	0.927	1.88E-02	0.924	2.33E-02	0.924	9.88E-03	0.926	2.44E-02
	24	0.980	8.13E-03	0.944	1.74E-02	0.932	2.61E-02	**0.994**	**2.82E-03**	0.900	2.24E-02	0.972	2.24E-02	0.918	3.44E-02
	32	**0.997**	**5.54E-03**	0.955	9.36E-03	0.974	7.09E-03	0.947	1.52E-02	0.958	1.34E-02	0.829	3.63E-02	0.939	2.12E-02
L0064	8	**0.969**	5.92E-04	0.965	1.73E-03	0.968	1.98E-03	0.958	4.17E-03	0.967	1.31E-03	0.967	**5.74E-04**	0.944	1.07E-02
	16	0.993	6.84E-04	0.985	3.19E-03	0.991	1.23E-03	**0.995**	**4.15E-04**	0.990	2.22E-03	0.983	4.46E-03	0.980	7.06E-03
	24	**0.997**	**3.60E-04**	0.991	1.65E-03	0.994	1.10E-03	**0.997**	4.47E-04	**0.997**	9.12E-04	0.996	1.80E-03	0.975	7.58E-03
	32	**0.999**	**2.20E-04**	0.998	6.91E-04	0.997	6.62E-04	**0.999**	3.46E-04	0.998	2.71E-04	0.996	2.42E-03	0.990	5.83E-03
L0135	8	**0.967**	4.77E-04	0.950	4.98E-03	0.963	3.52E-03	0.950	5.04E-03	**0.967**	5.39E-04	**0.967**	**4.28E-04**	0.865	3.01E-02
	16	0.992	**1.15E-03**	0.978	3.88E-03	0.972	5.53E-03	**0.993**	1.86E-03	0.982	4.50E-03	0.979	4.13E-03	0.952	9.37E-03
	24	**0.998**	**6.89E-04**	0.992	1.43E-03	0.991	1.36E-03	0.995	9.63E-04	0.995	8.49E-04	0.990	2.44E-03	0.981	7.88E-03
	32	**0.999**	**3.56E-04**	0.996	7.33E-04	0.996	7.45E-04	0.994	1.70E-03	0.995	8.54E-04	0.987	2.41E-03	0.984	6.54E-03
L0158	8	0.964	5.09E-04	0.948	7.99E-03	0.954	3.67E-03	**0.967**	5.80E-03	0.960	3.09E-03	0.962	**0.00E+00**	0.921	1.65E-02
	16	0.992	1.14E-03	0.983	3.95E-03	0.981	5.72E-03	**0.996**	**7.57E-04**	0.994	1.24E-03	0.988	6.61E-03	0.959	1.48E-02
	24	**0.998**	7.64E-04	0.994	1.22E-03	0.991	2.42E-03	**0.998**	**2.76E-04**	**0.998**	3.94E-04	0.990	2.85E-03	0.977	1.17E-02
	32	**0.999**	3.70E-04	0.998	**3.13E-04**	0.994	1.37E-03	0.997	4.39E-04	0.998	3.72E-04	0.992	1.80E-03	0.984	3.77E-03
L0226	8	0.960	**0.00E+00**	0.941	5.84E-03	**0.963**	1.44E-03	0.953	2.15E-03	0.961	1.00E-03	0.961	2.56E-04	0.876	2.49E-02
	16	**0.990**	**8.55E-04**	0.986	2.51E-03	0.986	2.03E-03	0.987	1.25E-03	0.984	3.05E-03	0.989	3.25E-03	0.972	8.56E-03
	24	**0.998**	**4.03E-04**	0.994	1.01E-03	0.991	1.49E-03	0.995	7.68E-04	0.996	6.68E-04	0.988	2.81E-03	0.981	7.68E-03
	32	**0.999**	**1.09E-04**	0.995	1.05E-03	0.996	6.03E-04	0.997	4.02E-04	0.997	3.57E-04	0.995	1.75E-03	0.991	3.28E-03
L0699	8	0.943	**0.00E+00**	0.918	9.03E-03	0.937	3.67E-03	0.936	9.32E-03	0.937	6.95E-03	0.943	**0.00E+00**	**0.948**	2.85E-02
	16	**0.986**	**2.34E-03**	0.960	8.36E-03	0.973	5.58E-03	0.968	6.71E-03	0.974	5.98E-03	0.958	8.86E-03	0.923	2.14E-02
	24	**0.997**	**9.79E-04**	0.987	3.22E-03	0.985	3.11E-03	0.981	4.08E-03	0.990	2.14E-03	0.979	6.18E-03	0.935	1.77E-02
	32	**0.999**	**2.49E-04**	0.994	1.88E-03	0.993	2.09E-03	0.995	8.85E-04	**0.999**	7.23E-04	0.982	5.05E-03	0.953	1.18E-02
L0879	8	**0.962**	7.77E-05	0.955	4.93E-03	0.944	8.41E-03	0.955	8.43E-03	**0.962**	1.80E-03	0.962	**2.28E-16**	0.958	2.30E-02
	16	0.993	1.05E-03	0.987	2.93E-03	0.985	3.04E-03	**0.994**	**8.56E-04**	0.984	3.28E-03	0.987	3.62E-03	0.975	1.08E-02
	24	**0.998**	5.37E-04	0.995	9.02E-04	0.983	3.30E-03	0.997	**4.97E-04**	0.992	1.82E-03	0.985	2.70E-03	0.958	9.37E-03
	32	**0.999**	3.64E-04	0.994	1.08E-03	0.997	5.13E-04	0.998	1.49E-03	0.998	**2.75E-04**	0.995	1.86E-03	0.990	6.74E-03
L1074	8	**0.983**	1.08E-04	0.978	2.66E-03	0.978	1.74E-03	**0.983**	7.57E-04	**0.983**	8.91E-04	**0.983**	**0.00E+00**	0.982	8.03E-03
	16	**0.996**	**2.65E-04**	0.994	8.05E-04	0.993	1.17E-03	**0.996**	5.68E-04	**0.996**	6.06E-04	0.994	1.36E-03	0.989	3.70E-03
	24	**0.999**	1.15E-04	0.998	6.61E-04	0.998	6.14E-04	0.997	3.67E-04	**0.999**	**9.92E-05**	0.996	9.59E-04	0.995	1.89E-03
	32	**0.999**	7.77E-05	0.999	3.86E-04	0.998	5.77E-04	**0.999**	1.52E-04	**0.999**	**4.11E-05**	0.998	3.90E-04	0.994	1.60E-03
X0017	8	**0.984**	**4.13E-05**	0.979	1.94E-03	0.979	1.70E-03	0.980	1.13E-03	**0.984**	2.07E-04	**0.984**	9.19E-05	0.968	4.20E-03
	16	**0.996**	**1.18E-04**	0.990	1.52E-03	0.993	8.46E-04	0.993	8.67E-04	0.995	1.69E-04	0.991	9.11E-04	0.981	3.24E-03
	24	**0.998**	1.31E-04	0.996	3.31E-04	0.996	5.48E-04	0.996	3.13E-04	**0.998**	**1.13E-04**	0.995	6.34E-04	0.990	1.91E-03
	32	**0.999**	7.92E-05	0.997	3.82E-04	0.997	4.43E-04	0.998	2.82E-04	**0.999**	**5.66E-05**	0.997	3.27E-04	0.990	1.43E-03
**Friedman mean rank**		**1.5547**		**5.0938**		**4.4531**		**3.2031**		**2.6484**		**4.3125**		**6.7344**	
**Rank**		**1**		**6**		**5**		**3**		**2**		**4**		**7**	

According to the analysis of the results of *PSNR* recorded in Table 7, the proposed HGJO algorithm obtained the best experimental data in terms of accuracy and stability in the segmentation experiments of C0080, C0088, C0132, C0135, C0180, C1088, L0226, L0699 and X0017 for a total of 9 images. The other compared algorithms did not obtain the best results in the segmentation experiments for any of the images. The top three algorithms ranked by the Friedman mean rank test are as follows: the first ranked is the proposed HGJO algorithm, the AOA algorithm ranks second, and the RUN ranks third.

In Table 8, which records the results about *SSIM*, the proposed algorithm achieves optimal results in the segmentation experiments of C0080, C0536, and X0017 for a total of 3 images. The rest of the algorithms also did not achieve optimal results on any of the images. According to the Friedman mean rank test, the top three algorithms were, HGJO, RUN, and AOA.

Table 9 shows the experimental results of *FSIM*, which are similar to Tables 7 and 8. By evaluating the *FSIM* metrics, the proposed HGJO algorithm achieves optimal results in a total of 7 images, C0080, C0088, C0180, C0536, C1088, L1074, and X0017. The performance far exceeds that of other comparable algorithms. According to the Friedman average ranking test, the top three algorithms are HGJO, AOA, and RUN.

In addition, Table 10 shows the results of the Wilcoxon rank-sum test. Consistent with the evaluation criteria in the previous section, if *P*>0.05, the null hypothesis is true; otherwise, the alternative hypothesis is true. By analyzing the results of the Wilcoxon rank-sum test, we can see that the proposed method has significant differences with other compared algorithms in all experiments, except for some experiments where it performs similarly to DE(OBL). Therefore, we can conclude that the performance of HGJO is significantly different from other algorithms.

**Table 10:** Comparison of the Wilcoxon signed-rank test for Otsu method.

Image Name	Level	HGJO vs. GJO	HGJO vs. IGJO	HGJO vs. RUN	HGJO vs. AOA	HGJO vs. DE	HGJO vs. PSO
C0080	8	3.090E-05 **++**	3.090E-05 **++**	3.090E-05 **++**	3.090E-05 **++**	3.906E-03 **++**	3.090E-05 **++**
	16	3.090E-05 **++**	3.090E-05 **++**	3.090E-05 **++**	3.090E-05 **++**	3.090E-05 **++**	3.090E-05 **++**
	24	3.090E-05 **++**	3.090E-05 **++**	3.090E-05 **++**	3.090E-05 **++**	3.090E-05 **++**	3.090E-05 **++**
	32	3.090E-05 **++**	3.090E-05 **++**	3.090E-05 **++**	3.090E-05 **++**	3.090E-05 **++**	3.090E-05 **++**
C0088	8	3.090E-05 **++**	3.090E-05 **++**	3.090E-05 **++**	3.090E-05 **++**	1.734E-04 **++**	3.090E-05 **++**
	16	3.090E-05 **++**	3.090E-05 **++**	3.090E-05 **++**	3.090E-05 **++**	3.090E-05 **++**	3.090E-05 **++**
	24	3.090E-05 **++**	3.090E-05 **++**	3.090E-05 **++**	3.090E-05 **++**	3.090E-05 **++**	3.090E-05 **++**
	32	3.090E-05 **++**	3.090E-05 **++**	3.090E-05 **++**	3.090E-05 **++**	3.090E-05 **++**	3.090E-05 **++**
C0132	8	3.090E-05 **++**	3.090E-05 **++**	3.090E-05 **++**	3.578E-05 **++**	6.104E-04 **++**	3.090E-05 **++**
	16	3.090E-05 **++**	3.090E-05 **++**	3.090E-05 **++**	3.090E-05 **++**	3.090E-05 **++**	3.090E-05 **++**
	24	3.090E-05 **++**	3.090E-05 **++**	3.090E-05 **++**	3.090E-05 **++**	3.090E-05 **++**	3.090E-05 **++**
	32	3.090E-05 **++**	3.090E-05 **++**	3.090E-05 **++**	3.090E-05 **++**	3.090E-05 **++**	3.090E-05 **++**
C0135	8	3.090E-05 **++**	3.090E-05 **++**	3.090E-05 **++**	3.090E-05 **++**	1.090E-04 **++**	3.090E-05 **++**
	16	3.090E-05 **++**	3.090E-05 **++**	3.090E-05 **++**	3.090E-05 **++**	3.090E-05 **++**	3.090E-05 **++**
	24	3.090E-05 **++**	3.090E-05 **++**	3.090E-05 **++**	3.090E-05 **++**	3.090E-05 **++**	3.090E-05 **++**
	32	3.090E-05 **++**	3.090E-05 **++**	3.090E-05 **++**	3.090E-05 **++**	3.090E-05 **++**	3.090E-05 **++**
C0180	8	3.090E-05 **++**	3.090E-05 **++**	3.090E-05 **++**	3.090E-05 **++**	**9.766E-02 —**	3.090E-05 **++**
	16	3.090E-05 **++**	3.090E-05 **++**	3.090E-05 **++**	3.090E-05 **++**	3.090E-05 **++**	3.090E-05 **++**
	24	3.090E-05 **++**	3.090E-05 **++**	3.090E-05 **++**	3.090E-05 **++**	3.090E-05 **++**	3.090E-05 **++**
	32	3.090E-05 **++**	3.090E-05 **++**	3.090E-05 **++**	3.090E-05 **++**	3.090E-05 **++**	3.090E-05 **++**
C0536	8	3.090E-05 **++**	3.090E-05 **++**	3.090E-05 **++**	3.090E-05 **++**	8.411E-05 **++**	3.090E-05 **++**
	16	3.090E-05 **++**	3.090E-05 **++**	3.090E-05 **++**	3.090E-05 **++**	3.090E-05 **++**	3.090E-05 **++**
	24	3.090E-05 **++**	3.090E-05 **++**	3.090E-05 **++**	3.090E-05 **++**	3.090E-05 **++**	3.090E-05 **++**
	32	3.090E-05 **++**	3.090E-05 **++**	3.090E-05 **++**	3.090E-05 **++**	3.090E-05 **++**	3.090E-05 **++**
C1088	8	3.090E-05 **++**	3.090E-05 **++**	3.090E-05 **++**	3.090E-05 **++**	8.731E-03 **++**	3.090E-05 **++**
	16	3.090E-05 **++**	3.090E-05 **++**	3.090E-05 **++**	3.090E-05 **++**	3.090E-05 **++**	3.090E-05 **++**
	24	3.090E-05 **++**	3.090E-05 **++**	3.090E-05 **++**	3.090E-05 **++**	3.090E-05 **++**	3.090E-05 **++**
	32	3.090E-05 **++**	3.090E-05 **++**	3.090E-05 **++**	3.090E-05 **++**	3.090E-05 **++**	3.090E-05 **++**
L0032	8	3.090E-05 **++**	3.090E-05 **++**	3.090E-05 **++**	3.090E-05 **++**	**1.289E-01 —**	3.090E-05 **++**
	16	3.090E-05 **++**	3.090E-05 **++**	3.090E-05 **++**	3.090E-05 **++**	3.090E-05 **++**	3.090E-05 **++**
	24	3.090E-05 **++**	3.090E-05 **++**	3.090E-05 **++**	3.090E-05 **++**	3.090E-05 **++**	2.875E-05 **++**
	32	3.090E-05 **++**	3.090E-05 **++**	3.090E-05 **++**	2.643E-05 **++**	3.051E-05 **++**	2.489E-05 **++**
L0064	8	3.090E-05 **++**	3.090E-05 **++**	3.090E-05 **++**	3.090E-05 **++**	**6.168E-02 —**	3.090E-05 **++**
	16	3.090E-05 **++**	3.090E-05 **++**	3.090E-05 **++**	3.090E-05 **++**	3.090E-05 **++**	3.090E-05 **++**
	24	3.090E-05 **++**	3.090E-05 **++**	3.090E-05 **++**	3.090E-05 **++**	3.090E-05 **++**	3.090E-05 **++**
	32	3.090E-05 **++**	3.090E-05 **++**	3.090E-05 **++**	3.090E-05 **++**	3.090E-05 **++**	3.090E-05 **++**
L0135	8	3.090E-05 **++**	3.090E-05 **++**	3.090E-05 **++**	3.090E-05 **++**	8.356E-04 **++**	3.090E-05 **++**
	16	3.090E-05 **++**	3.090E-05 **++**	3.090E-05 **++**	3.090E-05 **++**	3.090E-05 **++**	3.090E-05 **++**
	24	3.090E-05 **++**	3.090E-05 **++**	3.090E-05 **++**	3.090E-05 **++**	3.090E-05 **++**	3.090E-05 **++**
	32	3.090E-05 **++**	3.090E-05 **++**	3.090E-05 **++**	3.090E-05 **++**	3.090E-05 **++**	3.090E-05 **++**
L0158	8	3.090E-05 **++**	3.090E-05 **++**	3.090E-05 **++**	3.090E-05 **++**	1.367E-02 **++**	3.090E-05 **++**
	16	3.090E-05 **++**	3.090E-05 **++**	3.090E-05 **++**	3.090E-05 **++**	3.090E-05 **++**	3.090E-05 **++**
	24	3.090E-05 **++**	3.090E-05 **++**	3.090E-05 **++**	3.090E-05 **++**	3.090E-05 **++**	3.090E-05 **++**
	32	3.090E-05 **++**	3.090E-05 **++**	3.090E-05 **++**	3.090E-05 **++**	3.090E-05 **++**	3.090E-05 **++**
L0226	8	3.090E-05 **++**	3.090E-05 **++**	3.090E-05 **++**	3.090E-05 **++**	**NaN —**	3.090E-05 **++**
	16	3.090E-05 **++**	3.090E-05 **++**	3.090E-05 **++**	3.090E-05 **++**	3.090E-05 **++**	3.090E-05 **++**
	24	3.090E-05 **++**	3.090E-05 **++**	3.090E-05 **++**	3.090E-05 **++**	3.090E-05 **++**	3.090E-05 **++**
	32	3.090E-05 **++**	3.090E-05 **++**	3.090E-05 **++**	3.090E-05 **++**	3.090E-05 **++**	3.090E-05 **++**
L0699	8	3.090E-05 **++**	3.090E-05 **++**	3.090E-05 **++**	3.090E-05 **++**	4.648E-01 **--**	3.090E-05 **++**
	16	3.090E-05 **++**	3.090E-05 **++**	3.090E-05 **++**	3.090E-05 **++**	3.090E-05 **++**	3.090E-05 **++**
	24	3.090E-05 **++**	3.090E-05 **++**	3.090E-05 **++**	3.090E-05 **++**	3.090E-05 **++**	3.090E-05 **++**
	32	3.090E-05 **++**	3.090E-05 **++**	3.090E-05 **++**	3.090E-05 **++**	3.090E-05 **++**	3.090E-05 **++**
L0879	8	3.090E-05 **++**	3.090E-05 **++**	3.090E-05 **++**	3.090E-05 **++**	9.766E-04 **++**	3.090E-05 **++**
	16	3.090E-05 **++**	3.090E-05 **++**	3.090E-05 **++**	3.090E-05 **++**	3.090E-05 **++**	3.090E-05 **++**
	24	3.090E-05 **++**	3.090E-05 **++**	3.090E-05 **++**	3.090E-05 **++**	3.090E-05 **++**	3.090E-05 **++**
	32	3.090E-05 **++**	3.090E-05 **++**	3.090E-05 **++**	3.090E-05 **++**	3.090E-05 **++**	3.090E-05 **++**
L1074	8	3.090E-05 **++**	3.090E-05 **++**	3.090E-05 **++**	3.090E-05 **++**	**6.250E-02 —**	3.090E-05 **++**
	16	3.090E-05 **++**	3.090E-05 **++**	3.090E-05 **++**	3.090E-05 **++**	3.090E-05 **++**	3.090E-05 **++**
	24	3.090E-05 **++**	3.090E-05 **++**	3.090E-05 **++**	3.090E-05 **++**	3.090E-05 **++**	3.090E-05 **++**
	32	3.090E-05 **++**	3.090E-05 **++**	3.090E-05 **++**	3.090E-05 **++**	3.090E-05 **++**	3.090E-05 **++**
X0017	8	3.090E-05 **++**	3.090E-05 **++**	3.090E-05 **++**	3.090E-05 **++**	5.662E-04 **++**	3.090E-05 **++**
	16	3.090E-05 **++**	3.090E-05 **++**	3.090E-05 **++**	3.090E-05 **++**	3.090E-05 **++**	3.090E-05 **++**
	24	3.090E-05 **++**	3.090E-05 **++**	3.090E-05 **++**	3.090E-05 **++**	3.090E-05 **++**	3.090E-05 **++**
	32	3.090E-05 **++**	3.090E-05 **++**	3.090E-05 **++**	3.090E-05 **++**	3.090E-05 **++**	3.090E-05 **++**

Despite the fact that the algorithms used for comparison have some competitiveness on some images. In all, however, the proposed algorithm is still outstanding in optical aerial image segmentation. These results demonstrate that the HGJO algorithm can achieve better results in processing optical aerial images. Combined with the analysis of the results for CEC2022 test suite in the previous section, it is reasonable to presume that the current results can be maintained when HGJO is applied to a wider range of optical aerial image segmentation in the future. In addition, an interesting phenomenon worth our attention is that the improved algorithm has a certain loss in the performance of land images which is named at the beginning of “L”. By comparing with the original image, we can observe that these images all have a common feature, including many small objects, which greatly increases the difficulty of segmentation. Therefore, in the future work, we can further optimize the segmentation problem of such images.

**Fig 15:**
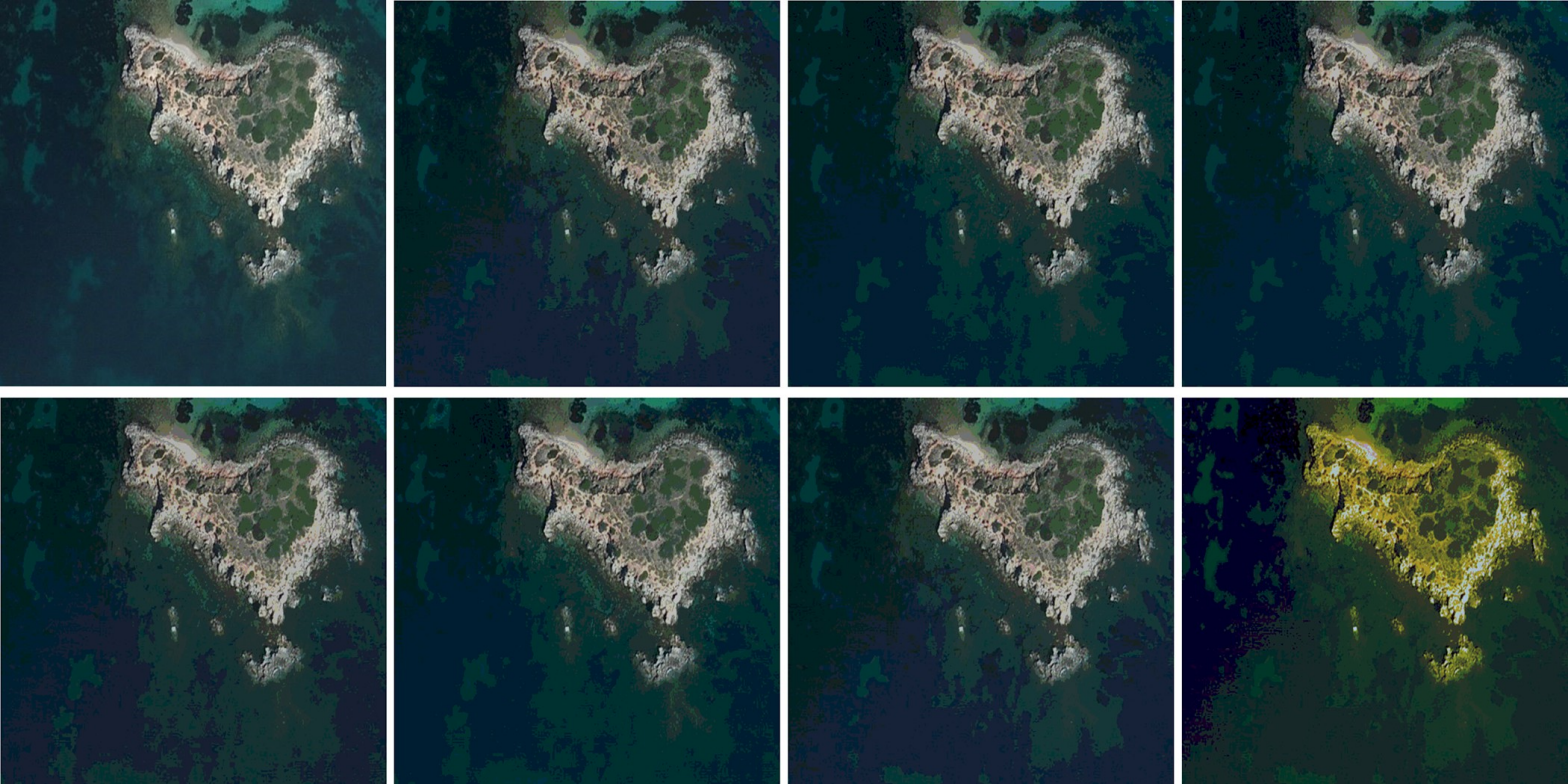
The segmentation results for C0180.

Fig 15 displays the segmentation results of each algorithm on the test image C0180 with a threshold level of 8. They are the original image, HGJO segmentation result, GJO segmentation result, IGJO segmentation result, RUN segmentation result, AOA segmentation result, DE(OBL) segmentation result and PSO(OBL) segmentation result in order. It is worth noting that only the image results with a threshold level of 8 are shown here, because it is impossible to intuitively feel the quality of the segmentation results in the high threshold segmentation results through human eye observation. Therefore, image results with a threshold level above 8 are not listed separately. With this figure, we can intuitively feel that the island in the optical aerial image segmented by HGJO have more distinct contours. In addition, by comparing the seabed distribution features in the segmentation results, we can observe that the competition algorithms ignore these details. However, HGJO preserves almost all the features of ocean distribution. Therefore, it can be concluded that HGJO can effectively segment complex ocean distribution optical aerial images with high quality. In summary, the proposed HGJO can effectively handle optical aerial image segmentation and provide effective help for the subsequent data processing and data acquisition.

Furthermore, the segmentation histogram for each channel is also shown in Appendix A–G in S1 Appendix. By comparing these images, we can see that, the results of HGJO segmentation on the red, green, and blue channels can retain more details. This shows that for optical aerial images, the proposed algorithm has more advantages and can retain more details after segmentation than other algorithms, which is helpful for subsequent image processing.

## 6. Conclusions

Aerial photography images can provide a wealth of information for scientific researchers. Multi-level threshold segmentation of aerial images can effectively reduce the complexity of subsequent image processing while preserving the original features. This paper introduces a new optimization algorithm, the Golden Jackal Optimization (GJO) algorithm, which suffers from convergence issues and the tendency to get stuck in local optima. Therefore, an improved version of GJO, which is named HGJO, is proposed in this paper to enhance the search capabilities and avoid getting stuck in local optima, to optimize the process of multilevel thresholding segmentation. The performance of the proposed method is compared with six different meta-heuristics, including GJO, IGJO, RUN, AOA, DE(OBL), and PSO(OBL), on the IEEE CEC2022 benchmark test function. Based on the experimental results, the proposed algorithm outperforms all other algorithms in terms of convergence accuracy and stability. In addition, the Otsu method is used as an objective function to perform multi-level threshold segmentation on a set of aerial images. *PSNR*, *SSIM*, and *FSIM* are used as evaluation metrics to assess the quality of the segmented images produced by each algorithm. Moreover, the Friedman mean rank test and the Wilcoxon rank-sum test are used to verify the segmentation results. The experimental results show that HGJO outperforms other algorithms in terms of overall performance. The proposed algorithm can effectively reduce the image complexity while preserving the original features, thereby improving the efficiency of the subsequent image processing. In general, the results of this study are satisfactory, but there are certain shortcomings. Firstly, the introduction of OBL and Cauchy operators increases the computation time of the original algorithm, making the proposed method less efficient than most algorithms (only better than RUN). Secondly, there is a performance loss in complex image segmentation, such as test images L0032, L0064, and L0135, thus further work is needed to improve the algorithm’s performance in these types of image segmentation.

In future work, we will further validate and improve the proposed algorithm through more extensive problems, such as medical image segmentation. In addition, we will try to apply HGJO to other more complex problems, such as neural networks, remote sensing data processing, and UAV path analysis. Furthermore, improving the computational efficiency of HGJO would be a significant contribution.

## Supporting information

S1 Appendix (DOCX)

S1 File (ZIP)
