# Supplementary material for: An improved golden jackal optimization for multilevel thresholding image segmentation
Source: PLoS One. 2023 May 5;18(5):e0285211. doi: 10.1371/journal.pone.0285211 (PMC10162520; doi:10.1371/journal.pone.0285211)
Supplement: S1 Appendix — (DOCX) [file pone.0285211.s001.docx]

Appendix A


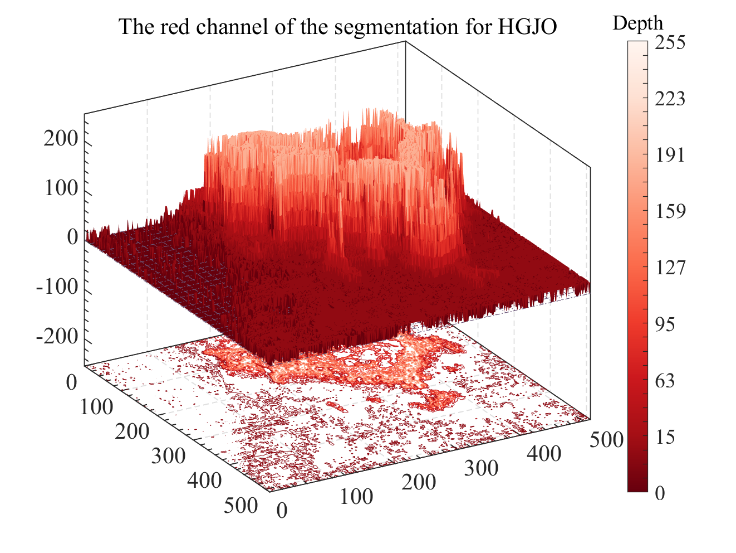

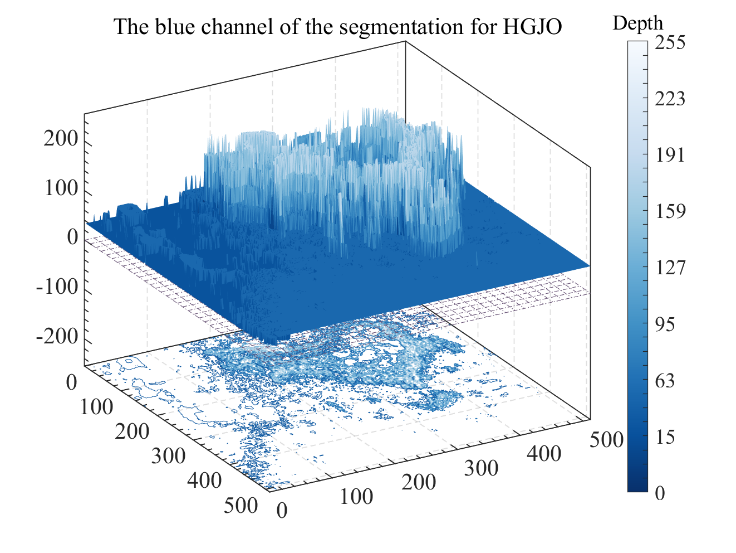

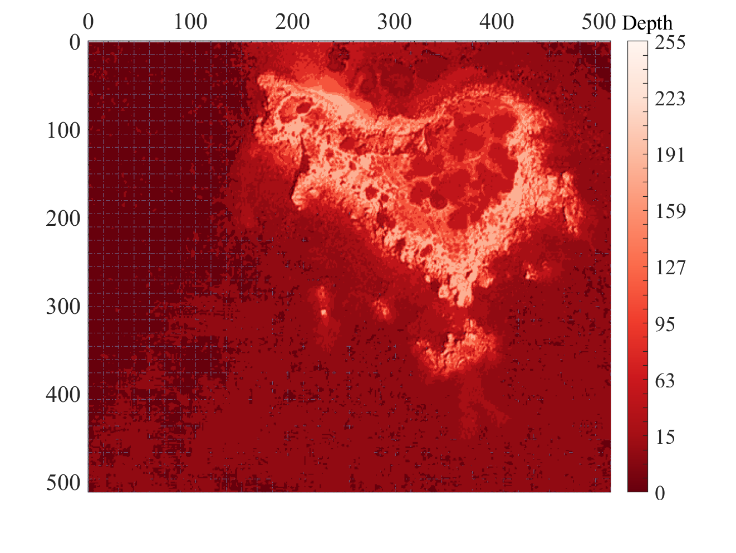

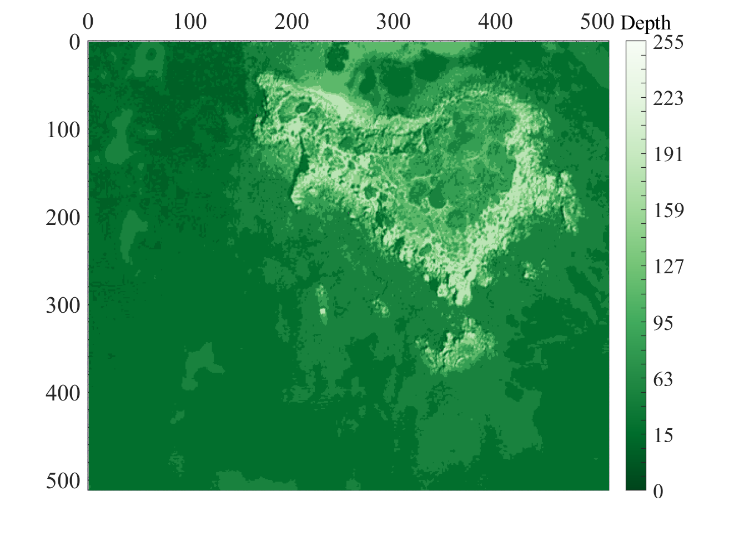

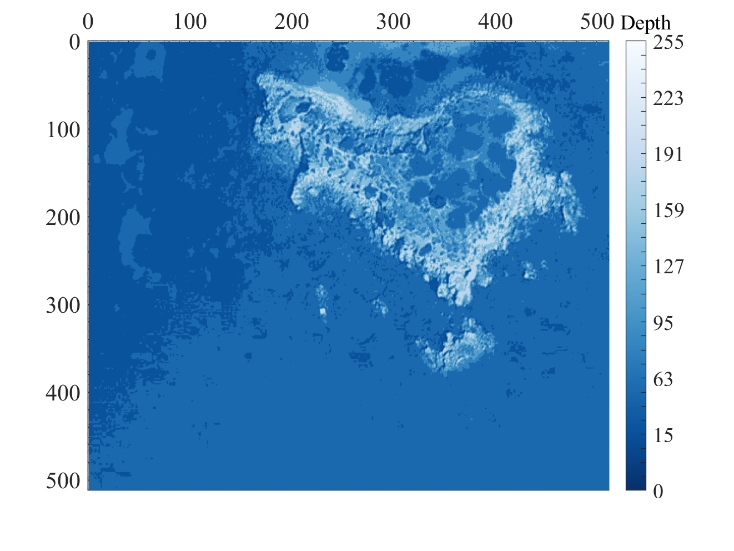


**Fig. 16** The 3D histograms and the projection for HGJO.

Appendix B


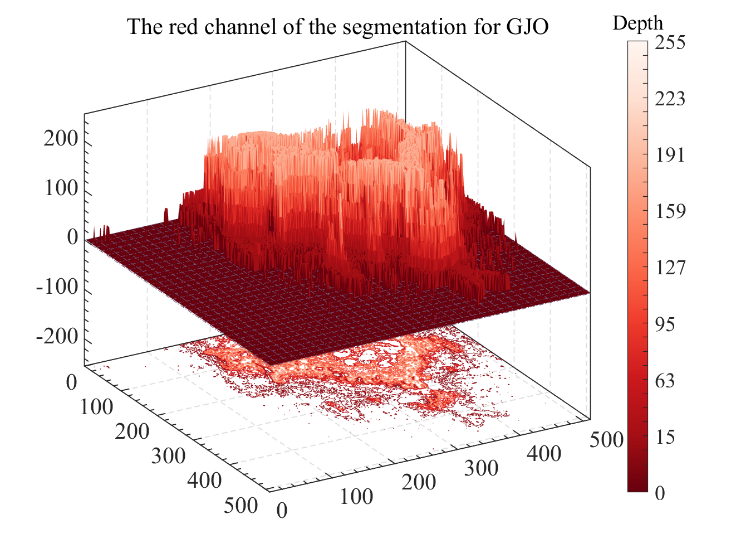

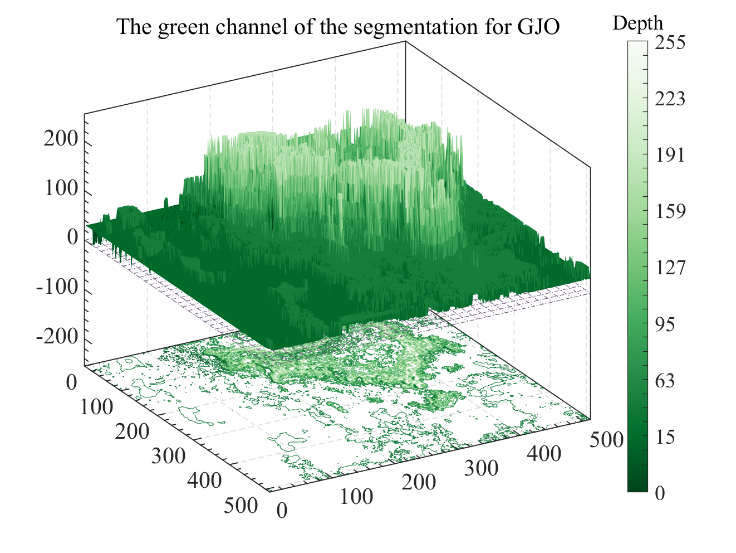

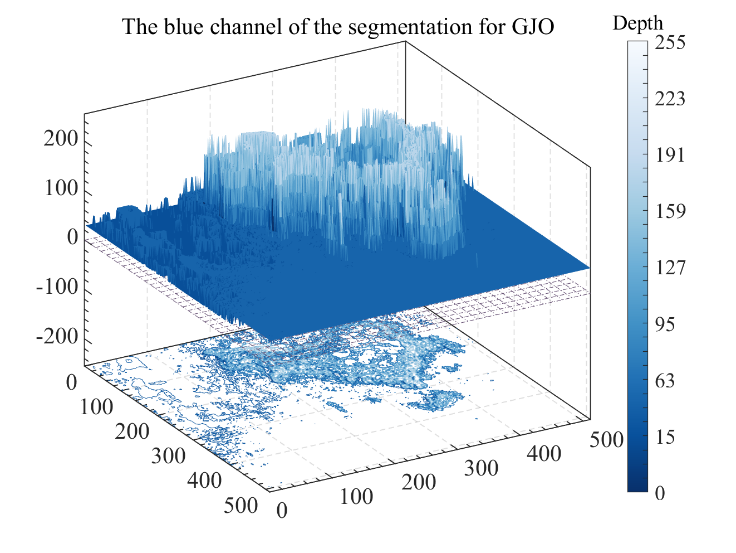

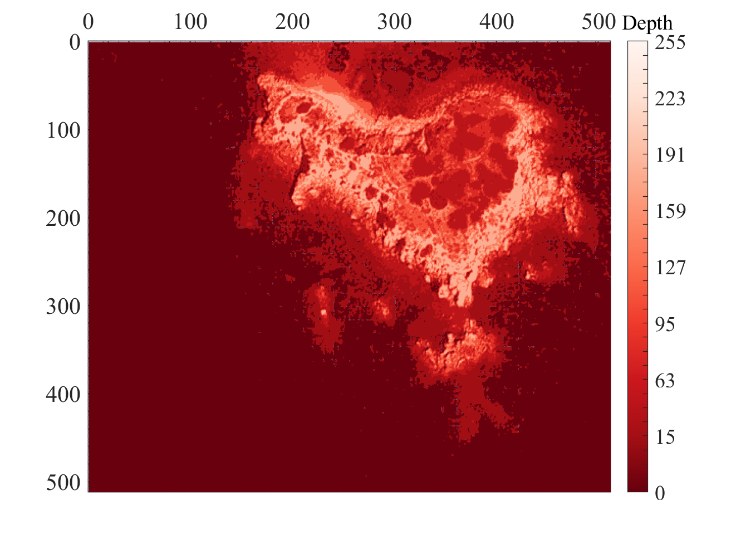

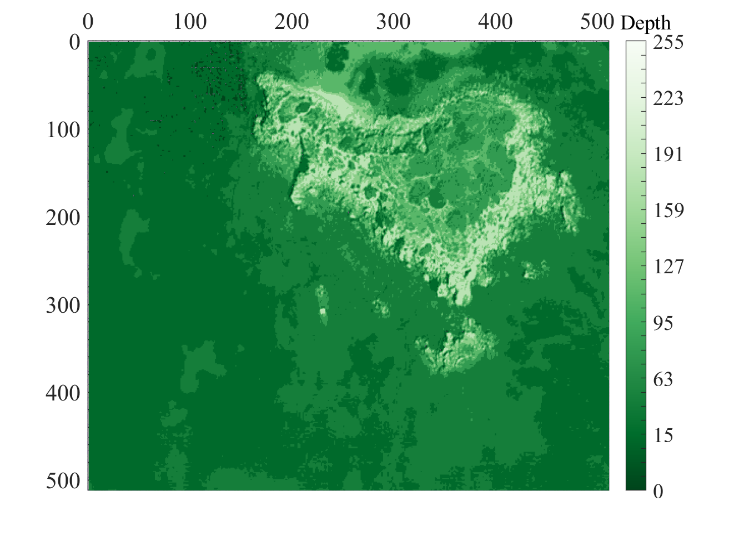

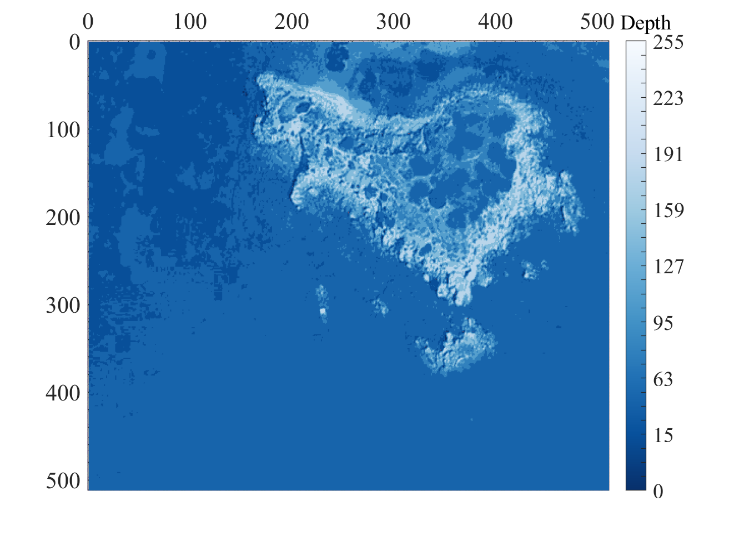


**Fig. 17** The 3D histograms and the projection for GJO.

Appendix C


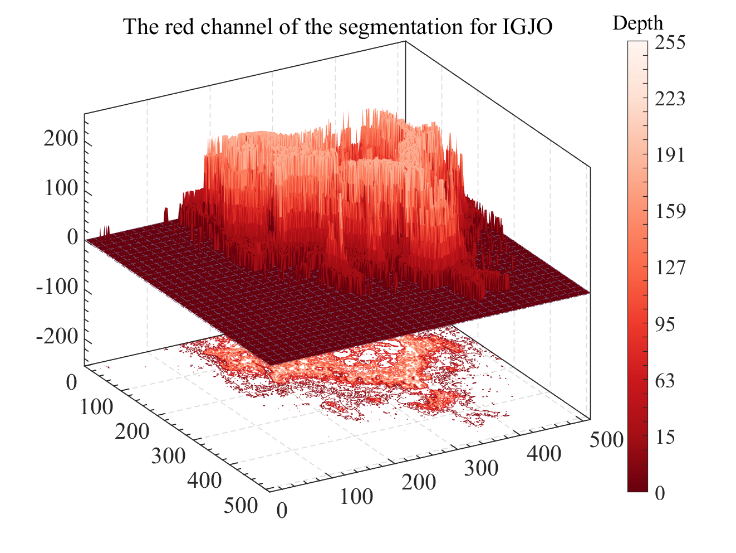

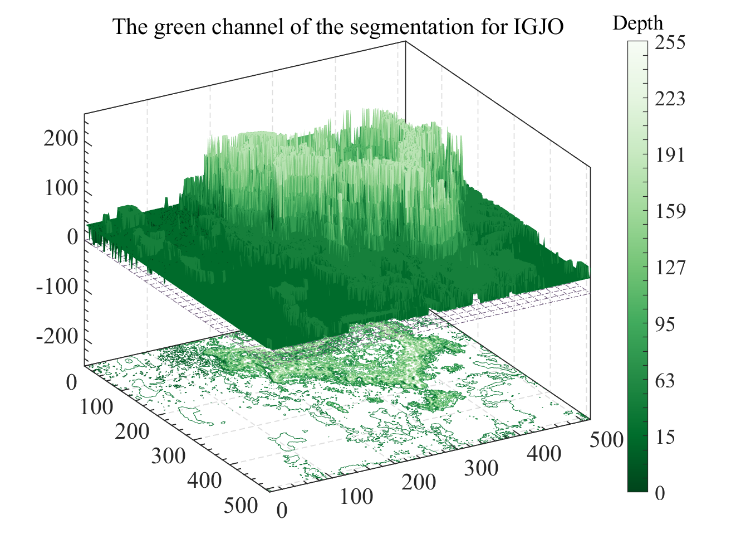

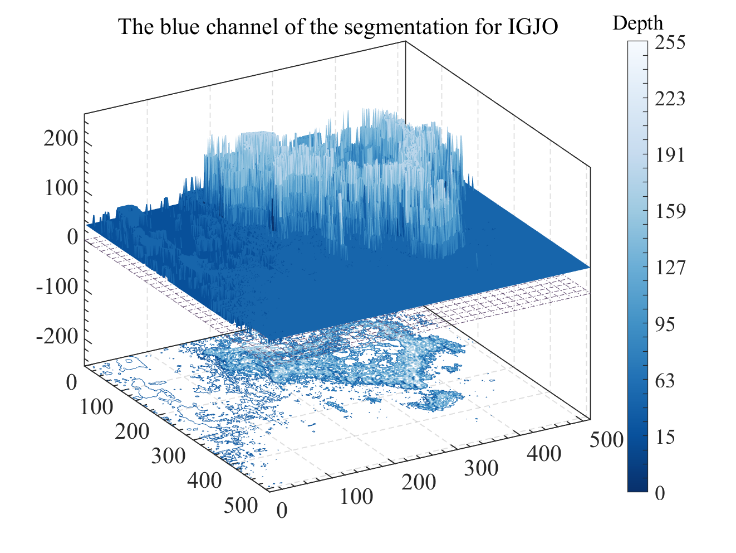

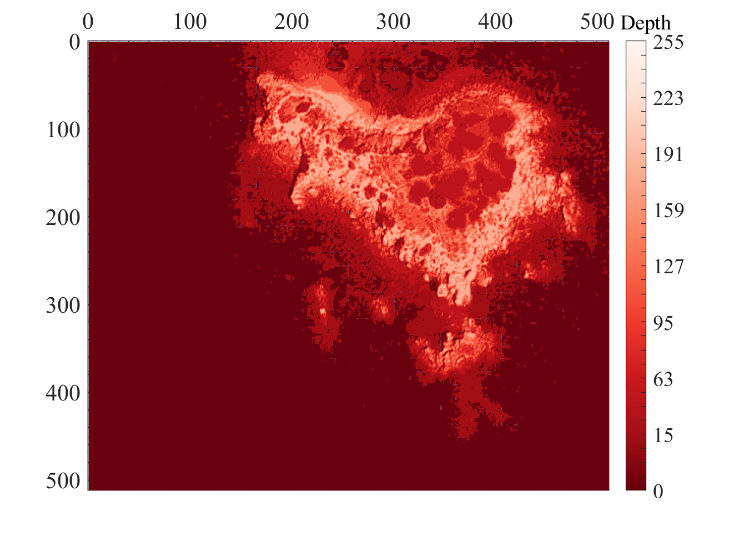

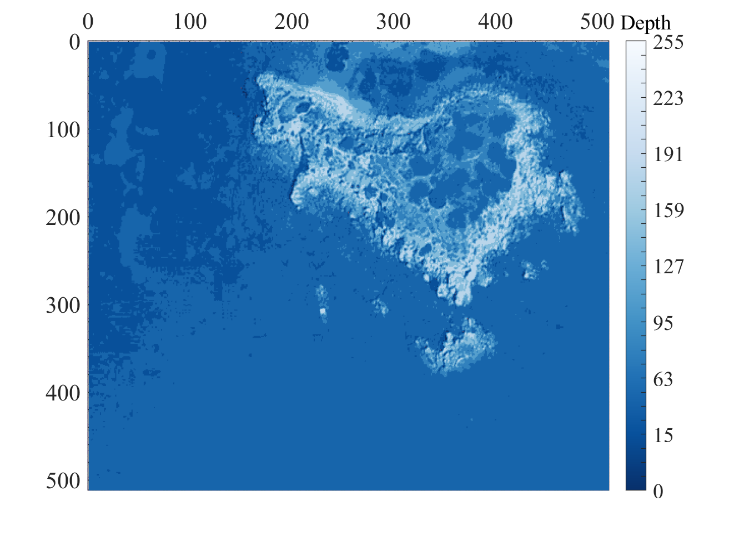

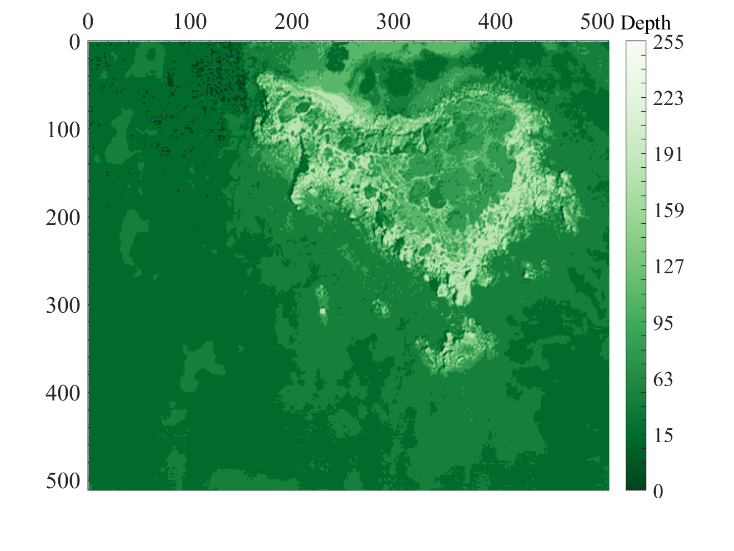


**Fig. 18** The 3D histograms and the projection for IGJO.

Appendix D


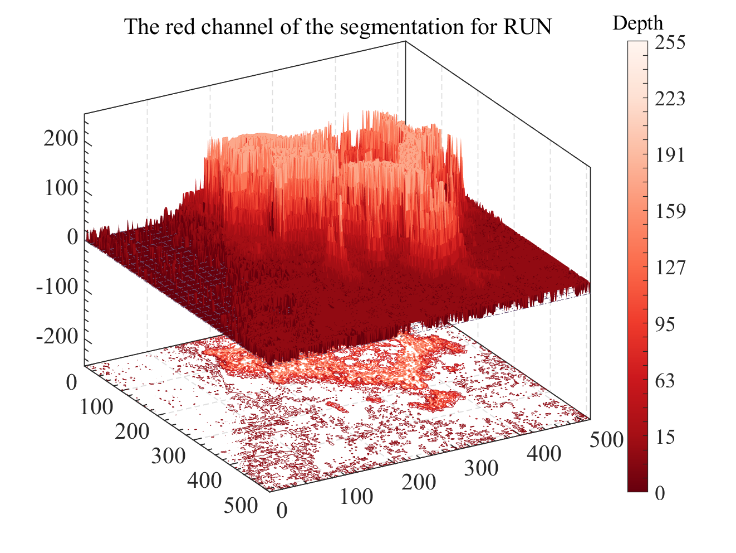

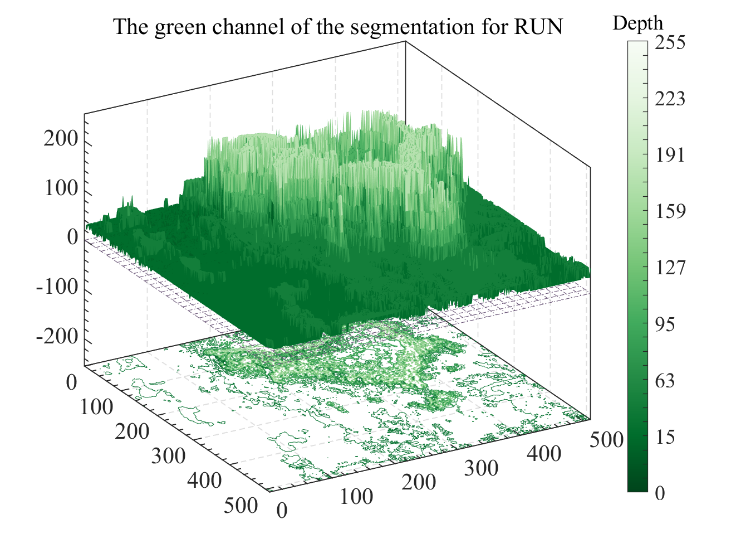

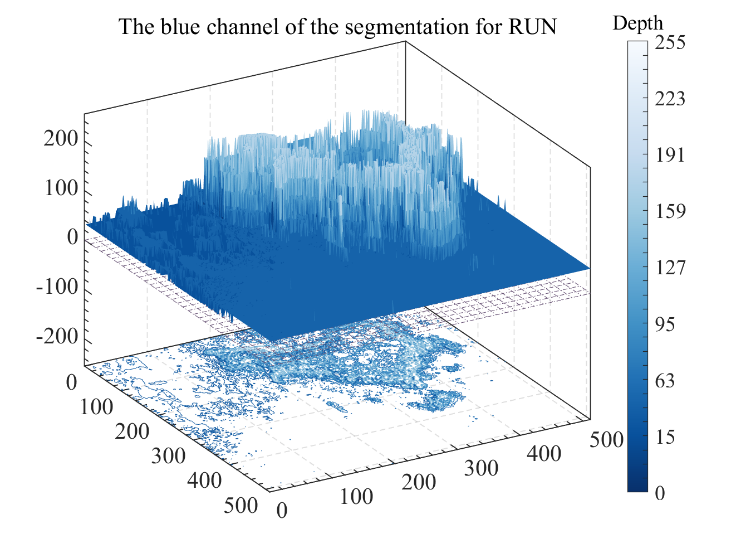

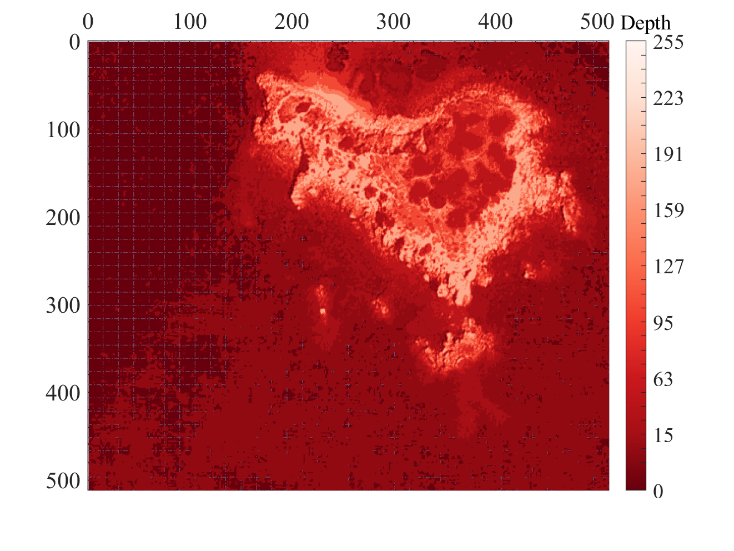

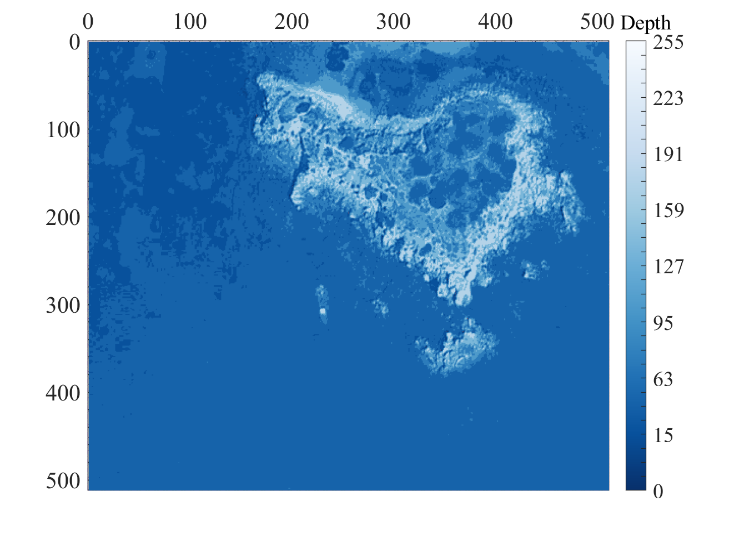

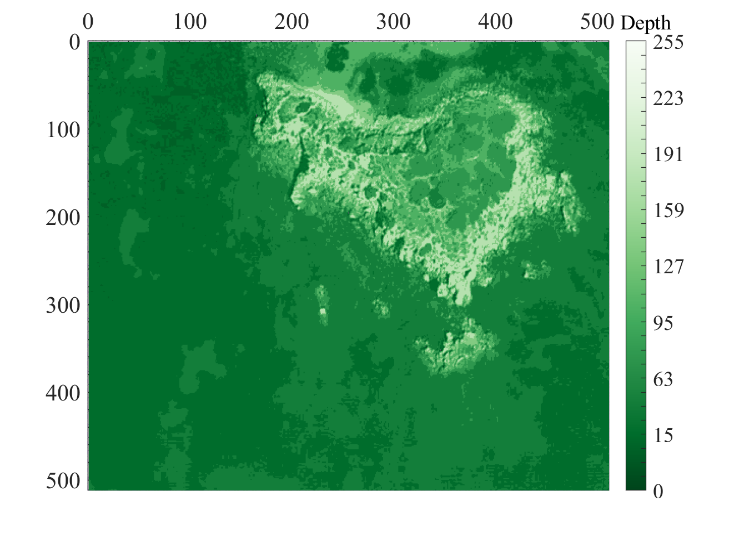


**Fig. 19** The 3D histograms and the projection for RUN.

Appendix E


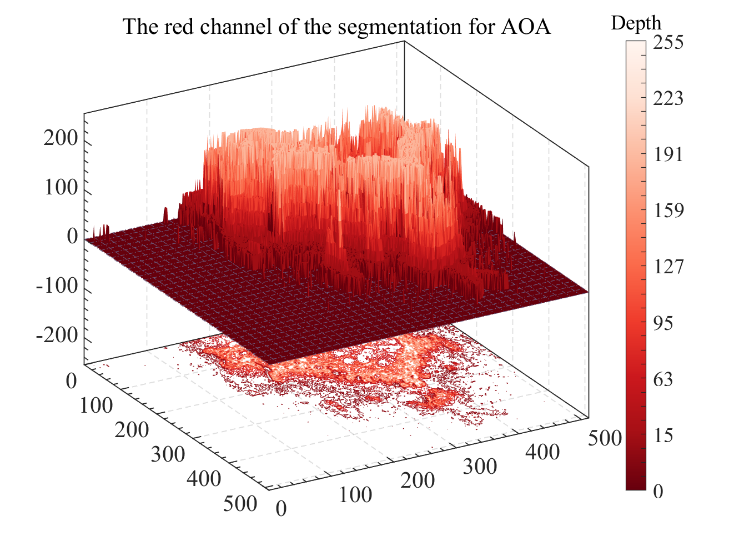

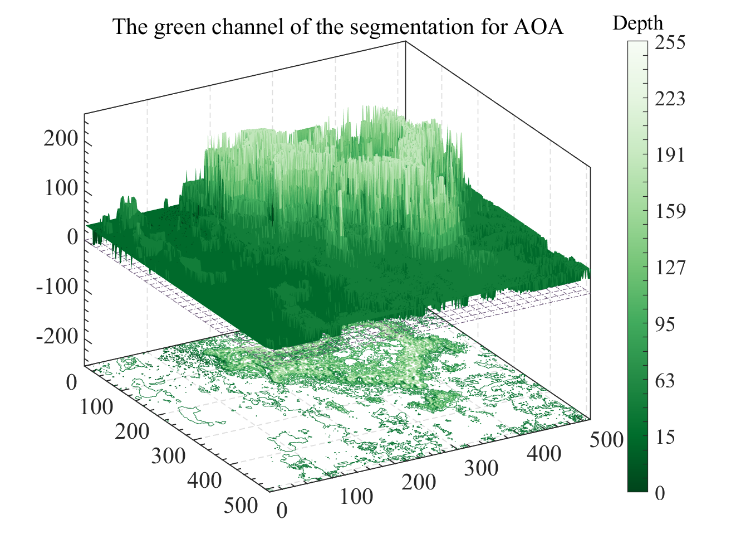

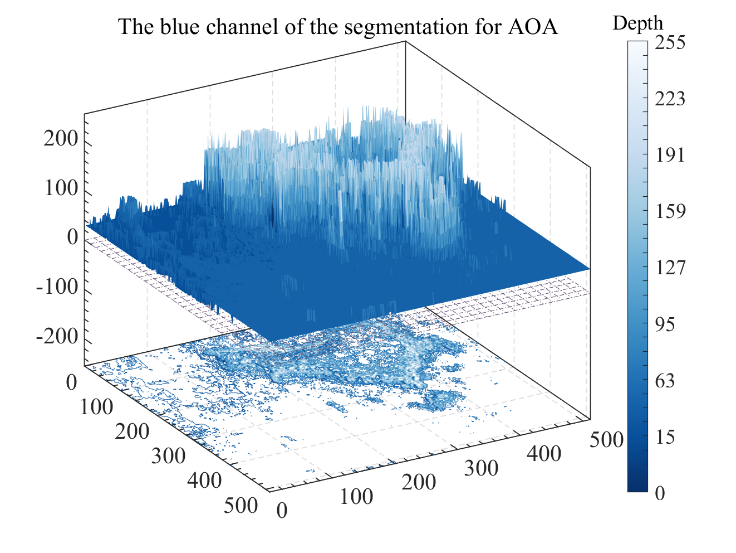

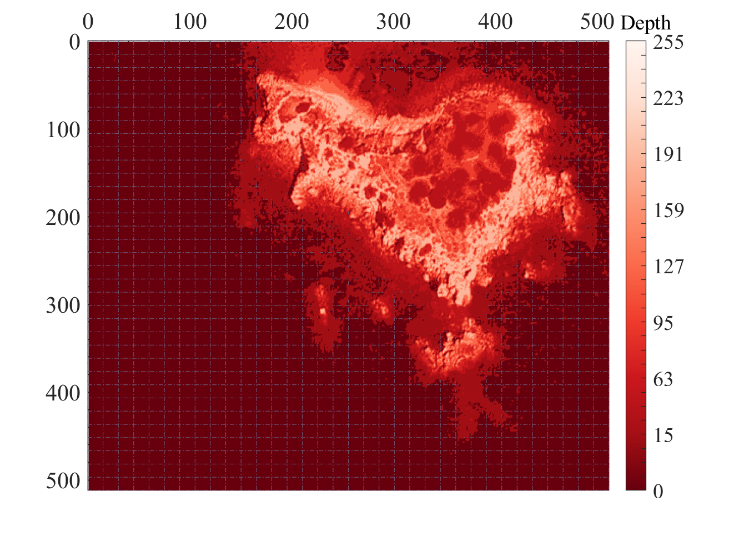

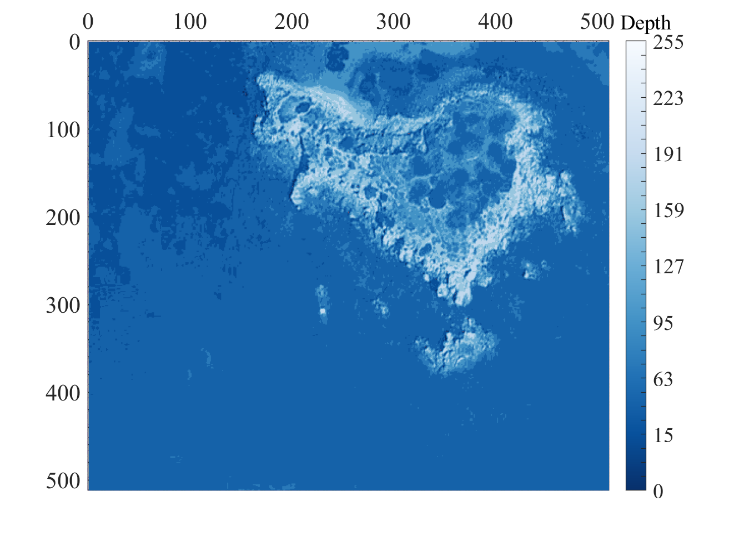

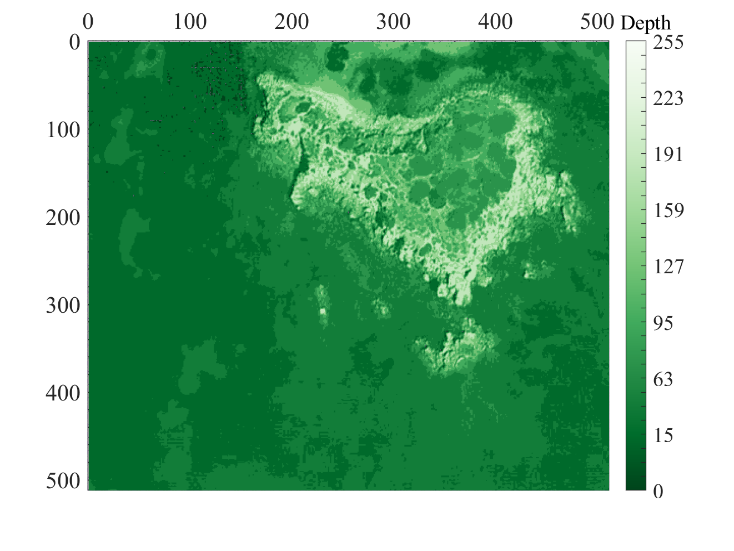


**Fig. 20** The 3D histograms and the projection for AOA.

Appendix F


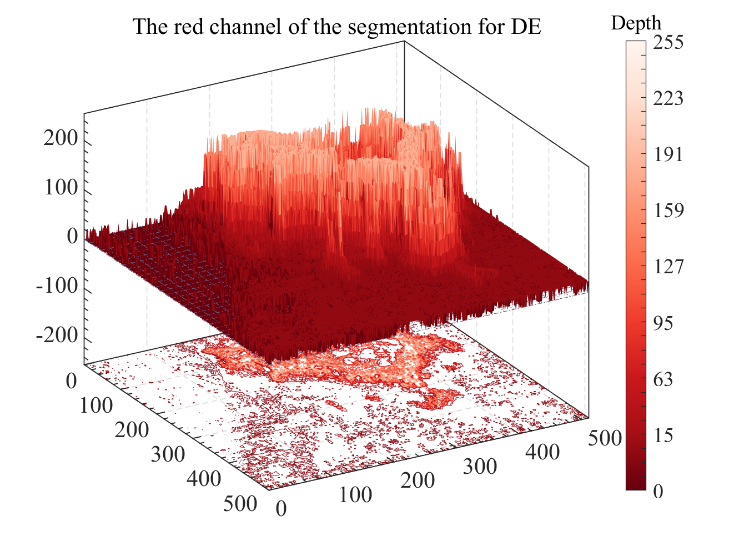

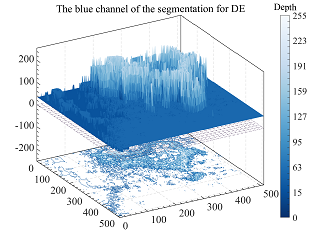

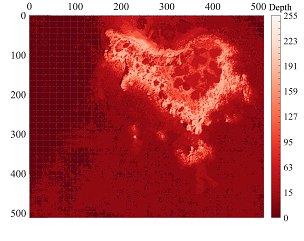

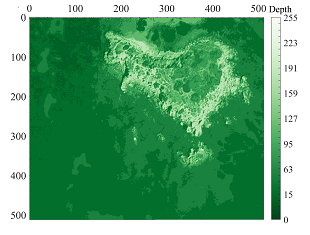

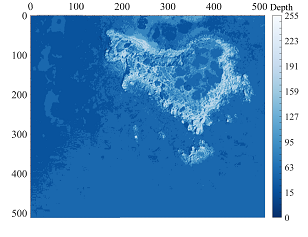

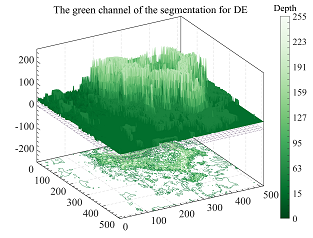


**Fig. 21** The 3D histograms and the projection for DE.

Appendix G


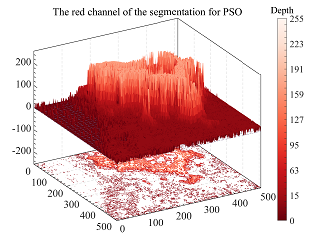

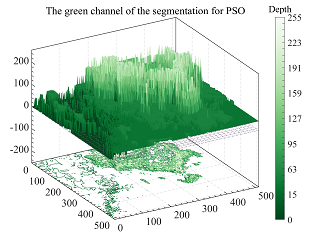

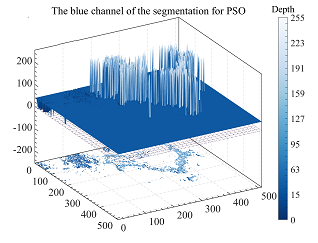

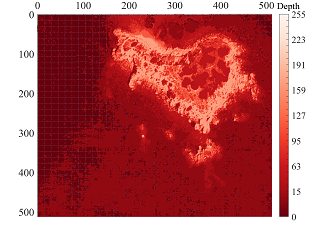

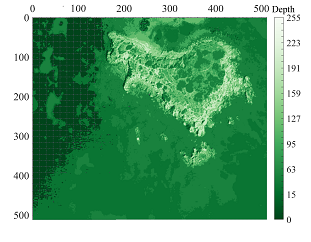

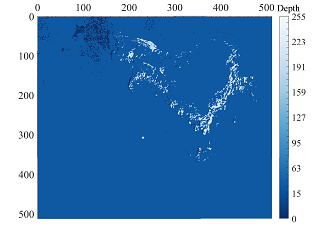


**Fig. 22** The 3D histograms and the projection for PSO.

s
