# Supplementary material for: An improved golden jackal optimization for multilevel thresholding image segmentation
Source: PLoS One. 2023 May 5;18(5):e0285211. doi: 10.1371/journal.pone.0285211 (PMC10162520; doi:10.1371/journal.pone.0285211)
Supplement: S1 File — (ZIP) [file pone.0285211.s002.zip › CEC/CEC22/CEC2022 TR.pdf]

Problem Definitions and Evaluation Criteria for the CEC 2022 Special Session and Competition on Single Objective Bound Constrained Numerical Optimization

Abhishek Kumar[1], Kenneth V. Price[2], Ali Wagdy Mohamed[3,4], Anas A. Hadi[5], P. N. Suganthan[6]

[1]Department of Electrical Engineering, Indian Institute of Technology (BHU), Varanasi, Varanasi, 221005, India.

[2] Vacaville, California, USA.

[3]Operations Research Department, Faculty of Graduate Studies for Statistical Research, Cairo University, Giza 12613, Egypt.

[4]Department of Mathematics and Actuarial Science, School of Sciences & Engineering, The American University in Cairo, Egypt..

[5]Faculty of Computer Studies, Arab Open University, P. O. Box 84901 Riyadh 11681, Saudi Arabia.

[6]School of Electrical Electronic Engineering, Nanyang Technological University, Singapore

abhishek.kumar.eee13@iitbhu.ac.in, kvprice@pacbell.net, aliwagdy@gmail.com, anas1401@gmail.com, epnsugan@ntu.edu.sg

Technical Report

December 2021

Single objective optimization algorithms are the foundation upon which more complex methods, like multi-objective, niching, and constrained optimization algorithms are built. Consequently, improvements to single objective optimization algorithms are important because they can impact other domains as well. These algorithmic improvements depend in part on feedback from trials conducted with single objective benchmark functions, which themselves are the elemental building blocks for more complex tasks, like dynamic, niching, composition, and computationally expensive problems. As algorithms improve, ever more challenging functions must be developed. This interplay between methods and problems drives progress, so we have developed the CEC'22 Special Session on Real-Parameter Optimization to promote this symbiosis.

Improved methods and problems sometimes require updating traditional testing criteria. In recent years, many novel optimization algorithms have been proposed to solve the bound-constrained, single objective problems offered in the CEC'05[1], CEC'13[2], CEC'14[3], CEC'17[4], CEC'20[4], and CEC'21[6] special sessions on Real-Parameter Optimization. Considering the comments on the CEC'20 test suite, we organized this competition on real parameter single objective optimization.

Participants are required to send their results to the organizers in the format specified in this technical report. Based on these results, organizers will present a comparative analysis that includes statistical tests on convergence performance to compare algorithms with similar final solutions.

Participants may not explicitly use the equations of the test functions, e.g. to compute gradients. This competition also excludes surrogate and meta-models. Papers on novel concepts that help us to understand problem characteristics are also welcome. C, Python, and MATLAB codes for CEC'22 test suite can be downloaded from the website below:

https://github.com/P-N-Suganthan

1. Introduction to the CEC'22 Benchmark Suite

1.1. Some Definitions:

All test functions are minimization problems defined as follows:

$$\text{Min } f(\mathbf{x}),\ \mathbf{x} = [\mathrm{x}_1, \mathrm{x}_2, \ldots, \mathrm{x}_D]$$

D: number of dimensions.

$o = [o_{i1}, o_{i2}, \ldots, o_{iD}]$: the shifted global optimum (defined in "shift_data_x.txt"), which is randomly distributed in $[-80,80]^D$. All test functions are shifted to o and are scalable.

Search range: $[-100,100]^D$. For convenience, the same search ranges are defined for all test functions.

M_i: rotation matrix. Different rotation matrix is assigned to each function and each basic function.

Considering that linkages seldom exist among all variables in real-world problems, CEC'22 randomly divides variables into subcomponents. The rotation matrix for each set of subcomponents is generated from standard normally distributed entries by Gram-Schmidt ortho-normalization with condition number *c* that is equal to 1 or 2.

1.2. Summary of the CEC'21 Test Suite

	No.	Functions	F_i^*
Unimodal Function	1	Shifted and full Rotated Zakharov Function	300
Basic Functions	2	Shifted and full Rotated Rosenbrock's Function	400
	3	Shifted and full Rotated Expanded Schaffer's $f6$ Function	600
	4	Shifted and full Rotated Non-Continuous Rastrigin's Function	800
	5	Shifted and full Rotated Levy Function	900
Hybrid Functions	6	Hybrid Function 1 (N = 3)	1800
	7	Hybrid Function 2 (N = 6)	2000
	8	Hybrid Function 3 (N = 5)	2200
Composition Functions	9	Composition Function 1 (N = 5)	2300
	10	Composition Function 2 (N = 4)	2400
	11	Composition Function 3 (N = 5)	2600
	12	Composition Function 4 (N = 6)	2700
Search range: $[-100,100]^D$			

***Please Note: These problems should be treated as black-box problems. The explicit equations of the problems are not to be used.**

1.3. Definitions of the Basic Functions

1) Zakharov Function

$$f_1(\mathbf{x}) = \sum_{i=1}^{D} x_i^2 + \left(\sum_{i=1}^{D} 0.5x_i\right)^2 + \left(\sum_{i=1}^{D} 0.5x_i\right)^4 \quad (1)$$

2) Rosenbrock's Function

$$f_2(\mathbf{x}) = \sum_{i=1}^{D-1} \left(100\left(x_i^2 - x_{i+1}\right)^2 + (x_{i+1} - 1)^2\right) \quad (2)$$

3) Expanded Schaffer's Function

Schaffer's Function: $g(x, y) = 0.5 + \frac{\left(\sin^2\left(\sqrt{x^2+y^2}\right)-0.5\right)}{\left(1+0.001(x^2+y^2)\right)^2}$

$$f_3(\mathbf{x}) = \mathrm{g}(x_1, x_2) + \mathrm{g}(x_2, x_3) + \ldots + \mathrm{g}(x_{D-1}, x_D) + \mathrm{g}(x_D, x_1) \quad (3)$$

4) Rastrigin's Function

$$f_4(\mathbf{x}) = \sum_{i=1}^{D}\left(x_i^2 - 10\cos(2\pi x_i) + 10\right) \quad (4)$$

5) Levy Function

$$f_5(\mathbf{x}) = \sin^2(\pi w_1) + \sum_{i=1}^{D-1}(w_i - 1)^2\,[1 + 10\sin^2(\pi w_i - 1)] + (w_D - 1)^2\,[1 + \sin^2(2\pi w_D)]$$

where $w_i = 1 + \frac{x_i - 1}{4},\ \forall\ i = 1, \ldots, D$ (5)

6) Bent Cigar Function

$$f_6(\mathbf{x}) = x_i^2 + 10^6 \sum_{i=2}^{D} x_i^2 \quad (6)$$

7) HGBat Function

$$f_7(\mathbf{x}) = \left|\left(\sum_{i=1}^{D} x_i^2\right)^2 - \left(\sum_{i=1}^{D} x_i\right)^2\right|^{0.5} + \left(0.5\sum_{i=1}^{D} x_i^2 + \sum_{i=1}^{D} x_i\right)/\mathrm{D} + 0.5 \quad (7)$$

8) High Conditioned Elliptic Function

$$f_8(\mathbf{x}) = \sum_{i=1}^{D} (10^6)^{\frac{i-1}{D-1}} x_i^2 \quad (8)$$

9) Katsuura Function

$$f_9(\mathbf{x}) = \frac{10}{D^2} \prod_{i=1}^{D}\left(1 + i\sum_{j=1}^{32}\frac{|2^j x_i - \mathrm{round}(2^j x_i)|}{2^j}\right)^{\frac{10}{D^{1.2}}} - \frac{10}{D^2} \quad (9)$$

10) Happycat Function

$$f_{10}(\mathbf{x}) = \left|\sum_{i=1}^{D} x_i^2 - D\right|^{1/4} + \left(0.5\sum_{i=1}^{D} x_i^2 + \sum_{i=1}^{D} x_i\right)/\mathrm{D} + 0.5 \quad (10)$$

11) Expanded Rosenbrock's plus Griewangk's Function

$$f_{11}(\mathbf{x}) = f_{15}\left(f_2(x_1, x_2)\right) + f_{15}\left(f_2(x_2, x_3)\right) + \cdots + f_{15}\left(f_2(x_{D-1}, x_D)\right) + f_{15}\left(f_2(x_D, x_1)\right) \quad (11)$$

12) Modified Schwefel's Function

$$f_{12}(\mathbf{x}) = 418.9829 \times D - \sum_{i=1}^{D} g(z_i) \tag{12}$$

$$z_i = x_i + 4.209687462275036\text{E} + 002$$

$$g(z_i) = \begin{cases} z_i \sin\left(|z_i|^{1/2}\right), & \text{if } |z_i| \leq 500 \\ \left(500 - \text{mod}(z_i, 500)\right)\sin\left(\sqrt{|500 - \text{mod}(z_i, 500)|}\right) - \dfrac{(z_i - 500)^2}{10000D}, & \text{if } z_i > 500 \\ \left(\text{mod}(|z_i|, 500) - 500\right)\sin\left(\sqrt{|\text{mod}(|z_i|, 500) - 500|}\right) - \dfrac{(z_i + 500)^2}{10000D}, & \text{if } z_i < -500 \end{cases}$$

13) Ackley's Function

$$f_{13}(\mathbf{x}) = -20 \exp\left(-0.2\sqrt{\frac{1}{D}\sum_{i=1}^{D} x_i^2}\right) - \exp\left(\frac{1}{D}\sum_{i=1}^{D} \cos(2\pi x_i)\right) + 20 + e \tag{13}$$

14) Discus Function

$$f_{14}(\mathbf{x}) = 10^6 x_1^2 + \sum_{i=2}^{D} x_i^2 \tag{14}$$

15) Griewank's Function

$$f_{15}(\mathbf{x}) = \sum_{i=1}^{D} \frac{x_i^2}{4000} - \prod_{i=1}^{D} cos\left(\frac{x_i}{\sqrt{i}}\right) + 1 \tag{15}$$

16) Schaffer's F7 Function

$$f_{16}(\mathbf{x}) = \left[\frac{1}{D-1}\sum_{i=1}^{D-1}\left(\sqrt{s_i}.\left(\sin\left(50.0 s_i^{0.2}\right) + 1\right)\right)\right]^2, s_i = \sqrt{x_i^2 + x_{i+1}^2} \tag{16}$$

1.4. Definitions of the CEC'22 Test Suite

A. Basic Functions

1) Shifted and Rotated Zakharov Function

$$F_1(\mathbf{x}) = f_1(M(x - o_1)) + F_1^* \tag{16}$$

Properties:

- Unimodal
- Non-separable

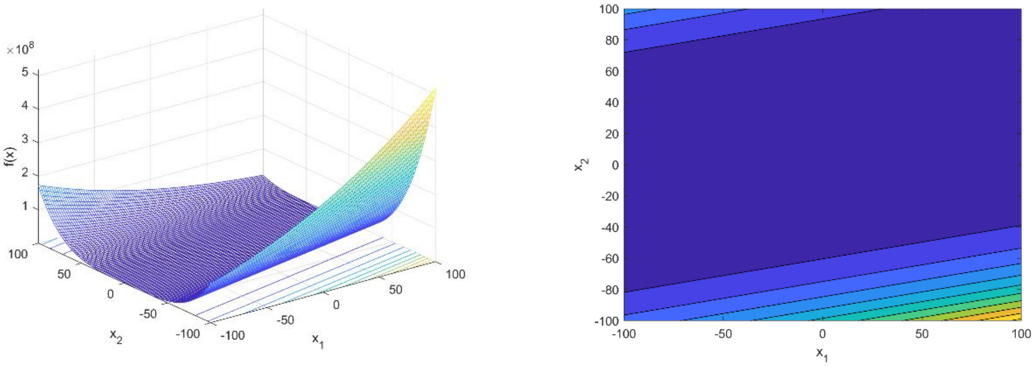

(a) 3-*D* map for 2-*D* function (b) Contour map for 2-*D* function

Figure 1 Shifted and Rotated Zakharov Function

2) Shifted and Rotated Rosenbrock's Function

$$F_2(\mathbf{x}) = f_2\left(M\left(\frac{2.048(x - o_2)}{100}\right) + 1\right) + F_2^* \tag{17}$$

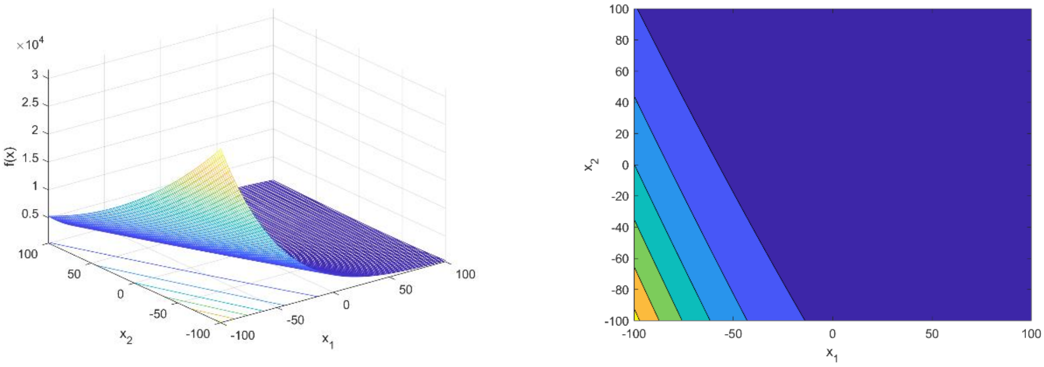

(a) 3-*D* map for 2-*D* function (b) Contour map for 2-*D* function

Figure 2 Shifted and Rotated Rosenbrock's Function

Properties:

- Multi-modal
- Non-separable
- Local optima's number is huge.

3) Shifted and full Rotated Expanded Schaffer's F7

$$F_3(\mathbf{x}) = f_3\left(M\left(\frac{0.5(x-o_3)}{100}\right)\right) + F_3^* \qquad (18)$$

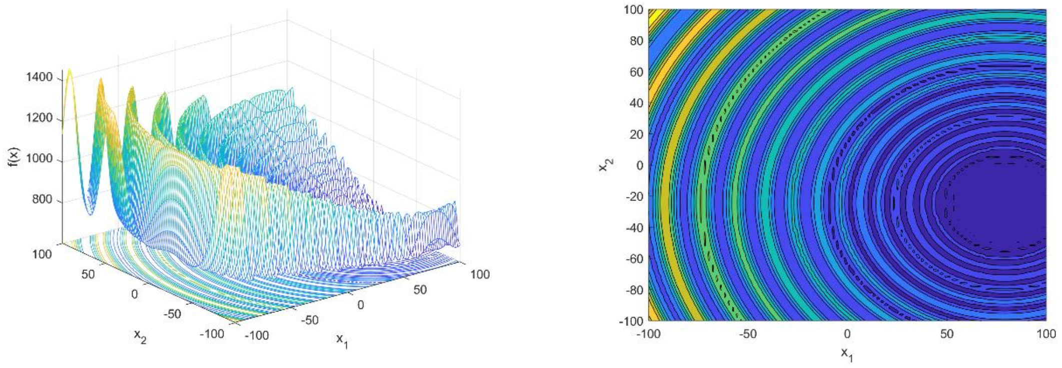

(a) 3-*D* map for 2-*D* function (b) Contour map for 2-*D* function

Figure 3 Shifted and full Rotated Expanded Schaffer's *f*6 Function

Properties:

- Multi-modal
- Non-separable
- Asymmetrical
- Local optima's number is huge.

4) Shifted and Rotated Non-Continuous Rastrigin's Function

$$F_4(\mathbf{x}) = f_4\left(M\left(\frac{5.12(x-o_4)}{100}\right)\right) + F_4^* \qquad (19)$$

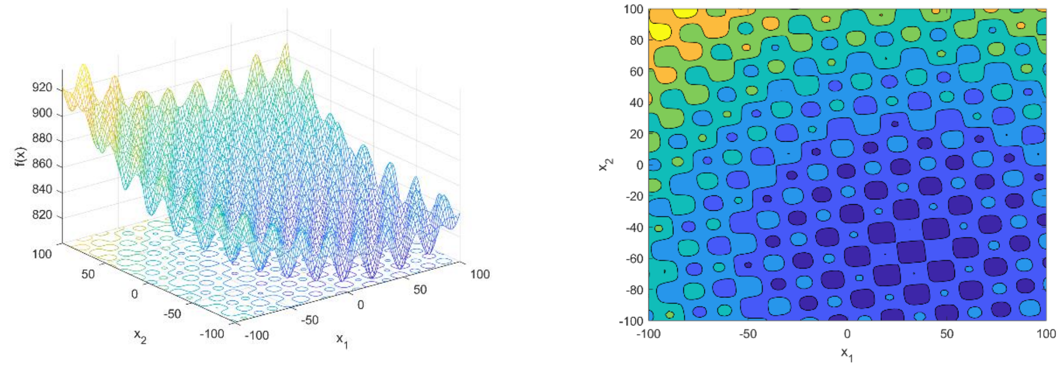

(a) 3-*D* map for 2-*D* function (b) Contour map for 2-*D* function

Figure 4 Shifted and full Rotated Non-Continuous Rastrigin's Function

Properties:

- Multi-modal
- Non-separable
- Asymmetrical
- Local optima's number is huge.

5) Shifted and Rotated Levy Function

$$\boldsymbol{F}_5(\mathbf{x}) = \boldsymbol{f}_5\left(\boldsymbol{M}\left(\frac{5.12(x-o_5)}{100}\right)\right) + \boldsymbol{F}_5^* \qquad (20)$$

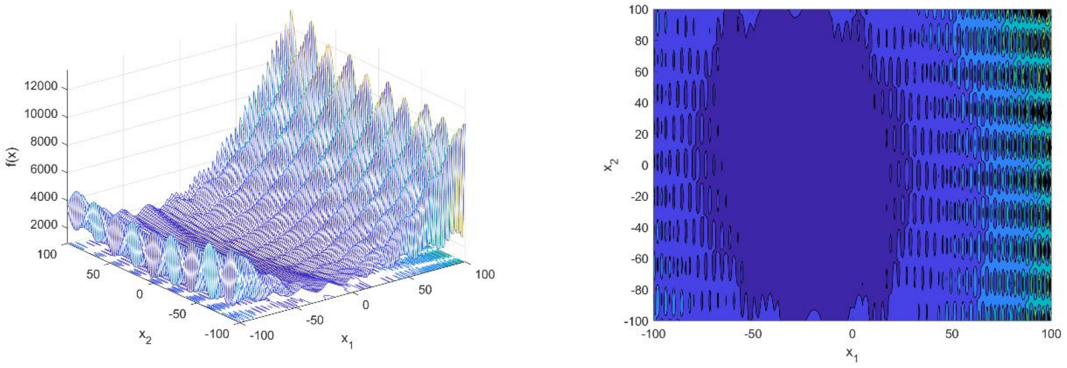

(a) 3-*D* map for 2-*D* function (b) Contour map for 2-*D* function

Figure 5 Shifted and Rotated Levy Function

Properties:

- Multi-modal
- Non-separable
- Local optima’s number is huge.

B. Hybrid Functions

In the real-world optimization problems, different subcomponents of the variables may have different properties[6]. In this set of hybrid functions, the variables are randomly divided into some subcomponents and then different basic functions are used for different subcomponents.

$$F(\mathbf{x}) = g_1(M_1 z_1) + g_2(M_2 z_2) + \cdots + g_N(M_N z_N) + F^*(\mathbf{x}) \qquad (21)$$

$F(\mathbf{x})$: hybrid function

$g_i(\mathbf{x})$: i^{th} basic function used to construct the hybrid function

N: number of basic functions

$z = [z_1, z_2, \dots, z_N]$

$z_1 = [y_{S_1}, y_{S_2}, \dots, y_{S_{n_1}}], z_2 = [y_{S_{n_1+1}}, y_{S_{n_1+2}}, \dots, y_{S_{n_1+n_2}}], \dots, z_N = [y_{S_{\sum_{i=1}^{N-1} n_i+1}}, y_{S_{\sum_{i=1}^{N-1} n_i+2}}, \dots, y_{S_D}]$

$y = x - o_i, S = \text{randperm}(1{:}D)$

p_i: used to control the percentage of $g_i(\boldsymbol{x})$

n_i: dimension for each basic function $\sum_{i=1}^{N} n_i = D$

$n_1 = \lceil p_1 D \rceil, n_2 = \lceil p_2 D \rceil, \dots, n_{N-1} = \lceil p_{N-1} D \rceil, n_N = D - \sum_{i=1}^{N-1} n_i$

Properties:

- Multi-modal or Unimodal, depending on the basic function
- Non-separable subcomponents
- Different properties for different variables subcomponents

6) Hybrid Function 1

$N = 3$

$p = [0.4, 0.4, 0.2]$

g_1: Bent Cigar Function f_6

g_2: HGBat Function f_7

g_3: Rastrigin's Function f_4

7) Hybrid Function 2

$N = 6$

$p = [0.1, 0.2, 0.2, 0.2, 0.2, 0.1, 0.2]$

$\sigma = [10,20,30]$

g_1: HGBat Function f_7

g_2: Katsuura Function f_9

g_3: Ackley's Function f_{13}

g_4: Rastrigin's Function f_4

g_5: Modified Schwefel's Function f_{12}

g_6: Schaffer's *F7* Function f_{16}

8) **Hybrid Function 3**

$N = 5$

$p = [0.3, 0.2, 0.2, 0.1, 0.2]$

g_1: Katsuura Function f_9

g_2: HappyCat Function f_{10}

g_3: Expanded Griewank's plus Rosenbrock's Function f_{11}

g_4: Modified Schwefel's Function f_{12}

g_5: Ackley's Function f_{13}

C. Composition Functions

$$F(\mathbf{x}) = \sum_{i=1}^{N}\{\omega_i^* [\lambda_i g_i(\mathbf{x}) + bias_i]\} + F^* \qquad (22)$$

$F(\mathbf{x})$: composition function

$g_i(\mathbf{x})$: i^{th} basic function used to construct the composition function

N: number of basic functions

o_i: new shifted optimum position for each $g_i(\mathbf{x})$, define the global and local optima's position

$bias_i$: defines which optimum is global optimum

σ_i: used to control each $g_i(x)$'s coverage range, a small σ_i gives a narrow range for that $g_i(x)$

λ_i: used to control each $g_i(x)$'s height

ω_i: weight value for each $g_i(x)$, calculated as below:

$$w_i = \frac{1}{\sqrt{\sum_{j=1}^{D}\left(x_j - o_{ij}\right)^2}} \exp\left(-\frac{\sum_{j=1}^{D}\left(x_j - o_{ij}\right)^2}{2D\sigma_i^2}\right) \qquad (23)$$

Then normalize the weight $\omega_i = w_i / \sum_{i=1}^{n} w_i$

So when $\mathbf{x} = \boldsymbol{o}_i$, $\omega_j = \begin{cases} 1 & j = i \\ 0 & j \neq i \end{cases}$ for $j = 1, 2, \ldots, N$, $f(\mathbf{x}) = bias_i + f^*$.

The local optimum which has the smallest bias value is the global optimum. The composition function merges the properties of the sub-functions better and maintains continuity around the global/local optima.

Functions $F_i' = F_i - F_i^*$ are used as g_i. In this way, the function values of global optima of g_i are equal to 0 for all composition functions in this report.

Please Note: All the basic functions that have been used in composition functions are shifted and rotated functions.

9) Composition Function 1

$N = 5$

$\sigma = [10,20,30,40,50]$

$\lambda = [1, 1e^{-6}, 1e^{-6}, 1e^{-6}, 1e^{-6}]$

$bias = [0, 200, 300, 100, 400]$

g_1: Rotated Rosenbrock's Function f_2

g_2: High Conditioned Elliptic Function f_8

g_3: Rotated Bent Cigar Function f_6

g_4: Rotated Discus Function f_{14}

g_5: High Conditioned Elliptic Function f_8

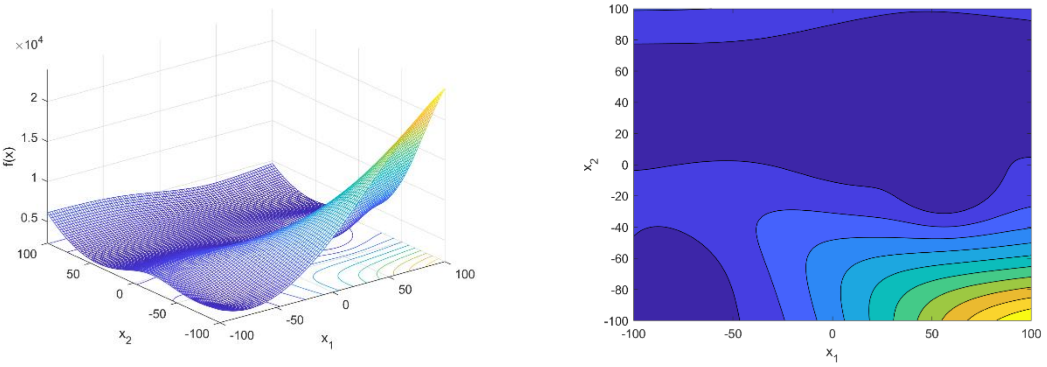

(a) 3-*D* map for 2-*D* function (b) Contour map for 2-*D* function

Figure 6 Composition Function 1

Properties:

- Multi-modal
- Non-separable
- Asymmetrical
- Different properties around different local optima

10) Composition Function 2

$N = 3$

$\sigma = [20,10,10]$

$\lambda = [1,1,1]$

$bias = [0,200,100]$

g_1: Rotated Schwefel's Function f_{12}

g_2: Rotated Rastrigin's Function f_4

g_3: HGBat Function f_7

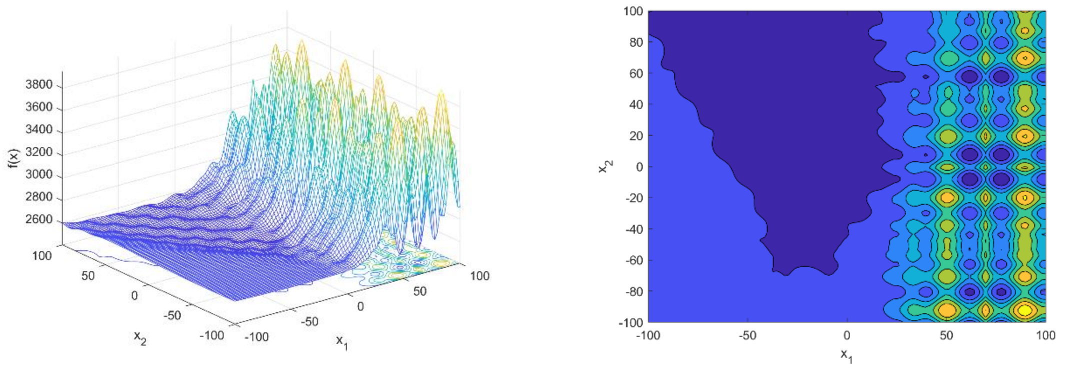

(a) 3-*D* map for 2-*D* function (b) Contour map for 2-*D* function

Figure 7 Composition Function 2

11) Composition Function 3

$N = 5$

$\sigma = [20,20,30,30,20]$

$\lambda = [1e^{-26}, 10, 1e^{-6}, 10, 5e^{-4}]$

$bias = [0, 200, 300, 400, 200]$

g_1: Expanded Schaffer's F6 Function f_3

g_2: Modified Schwefel's Function f_{12}

g_3: Griewank's Function f_{15}

g_4: Rosenbrock's Function f_2

g_5: Rastrigin's Function f_4

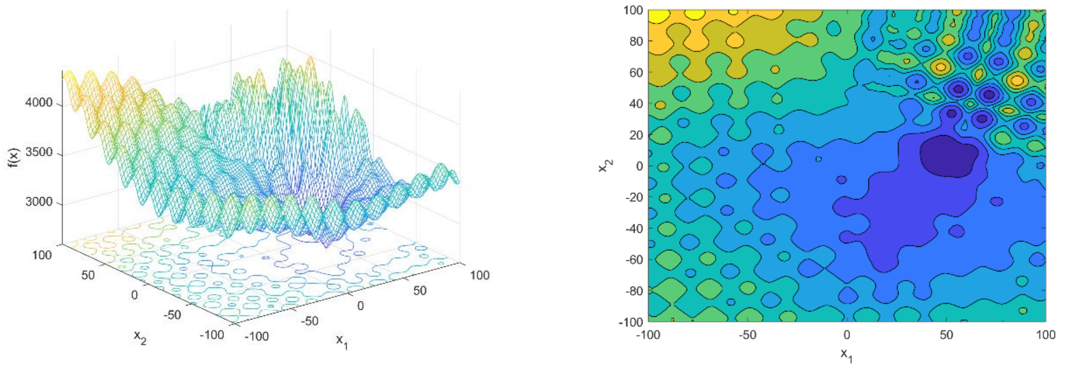

(a) 3-*D* map for 2-*D* function (b) Contour map for 2-*D* function

Figure 8 Composition Function 3

Properties:

- Multi-modal
- Non-separable
- Asymmetrical
- Different properties around different local optima

12) **Composition Function 4**

$N = 6$

$\sigma = [10,20,30,40,50,60]$

$\lambda = [10,10,2.5,1e^{-26},1e^{-6},5e^{-4}]$

$bias = [0,300,500,100,400,200]$

g_1: HGBat Function f_7

g_2: Rastrigin's Function f_4

g_3: Modified Schwefel's Function f_{12}

g_4: Bent Cigar Function f_6

g_5: High Conditioned Elliptic Function f_8

g_6: Expanded Schaffer's F6 Function f_3

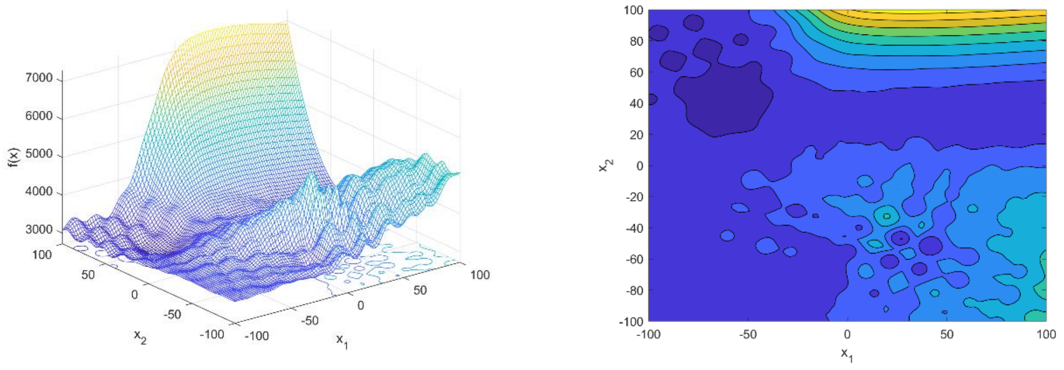

(a) 3-*D* map for 2-*D* function (b) Contour map for 2-*D* function

Figure 9 Composition Function 3

Properties:

- Multi-modal
- Non-separable
- Asymmetrical
- Different properties around different local optima

2. Experimental Settings and Evaluation Criteria

2.1. Experimental Settings

Problems: 12 minimization problems

Dimensions: D = 10 and 20

Runs / problem: 30

MaxFES:

	MaxFES
D = 10	200,000
D = 20	1,000,000

Search Range: $[-100,100]^D$

Initialization: Uniform random initialization within the search space. For fair comparison, 1000 uniform random seed have already been generated and stored in `'input_data\Rand_Seeds.txt'` file and the random seed for each run is based on four factors: Problem size (D), Function No. (func_no), Runs, and Run Id(run_id) according to:

```
seed_ind=(problem_size/10*func_no*Runs+run_id)-Runs;
seed_ind=mod(seed_ind,1000)+1;
run_seed=Rand_Seeds(seed_ind);
```

Matlab users can use:

```
rng(run_seed,'twister');
```

Global Optimum: All problems have the global optimum within the given bounds and there is no need to search outside of the given bounds for these problems.

$$F_i(\mathbf{x}^*) = F_i(\mathbf{o}_i) = F_i^*$$

Termination: Terminate when reaching *MaxFES* or when the error value is smaller than 10^{-8}.

2.2. Results Record

1) For each run, record the function error value $(F_i(\mathbf{x}) - F_i(\mathbf{x}^*))$ after $\left\lfloor D^{\frac{k}{5}-3} MaxFES \right\rfloor$ function evaluations (k = 0, 1, 2, 3, ... , 15). If a trial terminates by reaching an error value of 10^{-8} before reaching *MaxFES*, then record 10^{-8} for the remaining entries.
2) **New this year:** In addition, record FE_{term} *as the 17th entry for each run*, where FE_{term} is the number of function evaluations when a run terminates. For trials that do not reach 10^{-8}, record *MaxFES*; otherwise, enter the number of function evaluations at which the error value first became smaller than 10^{-8}.

For example, in problems with D = 10, record the function error value after $\left\lfloor 10^{\frac{0}{5}-3} \times 200{,}000 \right\rfloor \left\lfloor 10^{\frac{1}{5}-3} \times 200{,}000 \right\rfloor \left\lfloor 10^{\frac{2}{5}-3} \times 200{,}000 \right\rfloor \ldots \left\lfloor 10^{\frac{15}{5}-3} \times 200{,}000 \right\rfloor$ for each run. If, for example, the trial reached an error value of 10^{-8} after k = 9 and before k = 10 (i.e. before D^{-1}*$MaxFES$ = 20,000 function evaluations), then enter: 10^{-8} for entries: k = 10, 11, 12, 13, 14, 15 and 16. In addition, append

the record to make FE_{term} the 17th entry. If the trial fails to reach 10^{-8}, then record the error values at $k = 0, 1\ldots, 15$) and record *MaxFES* as the 17th entry.

3) Sort the error values achieved after *MaxFES* in 30 runs from the smallest (best) to the largest (worst) and present the best, worst, mean, median and standard variance values of function error values for the 30 runs.

4) Algorithm Complexity

a) Run the test program below:

$x = 0.55$
for i = 1: 200000
$x = x + x; x = x/2; x = x * x, x = sqrt(x); x = log(x); x = exp(x); x = x/(x + 2);$
end
Computing time for the above = *T0*;

b) Evaluate the computing time just for Function 1. For 200000 evaluations of a certain dimension *D*, it gives *T1*;
c) The complete computing time for the algorithm with 200000 evaluations of the same *D* dimensional Function 1 is *T2*.
d) Execute step c five times and get five *T2* values. $T2 = mean(T2)$

The complexity of the algorithm is reflected by: *T2*, *T1*, *T0*, and $(T2 - T1)/T0$.

The algorithm complexities are calculated on 10, 20 dimensions, to show the algorithm complexity's relationship with dimension. Also provide sufficient details on the computing system and the programming language used. In step c, we execute the complete algorithm five times to accommodate variations in execution time due adaptive nature of some algorithms.

Please Note: Similar programming styles should be used for all *T0*, *T1* and *T2*.

(For example, if *m* individuals are evaluated at the same time in the algorithm, the same style should be employed for calculating *T1*; if parallel calculation is employed for calculating *T2*, the same way should be used for calculating *T0* and *T1*. In other words, the complexity calculation should be fair.)

5) Parameters

Participants must not search for a distinct set of parameters for each problem/dimension/etc. Please provide details on the following whenever applicable:

a) All parameters to be adjusted

b) Corresponding dynamic ranges

c) Guidelines on how to adjust the parameters

d) Estimated cost of parameter tuning in terms of number of FEs

e) Actual parameter values used.

6) Encoding

If the algorithm requires encoding, then the encoding scheme should be independent of the specific problems and governed by generic factors such as the search ranges.

7) Results Format

The participants are required to send the final results as the following format to the organizers and the organizers will present an overall analysis and comparison based on these results.

Create one txt document with "AlgorithmName_FunctionNo._D.txt" for each test function and for each dimension.

For example, DE results for test function 5, and *D* = 10, the file name should be "DE_5_10.txt".

Then save the results matrix (***the gray shadowing part***) as Table I in the file:

Table I Information Matrix for *D* Dimensional Function X with the configuration Y.

***.txt	Run1	Run2	…	Run30
Function error values when FES = $\lfloor D^{\frac{0}{5}-3} MaxFES \rfloor$				
Function error values when FES = $\lfloor D^{\frac{1}{5}-3} MaxFES \rfloor$				
Function error values when FES = $\lfloor D^{\frac{2}{5}-3} MaxFES \rfloor$				
Function error values when FES = $\lfloor D^{\frac{3}{5}-3} MaxFES \rfloor$				
… …				
Function error values when FES = $\lfloor D^{\frac{14}{5}-3} MaxFES \rfloor$				
Function error values when FES = $\lfloor D^{\frac{15}{5}-3} MaxFES \rfloor$				
Number of Function Evaluations upon termination (FE_{term})				

Since there are 12 functions and 2 dimensions, 12*2 files should be zipped and sent to the organizers. Each file contains a 17*30 matrix.

Notice: All participants are allowed to improve their algorithms further after submitting the initial version of their papers to CEC2022. And they are required to submit their results in the introduced format to the organizers after submitting the **final** version of paper as soon as possible.

8) Results Template

Language: MATLAB 2020b

Algorithm: Differential Evolution (DE)

Please Notice: Considering the length limit of the paper, only Error Values Achieved with *MaxFES* are need to be listed. While the authors are required to send all results to the organizers for a better comparison among the algorithms.

Table II Results for 10 D

Func.	Best	Worst	Median	Mean	Std
1					
2					
3					
4					
5					
8					
9					
10					
11					
12					

Table III Results for 20D

Func.	Best	Worst	Median	Mean	Std
1					
2					
3					
4					
5					
8					
9					
10					
11					
12					

Algorithm Complexity

Table IV Computational Complexity

	T0	T1	$T2$	$(T2-T1)/T0$
$D = 10$				
$D = 20$				

2.3. Evaluation Criteria

This year's evaluation criterion rewards not only accuracy, but also speed. It is based on the Wilcoxon rank-sum test [11] (aka the Mann-Whitney U-test [12]) and the observation that trials can be ranked from best to worst when they terminate upon reaching either the minimum error value (10^{-8}) or the maximum number of function evaluations MaxFES (Fig. 10).

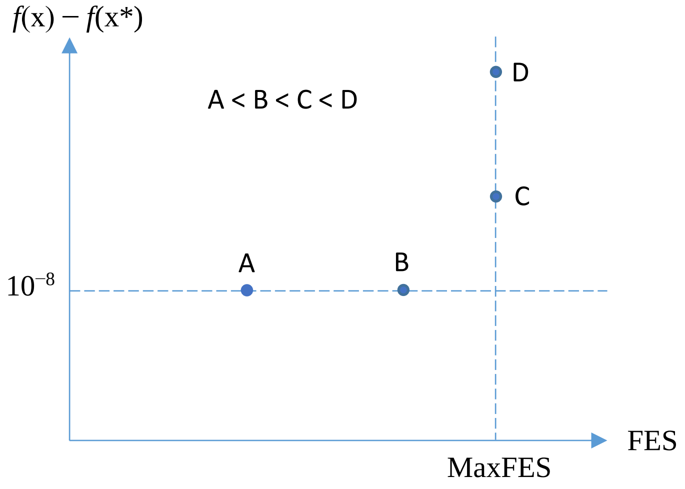

Figure 10 Trials sampled on the terminal axes can be unambiguously ordered from best to worst. Trial A is the best, since smaller is better.

Suppose that $t_{i,j,k}$ is the ith trial from the jth algorithm optimizing the kth function. To compute the algorithm scores for function k, rank all trials $t_{i,j,k}$, $i = 1, 2\ldots, n$, $j = 1, 2\ldots, m$, where n is the number of trials (30) and m is the number of algorithms. Assign the best trial the highest rank (nm). Resolve ties by assigning average ranks to identical trials. Once trials have been ranked, compute each algorithm's score for function k as the *sum of its ranks* minus the correction term $n(n + 1)/2$. An algorithm's *final score* is the sum of its *function scores*.

Figure 11 provides an example with three algorithms, P, Q and R, each of which ran four trials on one function. Table V illustrates how algorithm scores are computed from Fig. 11.

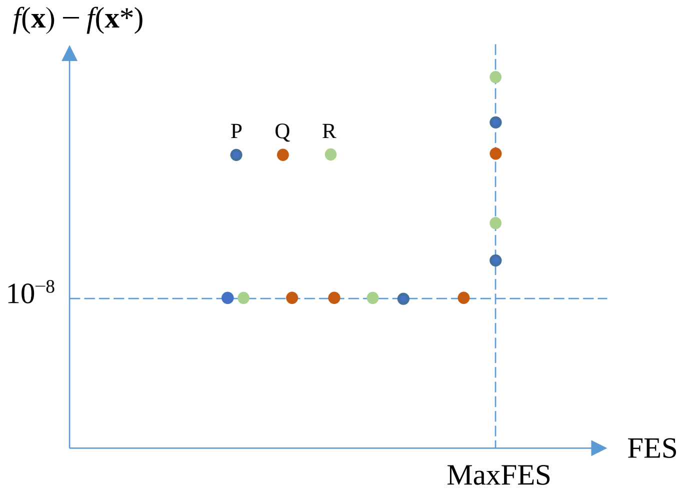

Figure 11 Three algorithms, P, Q and R, run four trials each. Five trials terminate when they reach MaxFES, while seven terminate when they reach an error value of 10^{-8}. All twelve trials can be ordered from best to worst.

Table V

Function scores for algorithms P, Q and R are derived by summing their ranks. Algorithm Q wins with a score of 18. The correction factor for four trials is 4·5/2 = 10. "SR" = sum of ranks.

trial:	p	r	q	q	r	p	q	p	r	q	p	r	SR	SR − 10
rank:	12	11	10	9	8	7	6	5	4	3	2	1		
P	12					7		5			2		26	16
Q			10	9			6			3			28	18
R		11			8				4			1	24	14

The score obtained by ranking trials is also the number of times that an algorithm wins, i.e. has the better trial, when all of its trials are compared to all trials from all other algorithms (on the same function). In a competition between just two algorithms, the score reduces to the Mann-Whitney U-statistic.

.

References

[1] P. N. Suganthan, N. Hansen, J. J. Liang, K. Deb, Y. P. Chen, A. Auger & S. Tiwari, "Problem Definitions and Evaluation Criteria for the CEC 2005 Special Session on Real-Parameter Optimization," Technical Report, Nanyang Technological University, Singapore, May 2005 and KanGAL Report #2005005, IIT Kanpur, India, 2005.

[2] J. J. Liang, B. Y. Qu, P. N. Suganthan, Alfredo G. Hernández-Díaz, "Problem Definitions and Evaluation Criteria for the CEC 2013 Special Session and Competition on Real-Parameter Optimization", Computational Intelligence Laboratory, Zhengzhou University, Zhengzhou China and Technical Report, Nanyang Technological University, Singapore, January 2013.

[3] J. J. Liang, B. Y. Qu, P. N. Suganthan, "Problem Definitions and Evaluation Criteria for the CEC 2014 Special Session and Competition on Real-Parameter Optimization", Computational

Intelligence Laboratory, Zhengzhou University, Zhengzhou China and Technical Report, Nanyang Technological University, Singapore, January 2014.

[4] N. H. Awad, M. Z. Ali, P. N. Suganthan, J. J. Liang, B. Y. Qu, " Problem Definitions and Evaluation Criteria for the CEC 2017 Special Session and Competition on Single Objective Real-Parameter Numerical Optimization", Computational Intelligence Laboratory, Zhengzhou University, Zhengzhou China and Technical Report, Nanyang Technological University, Singapore, October 2016.

[5] C. T. Yue, K. V. Price, P. N. Suganthan, J. J. Liang, M. Z. Ali, B. Y. Qu, N. H. Awad, & P.P Biswas, "Problem Definitions and Evaluation Criteria for the CEC 2020 Special Session and Competition on Single Objective Bound Constrained Numerical Optimization", Technical Report. Rep., Zhengzhou University and Nanyang Technological University, 2019.

[6] A. M. Wagdy, A. A. Hadi, A. A. Mohamed, P. Agrawal, A. Kumar, & P. N. Suganthan, "Problem Definitions and Evaluation Criteria for the CEC 2021 Special Session and Competition on Single Objective Bound Constrained Numerical Optimization", Technical Report. Rep., Zhengzhou University and Nanyang Technological University, 2020.

[7] Xiaodong Li, Ke Tang, Mohammad N. Omidvar, Zhenyu Yang, and Kai Qin, "Benchmark Functions for the CEC'2013 Special Session and Competition on Large-Scale Global Optimization", Technical Report, 2013.

[8] N. Hansen, A. Auger, S. Finck, and R. Ros. 2009. Real-Parameter Black-Box Optimization Benchmarking 2009: Experimental setup. Technical Report. INRIA. http://coco.gforge.inria.fr

[9] J. J. Liang, S. Baskar, P. N. Suganthan and A. K. Qin, Performance Evaluation of Multiagent Genetic Algorithm, Natural Computing, volume 5, pages 83–96, (2006).

[10] JJ Liang, PN Suganthan, K Deb. Novel composition test functions for numerical global optimization, Proceedings of 2005 IEEE Swarm Intelligence Symposium, SIS 2005, 68-75.

[11] F. Wilcoxon, "Individual Comparisons by Ranking Methods," *Biometrics Bulletin*, vol. 1, no. 6, [International Biometric Society, Wiley], 1945, pp. 80–83, https://doi.org/10.2307/3001968

[12] Mann, Henry B., Whitney, Donald R. (1947). "On a Test of Whether One of Two Random Variables is Stochastically Larger than the Other," *Annals of Mathematical Statistics*, **18** (1), 1947, pp. 50–60. https://doi:10.1214/aoms/1177730491 . MR 0022058. Zbl 0041.26103
